# Chronic Intake of Japanese *Sake* Mediates Radiation-Induced Metabolic Alterations in Mouse Liver

**DOI:** 10.1371/journal.pone.0146730

**Published:** 2016-01-11

**Authors:** Tetsuo Nakajima, Guillaume Vares, Bing Wang, Mitsuru Nenoi

**Affiliations:** Research Center for Radiation Protection, National Institute of Radiological Sciences, Chiba, Japan; Georgetown University, UNITED STATES

## Abstract

*Sake* is a traditional Japanese alcoholic beverage that is gaining popularity worldwide. Although *sake* is reported to have beneficial health effects, it is not known whether chronic *sake* consumption modulates health risks due to radiation exposure or other factors. Here, the effects of chronic administration of *sake* on radiation-induced metabolic alterations in the livers of mice were evaluated. *Sake* (*junmai-shu)* was administered daily to female mice (C3H/He) for one month, and the mice were exposed to fractionated doses of X-rays (0.75 Gy/day) for the last four days of the *sake* administration period. For comparative analysis, a group of mice were administered 15% (v/v) ethanol in water instead of *sake*. Metabolites in the liver were analyzed by capillary electrophoresis-time-of-flight mass spectrometry one day following the last exposure to radiation. The metabolite profiles of mice chronically administered *sake* in combination with radiation showed marked changes in purine, pyrimidine, and glutathione (GSH) metabolism, which were only partially altered by radiation or *sake* administration alone. Notably, the changes in GSH metabolism were not observed in mice treated with radiation following chronic administration of 15% ethanol in water. Changes in several metabolites, including methionine and valine, were induced by radiation alone, but were not detected in the livers of mice who received chronic administration of *sake*. In addition, the chronic administration of *sake* increased the level of serum triglycerides, although radiation exposure suppressed this increase. Taken together, the present findings suggest that chronic *sake* consumption promotes GSH metabolism and anti-oxidative activities in the liver, and thereby may contribute to minimizing the adverse effects associated with radiation.

## Introduction

Human health is influenced by lifestyle choices, including exercise, diet, and tobacco use. Among these factors, alcohol consumption is related with numerous health risks and diseases, such as cardiovascular diseases and cancer [1]. The liver is particularly susceptible to alcohol-related disease [2] and higher alcohol consumption is a major cause of hepatocellular carcinoma [3]. In addition, when combined with other risk factors, such as tobacco, alcohol may have synergistic effects on health [4–7].

Radiation is also a significant health risk factor, and is associated with both acute and chronic effects depending on the quality and dose of radiation. The effects of radiation on human health may be mediated, at least in part, by lifestyle-related factors, such as diet [8–10]. For example, the findings from epidemiological studies in nuclear plant workers suggest that lifestyle-related factors, including alcohol consumption, influence the adverse effects of radiation [11,12]. However, how alcohol drinking custom mediates radiation effects remains unclear.

Many types of alcohol beverages are consumed worldwide. Although alcohol in general has been shown to adversely affect human health [2,13], certain beverages, including beer and *sake*, have been demonstrated to have anti-mutagenic activities [14]. Recently, it was reported that *sake*, a popular Japanese alcoholic beverage, has protective effects against acute radiation [15]. However, it remains unknown whether chronic *sake* consumption mediates such radiation-induced effects.

The liver is the main organ involved in detoxification of harmful substances, including alcohol [16] and is susceptible to radiation damage [8,10,17]. Alcohol is metabolized in the liver, and the resulting metabolic byproducts can impair liver function and cause tissue damage [18]. For this reason, liver metabolites are useful indicators of health status.

Here, the influence of chronic *sake* consumption on radiation-induced effects, particularly those related to the alteration of liver metabolites, was evaluated in mice using a metabolic approach.

## Materials and Methods

### Animal care

Seven week-old female C3H/He mice were purchased from the Japan SLC Co. (Hamamatsu, Japan). Mice were housed for seven weeks to allow for adaptation before performing experiments. Mice were typically allowed access to water and standard laboratory chow (MB-1, Funabashi Farm Co., Japan) ad libitum. The major components of MB-1 (gross energy, 4.28 Kcal/g) were as follows: total carbohydrate, 54.4%; proteins, 24.2%; fat, 4.4%; fibers, 3.6%; moisture, 8.0% and ash, 5.4%. All animal studies were reviewed and approved by The Institutional Animal Care and Use Committee of the National Institute of Radiological Sciences (NIRS), and were performed in strict accordance with the NIRS *Guidelines for the Care and Use of Laboratory Animals*. A total of 4 or 5 mice were used in each administration group (*sake* or ethanol). Measurements of whole body weight, organ weight, and metabolic markers were performed using 3 (corresponding to metabolome analysis samples) or 4 mice in each administration group in two independent experiments.

### *Sake* administration

*Sak*e *(junmai-shu*; Daishichi Sake Brewery, Nihonmatsu, Japan) produced with rice polished to 69% and containing 15% (v/v) alcohol was used to examine the effects of *sake* on the liver metabolome in this study. As a comparative study to examine the effects of ethanol, 15% (v/v) special grade ethanol (99.5%) (Wako Pure Chemical Industries, Ltd.) in water was administered to mice instead of *sake*.

*Sake* (0.2 or 0.6 ml per 23 g body weight, corresponding to 0.009 ml/g or 0.026 ml/g body weight, respectively) was administrated to mice using a feeding needle every morning for one month (30–31 days). The body weights of mice were measured every evening and used for calculation of the *sake* intake amounts the next morning. In comparative experiments using 15% ethanol in water, 0.6 ml of ethanol solution was administered to mice. A control group was administered the same volume of drinking water in place of *sake*. Before administering the test solutions in the morning, food and water were withheld from mice from the previous evening. Mice in the irradiated group were irradiated on the last four days of the *sake*-administration period, as described in the following section. A total of 4 or 5 mice were used in each experimental group for sake and ethanol. S1 and S2 Figs are representative graphs showing changes in the body weights of mice administered either sake or 15% ethanol administration. After giving the mice 0.6 ml *sake* per day for one month, the gross appearance of mice was normal and no changes in weight were observed compared to controls; therefore, the following experiments were performed by administering 0.6 ml *sake* to mice, unless otherwise noted.

### Irradiation

Mice orally administered *sake* every day for one month (0.6 ml per 23 g body weight) were treated with fractionated irradiation (0.75 Gy/day). The irradiation was performed once a day at a dose rate of 0.85 Gy/min during the last four days of the *sake*- or ethanol- administration period immediately after the administration. Irradiation was performed using a Pantak 320S machine (Shimadzu, Japan) equipped with a 0.50-mm Al + 0.50-mm Cu filter and operated at 200 kVp and 20 mA. An exposure rate meter (AE-1321M; Applied Engineering Inc., Japan) was used for the dosimetry. Blood and organ collection was performed one day after the last irradiation. Mice were anesthetized by inhalation of gaseous isoflurane (Pfizer, Tokyo, Japan) and blood was collected for serum preparation. The mice were then euthanized by cervical dislocation, and liver, thymus and spleen samples were collected for analysis.

### Metabolome analysis

The liver tissue from three mice from the *sake* and ethanol administration groups was used for metabolome analysis. A portion of the collected liver tissue was immediately frozen in liquid nitrogen for metabolite analysis. After thawing, the samples (approximately 50 mg) were homogenized in 1800 μl of 50% acetonitrile solution (v/v) containing internal standards (Human Metabolome Technologies (HMT), Inc., Tsuruoka, Japan) at concentrations of 5 and 20 μM for anion and cation modes, respectively. The homogenized samples were centrifuged (2,300 × g) for 5 min at 4°C and the separated upper layer was ultrafiltered (9,100 × g for 120 min at 4°C) using an ultrafiltration tube with a molecular weight cut-off of 5 kDa. The filtrate was evaporated and dissolved in 50 μl Milli-Q water for analysis using a capillary electrophoresis-time-of-flight mass spectrometry (CE-TOFMS) system (Agilent Technologies). Cationic metabolites were diluted twofold in cation buffer solution (HMT, Inc.) and analyzed using a fused silica capillary tube (i.d. 50 μm × 80 cm). For the analysis, the samples were injected into the system at a pressure of 50 mbar over 10 sec. The CE voltage was set at 27 kV. Electro-spray ionization-mass spectrometry (ESI-MS) was conducted in the positive ion mode. The capillary voltage was set at 4000 V. The spectrometer was scanned from m/z 50–1000. Anionic metabolites were diluted fivefold in anion buffer solution (HMT, Inc.) and analyzed using a fused silica capillary tube (i.d. 50 μm × 80 cm). For the analysis, the samples were injected at a pressure of 50 mbar over 25 sec. The CE voltage was set at 30 kV. ESI-MS was conducted in the negative ion mode. The capillary voltage was set at 3500 V. The spectrometer was scanned from m/z 50–1000. The raw data obtained by CE-TOFMS were processed with MasterHands software (ver. 2.16.0.15; developed at Keio University, Japan), which automatically extracted peaks greater than 3 (Signal/Noise ratio), and various data, including the m/z ratio, migration times (MT), and peak area values, were collected. The relative area values were calculated based on the sample tissue weight and were adjusted for the analysis sensitivity. Each peak was aligned based on the MT and m/z value. Metabolites in the samples were identified by comparing the MT and m/z values with those of authentic standards (HMT metabolite library), in which differences of ± 0.5 min and ± 10 ppm were permitted. The estimated relative area values were subjected to principal component analysis (PCA). We used the technical services of the HMT Research Group (Tsuruoka, Japan) for the metabolome analysis by CE-TOFMS. PCA was performed using SampleStat ver.3.14 (HMT). Hierarchical clustering analysis (HCA) and heat mapping were performed using PeakStat ver3.18 (HMT). The identification of metabolites was performed using the HMT database by measuring standard metabolites. This method for identification has been used in many organisms and it is also accepted in liver samples [19, 20, 21]. Description of XA・・・or XC・・・ in the compound name in S3 and S4 Tables indicate unknown peaks that have been detected in samples from other organisms in the HMT database for metabolite identification.

### Free amino acid analysis

Free amino acid concentrations in *sake* were determined using an automated amino acid analysis system (JLC-500v2; JEOL Ltd., Japan). The assay samples were prepared by adding 22 ml of 0.1% 2-mercaptoethanol and 3 ml of 50% TCA solution to each 5-g sample of *sake*. After mixing, the resulting solutions were kept for 3 hours on ice, and were then centrifuged at 10,000 × g for 20 min at 4°C. After filtration of the supernatants through a No. 5A filter (Advantec), 1 N NaOH (70 μl) was added to 1 ml of filtrate, and the resulting solutions were diluted 1:3 (v/v) in the primary buffer of the analysis system. After filtration through a 0.45μm filter (DISMIC-13CP, Advantec), the samples were analyzed. All procedures were performed on ice. All analyses were performed by the NH (Nipponham) Foods Ltd. Research and Development Center (Tsukuba, Japan).

### Serum preparation and biochemical marker analysis

Collected blood was kept at room temperature for 90 min and was then centrifuged at 1,000 × g for 15 min at 20°C. The resulting supernatant was collected as serum and was stored at -80°C until needed for analysis. Metabolic markers in serum were analyzed using a Dri-Chem 7000V (Fuji Film, Japan).

### Statistical analysis

Changes in metabolites were examined statistically using Welch’s *t*-test. To draw biological inferences using factor loading in the principal component analysis (PCA), factor loading was defined as the correlation coefficient between the PC scores and variables [22]. Statistical testing for factor loading in PCA was performed based on the fact that for a correlation coefficient r, the statistic:

$$t=\frac{r\sqrt{n-2}}{\sqrt{1-r^{2}}}$$

has a *t*-distribution with (n−2) degrees of freedom. Metabolites that has statistically significant (P<0.01) correlation between the PC score and the relative area value were selected, and characters of the metabolites were evaluated. All other statistical analyses were performed by the unpaired *t*-test.

## Results and Discussion

### Effects of *sake* and radiation on organ weight

**Fig 1:**
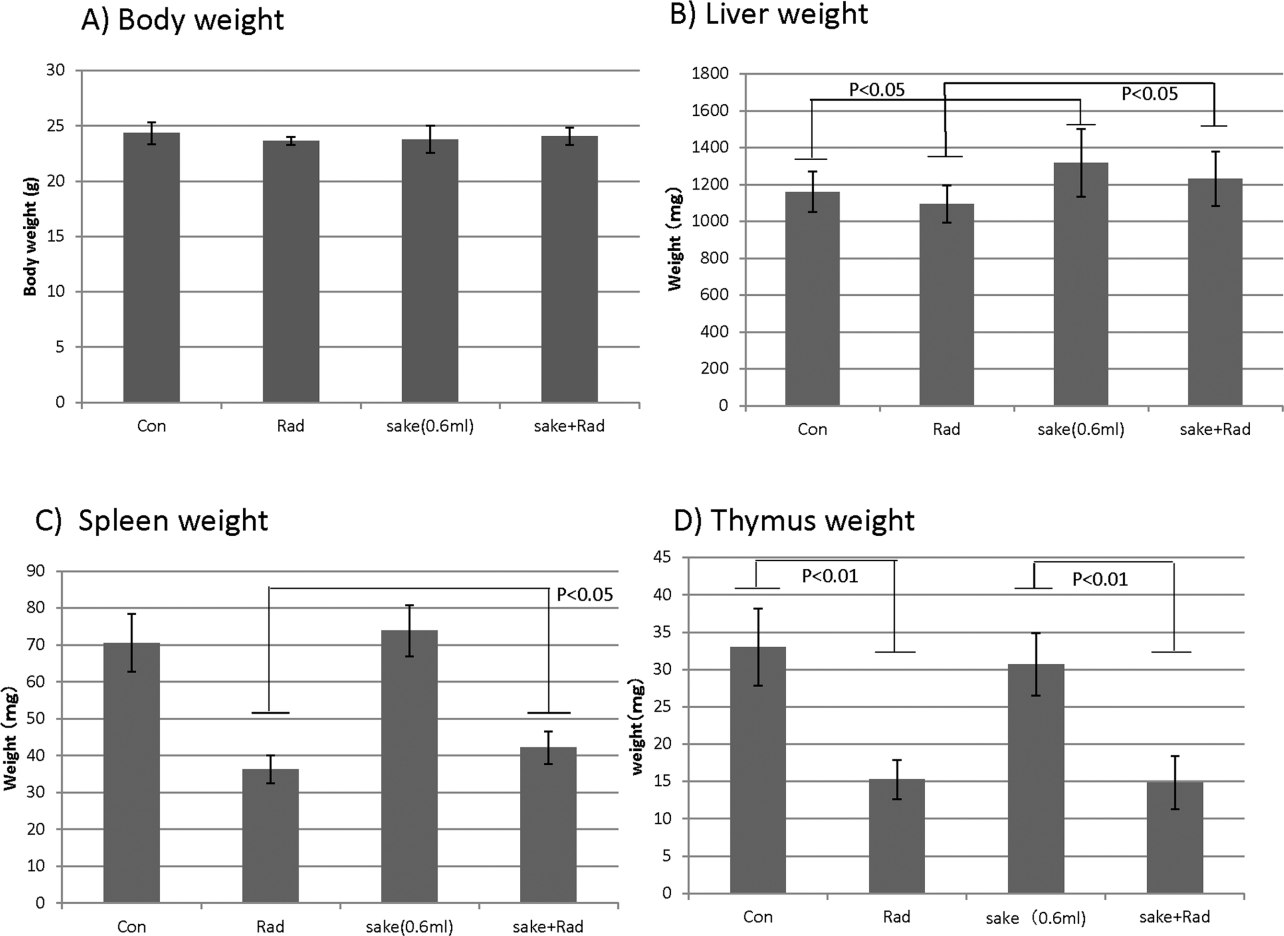
Effects of *sake* on the weight of the body, liver, spleen and thymus of irradiated mice. The weights of the (A) body, (B) liver, (C) spleen and (D) thymus were measured when blood and tissue samples were collected. Data are presented as means ± S.D. from seven mice in two independent experiments. Statistical analyses were performed by the unpaired t-test.

Mice administered pure Japanese *sake* (*junmai-shu*) or 15% (v/v) ethanol in water for one month appeared normal. During the one-month administration period, significant decreases in the body weights of mice in the groups administered ethanol or *sake* compared to the no intake group were observed; however, no significant difference in mean body weight was detectable at the end of the administration period compared to the control group. (S1 and S2 Figs and S1 and S2 Tables). Body and organ weights (liver, thymus, and spleen) of the mice administered with *sake* were also evaluated when blood and liver tissues were collected. No significant differences in body weights were observed between any of the treatment conditions (Fig 1A). However, liver weights increased slightly but significantly in mice administered *sake*, although radiation had no marked effects on liver weight (Fig 1B). The observed decreases in spleen weights in irradiated mice were slightly but significantly reversed by the administration of *sake* (Fig 1C). For the thymus, the marked decreases in weight induced by irradiation were similarly observed in irradiated mice administered *sake* (Fig 1D).

### Principal component analysis of metabolomic data

**Fig 2:**
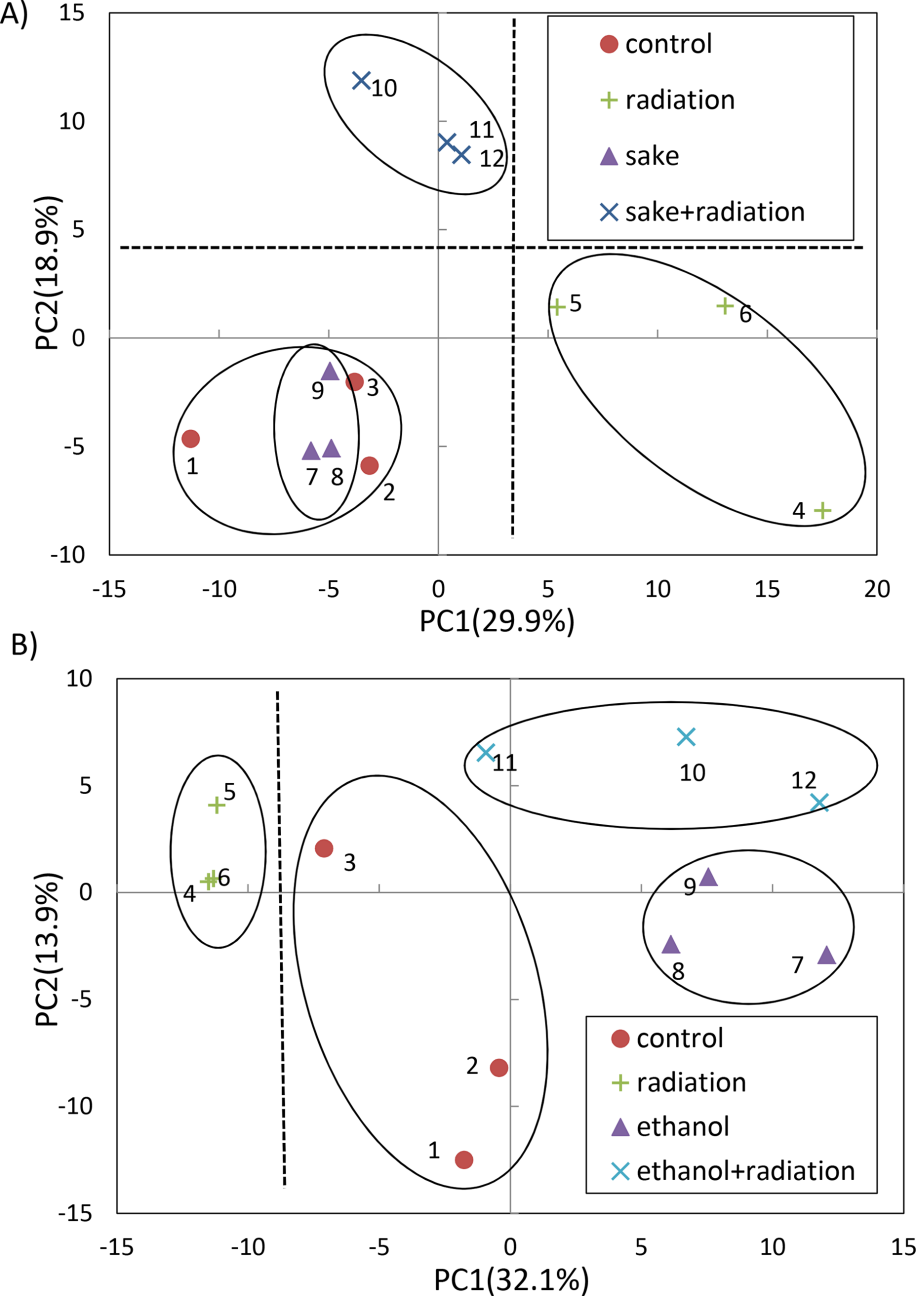
PCA of metabolic data for the combined effects of *sake* (or ethanol) and radiation on livers. (A) PCA in *sake* administration experiment, (B) PCA in 15% ethanol administration experiment. Percentage values indicated on the axes represent the contribution rate of the first (PC1) and second (PC2) principal components.

Metabolome analysis was next performed using CE-TOFMS to explore effects of *sake* on radiation-induced physiological alteration in the liver. In the analyses, a total of 230 (87 anions and 143 cations) and 245 metabolites (81 anions and 164 cations) were identified in the livers of mice administered *sake* and 15% ethanol, respectively (S3 and S4 Tables, respectively). PCA was performed to reveal differences in the metabolite profiles of the four treatment groups, which consisted of the controls, radiation, *sake* (or 15% ethanol) and the combination of both alcohol administration (*sake* or 15% ethanol) and radiation (Fig 2). In the PCA score plot, the group that received a combination of radiation and *sake* was clearly separated from the other three groups by the second principal component (PC2, 18.9% proportion; Fig 2A). The radiation and other groups appeared to be separated slightly along the first principal component (PC1, 29.9% proportion). The PCA score plot indicated that the metabolic profiles among individuals in each group were highly similar. A heat map representation of HCA (S3 Fig) also showed that clear differences were detectable among four groups and that individuals in each group have similar characteristics. In the case of 15% ethanol administration (Fig 2B), the group that received a combination of radiation and 15% ethanol was not clearly separated from the other groups along either PC1 or PC2. This lack of separation was in contrast to the group that received a combination of radiation and *sake* (Fig 2A). The PCA plot appears to show that 15% ethanol and radiation influence metabolic alterations of the liver in an independent manner, although the radiation and other three treatments groups were separated along PC1 (Fig 2B). Taken together, these results suggest that *sake* has distinct effects from ethanol on the influences of radiation on the liver metabolism.

### Effects of *sake* on the liver metabolome of irradiated mice

**Fig 3:**
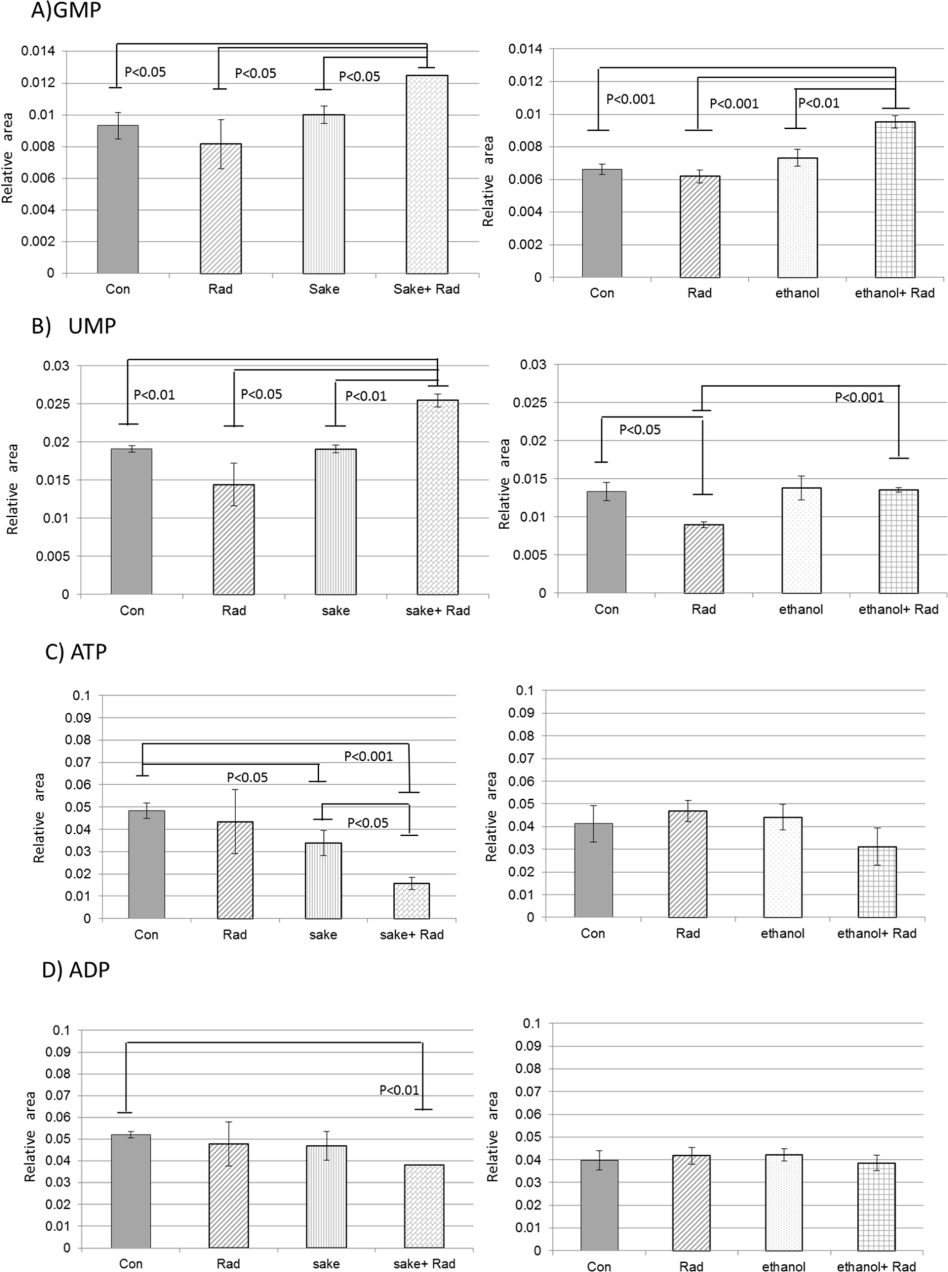
Effects of *sake* or ethanol on radiation-induced alterations of purine and pyrimidine metabolism in livers. (A) GMP, (B) UMP, (C) ATP, and (D) ADP. Data are relative area values of the metabolites and are presented as means ± S.D. of triplicate samples. Statistical analyses were performed by Welch’s *t*-test.

Using the correlation coefficients between the PC scores and variables for factor loading [22], we attempted to identify liver metabolites in irradiated mice were affected by *sake* administration. Metabolites that reached significant levels (p<0.01) in the evaluation of positive and negative correlation using the correlation coefficients were selected from the PC2 data generated from mice treated with a combination of radiation and *sake* (S5 Table). Among the selected metabolites, several were related to purine and pyrimidine metabolism and included AMP GMP, UMP, and CMP, which were found to be influenced by radiation. The relative area levels of GMP and UMP, which are representative nucleotide monophosphates, are shown in Fig 3A and 3B. Interestingly, although the diphosphates and triphosphates of adenosine (Fig 3C and 3D), guanosine, and uridine were not selected from PC2 data (S5 Table), the levels of these metabolites decreased in the livers of mice exposed to *sake* and radiation (S3 Table). Among the selected metabolites (S5 Table), seven metabolites (3-dephospho-CoA, GSH, nicotineamide, cysteine glutathione disulfide, GMP, UMP, and sedoheptulose 7-phosphate) were significantly modulated in the livers of mice treated with radiation and *sake* compared to the levels in the control, and *sake* and radiation alone-treated mice (S3 Table). In contrast, no changes in ATP, or ADP were detected in the livers of mice treated with the combination of radiation and ethanol (Fig 3C and 3D). Based on these findings for nucleotide metabolites, it appears that only guanosine nucleotide (GTP, GDP, and GMP) metabolism among nucleotides is influenced by radiation in the cases of both *sake* and ethanol. Although ATP depletion in combination of radiation and *sake* could result from hepatic failure induced by radiation or oxidative damages [23], taking it into consideration that a decrease in glutathione (GSH) is detected in liver diseases [24] but is not observed here as mentioned in the following part, it may be influenced by energy consumption for protection of livers or recovery from damages. In the livers of mice exposed to radiation and *sake*, the changes in several metabolites involved in glycolysis and pentose phosphate cycle, including glucose-6-phospate and sedoheptulose 7-phosphate, but not in the TCA cycle, were observed (S5 Table). ATP depletion may also be influenced by activities of these energy cycles. In addition, as nucleotide metabolism is known to be active in proliferating cells, including those of regenerating livers, the observed increases in several metabolites related to purine and pyrimidine metabolism such as AMP or GMP may be required for the repair and recovery of livers from damages [25–27]. Notably, as the increase in UMP was only observed in the liver of mice treated with a combination of *sake* and radiation, UMP might be involved in the metabolism of sugars present in sake [28].

**Fig 4:**
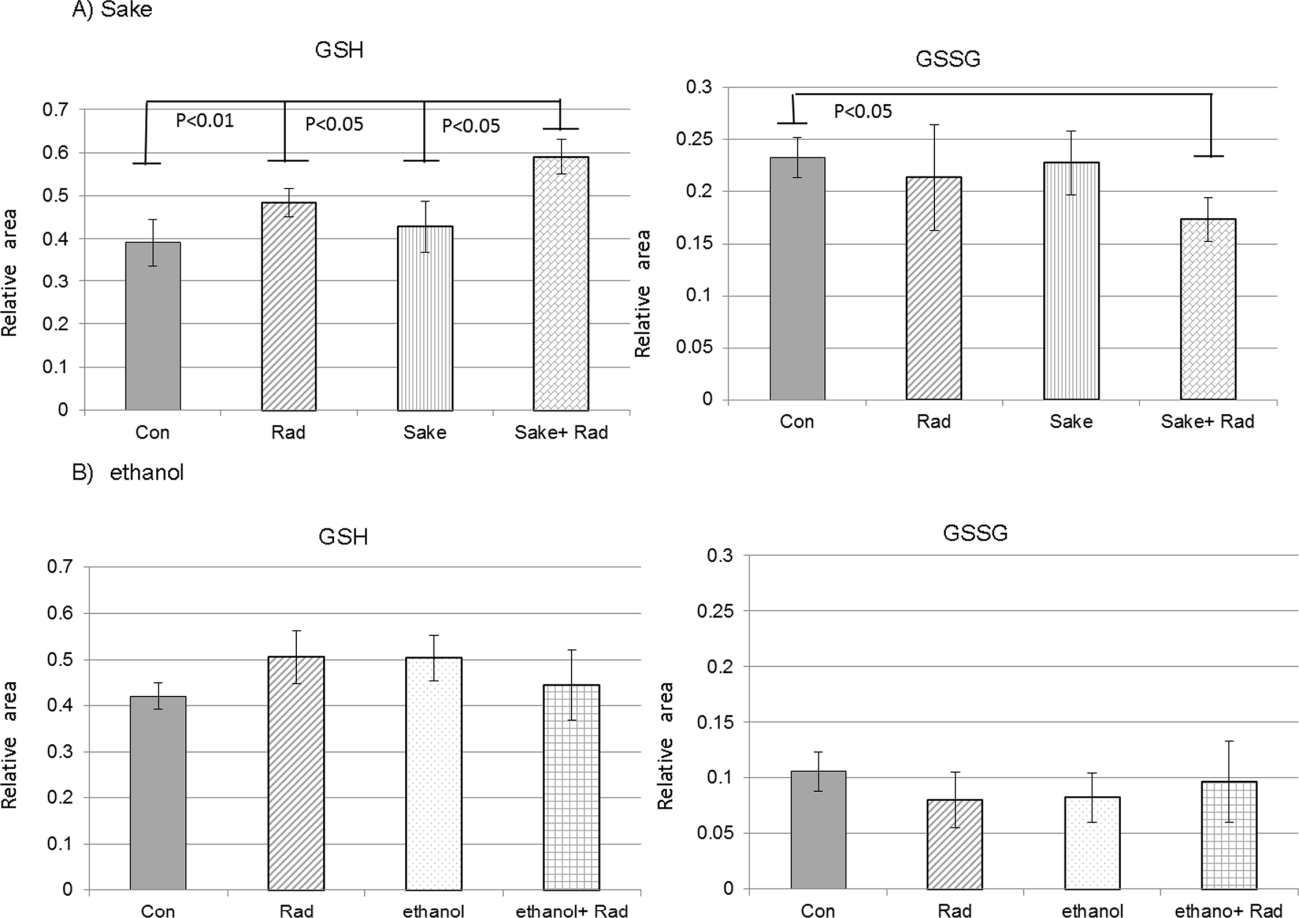
Effects of *sake* or ethanol on radiation-induced changes of GSH and GSSG in mouse livers. (A) *Sake* and (B) ethanol. Data are relative area values of the metabolites and are presented as means ± S.D. of triplicate samples. Statistical analyses were performed by Welch’s *t*-test.

Among the seven selected metabolites that were significantly modulated in the livers of mice treated with radiation and *sake*, GSH is an important regulator of redox homeostasis and GSH/GSSG (glutathione disulfide) is considered to be the major redox couple that determines anti-oxidative capacity. GSSG is the oxidized form of GSH, and the GSH/GSSG ratio is often used as an indicator of the cellular redox state. Here, the levels of GSH and GSSG significantly increased and decreased, respectively, in the livers of mice treated with a combination of radiation and *sake* (Fig 4). The changes in these metabolites were not observed in mice administered 15% ethanol instead of *sake* (Fig 4), suggesting that glutathione metabolism is specifically influenced by the consumption of *sake*.

### Characterization of radiation-induced metabolic alterations in livers

**Fig 5:**
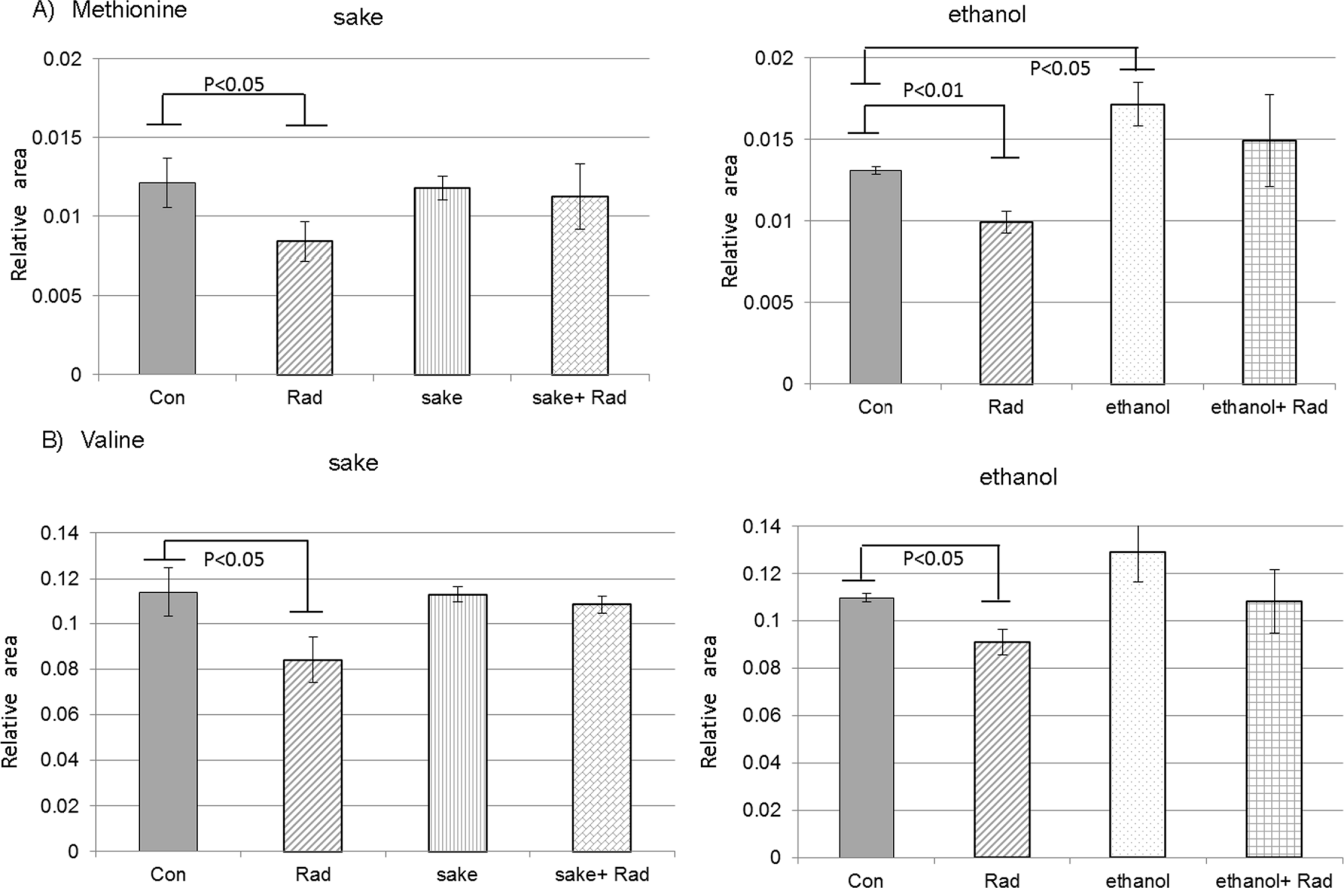
Changes in methionine and valine in the livers of irradiated mice administered sake or ethanol. (A) Methionine and (B) valine. Data are relative area values of the metabolites, and are presented as means ± S.D. of triplicate samples. Statistical analyses were performed using Welch’s *t*-test.

Metabolites that have significant correlation between the PC1 score and the relative peak area values were also selected in PC1, which characterized the radiation-alone group in the experiment examining *sake* and radiation (Fig 2A and S6 Table). The relative peak area values of 25 metabolites in the selected metabolites were significantly altered by radiation (S3 and S6 Tables). Among these metabolites, seven metabolites (3-methylhistidine, Val, Met, γ-butyrobetaine, N6-methyllysine, UDP-glucuronic acid, and UDP-glucose/UDP-galactose), which were also significantly changed on the relative peak area value levels in the livers of mice treated with radiation alone compared to the control in the experiment using 15% ethanol and radiation, were identified. These metabolites appeared to be altered stably and specifically in response to radiation in the livers of mice. With the exception of UDP-glucose/UDP-galactose, all of the metabolites decreased in response to radiation exposure alone. Radiation induced a decrease in the levels of methionine (Fig 5A), a finding that was previously reported [29], suggesting that alteration of methionine metabolism may be related to carcinogenesis [30,31]. The radiation-induced decrease in methionine levels was restored to control levels by the administration of *sake* or 15% ethanol. Although the administration of *sake* alone had no effect on the methionine level, *sake* administration diminished the effect of radiation on the levels of methionine. In the case of 15% ethanol, methionine levels induced by ethanol may have influenced the results (Fig 5A). It was reported that alcohol consumption impairs various methylation reactions in the liver [32]. However, a decrease in methionine in the livers of mice administered *sake* or ethanol alone was not observed. In contrast, the decrease in methionine induced by radiation was suppressed to control levels in irradiated mice after treatment with *sake* or ethanol. The contrasting results from these studies may be related to differences between mouse strains, sexes, or diets, with respect to the regulation of methylation reactions related to metabolism [33,34]. Further studies are therefore needed to determine the mechanisms underlying the mediation of methylation metabolism by *sake* or ethanol. Radiation was also shown to induce a decrease in the valine content of livers, which appeared to be influenced by the administration of *sake* or 15% ethanol (Fig 5B). Valine depletion is suggested to be associated with mTOR /S6K signaling suppression [35]. Interestingly, mTOR is related to reactive oxygen species (ROS) signaling [36]. Therefore, ROS induced by radiation might induce valine depletion, which in turn leads to the suppression of mTOR signaling. In addition, as supplementation of branched-chain amino acids, including valine, appears to reduce radiation-induced damage [37], the suppression of the valine depletion by exposure to *sake* or ethanol suggest that alcohol administration has protective abilities.

Here, metabolites that exhibited significant (P<0.01) trends using correlation coefficients between the PC scores and variables for factor loading in PC2 or PC1 as indicated in S5 and S6 Tables were compared. In addition, although the ethanol and *sake* administration were performed independently, metabolite levels in the control and radiation groups were measured in both experiments. As relative values were obtained in each experiment, they cannot be combined for the statistical analyses; however, similar changes in metabolite levels were detected in the control and radiation groups between two independent experiments. These results indicate that this experimental approach provides consistent results and allows changes in the selected metabolites to be evaluated.

### Alteration of metabolic biochemical markers in serum

**Fig 6:**
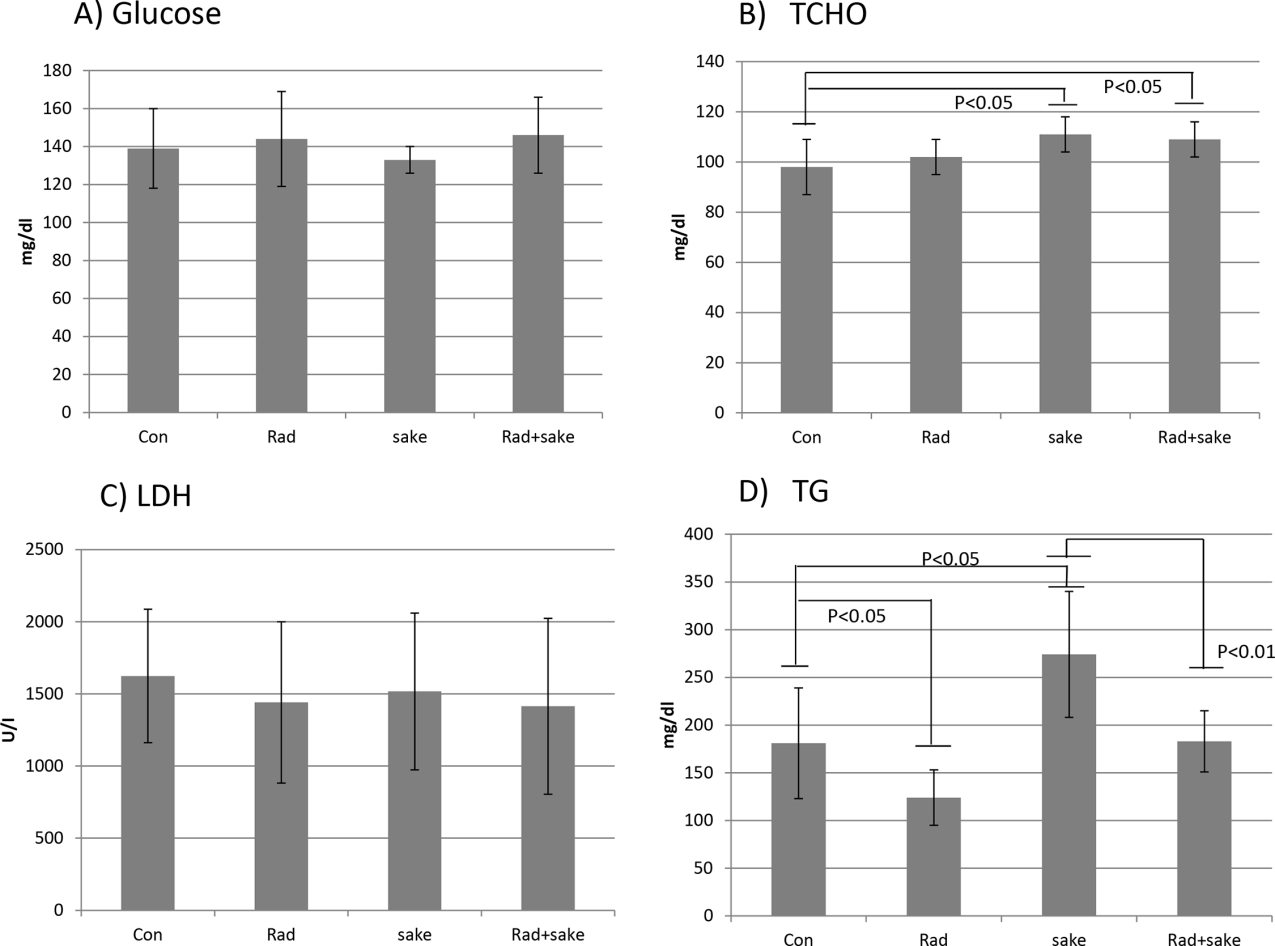
Effects of *sake* on metabolic biochemical markers in the serum of irradiated mice. (A) Glucose, (B) total cholesterol (TCHO), (C) lactose dehydrogenase (LDH), and (D) triglycerides (TG). Data are presented as means ± S.D. from seven mice in two independent experiments. Statistical analyses were performed by the unpaired *t*-test.

Changes in the serum levels of several metabolic biochemical markers that accompanied by alterations in liver metabolism in the four treatment groups were next evaluated. The serum levels of lactose dehydrogenase (LDH) and glucose were not markedly changed in mice treated with *sake* and/or radiation compared to the control group. Although the serum level of total cholesterol (TCHO) increased in mice administered *sake* alone, radiation had no effect on the level of TCHO; however, that of triglycerides (TG) increased in mice administered *sake* alone, and was reduced to control levels following irradiation (Fig 6). In this experiment, the amount of *sake* administered to mice seemed to be excessive because a significant increase in serum TG was observed compared to control mice. Although radiation alone induced a reduction in TG levels, the serum TG level in the treatment group that received both *sake* and radiation is greatly reduced to the control level from the level in mice administered *sake*. The observed reduction of TG by radiation in mice administered *sake* may be in part due to an induction of anti-oxidative responses, as indicated by the increase in GSH in the liver. The alcohol-induced accumulation of TG can reportedly be mitigated by a diet including foods that contain factors that promote anti-oxidative responses [38].

### Influence of *sake* administration on the effects of radiation

It has been reported that wine consumption mitigates the side effects associated with radio-cancer therapies [39] and that beer consumption can reduce the adverse effects of radiation [40]. However, a greater understanding of the effects of alcoholic beverages on responses to radiation is needed for assessing radiation risk or medical applications of alcohol. An omics-based approach was used to examine molecular changes in the livers of rats treated with *sake* [41]. However, such approaches have not been applied to the determination of the specific effects of *sake* on liver metabolism versus those of ethanol. To the best of our knowledge, metabolic analyses have not been performed from the viewpoint of mediation of a stress to other stress effects. *Sake* has anti-mutagenic effects, a property that has not been attributed to ethanol [14], and the administration of acute doses of *sake* has been shown to protect mice from the adverse effects of high-dose radiation more effectively than ethanol alone [15].

*Sake*, which is brewed from rice, water, and rice *koji* mold, contains numerous components, including sugars, amino acids, and vitamins [42]. GSH is induced by various components in foods, including rice proteins, and the effects we found seem to be due to the involved amino acid components in *sake* [43]. The induction of anti-oxidative activity is considered to be influenced by amino acids, including cysteine. The free amino acid composition of the *sake* used in this study was analyzed and was found to be comprised of relatively high levels of alanine, glutamate, and glycine, which are related to GSH synthesis (Table 1) [44]. Recently, it was demonstrated that glutamine and alanine induce anti-oxidative activity in livers [45]. In the present study, although the administration of *sake* alone had no marked effects on GSH regulation, and glutamine was not abundantly present in the *sake* (Table 1), the amino acid components of *sake* may be related to the altered regulation of GSH that was observed in irradiated mice after *sake* administration. The *sake* used in the present study and that evaluated in a report that demonstrated *sake* mitigates high-dose radiation effects [15] shared common characteristics, such as the abundance of alanine, glutamate, and glycine. Thus, it is possible that these amino acids are partly responsible for the beneficial effects of *sake* on radiation-induced damages.

**Table 1:** Free amino acids in *sake* (mg per 100g).

Amino acids	Amounts (mg) per 100g *sake*	Amino acids	Amounts (mg) per 100g *sake*
Asp	3	Cys	1
Thr	2	Met	<1
Ser	3	Ile	2
Asn	4	Leu	5
Glu	9	Tyr	5
Gln	<1	Phe	2
Pro	10(±1.2)	His	2
Gly	8	Lys	2
Ala	13(±1.2)	Trp	<1
Val	4	Arg	3

Data are presented as means (mg) of three samples from three different lots. Means (±SD) in the case of SD>1.

Although irradiation and *sake* administration were clearly shown to have interactive effects with respect to GSH regulation, the underlying mechanism remains unclear. We previously demonstrated that obesity mediates radiation sensitivity [8]. The harmful effects of radiation on living organisms, particularly in the case of low-linear energy transfer (LET) radiation, such as X-rays, are considered to result from radiation-induced oxidative stress. In the case of obesity, obesity-induced oxidative stress seems to mediate the effects of radiation in livers. *Sake* may influence redox homeostasis in the liver by a yet-unidentified mechanism, resulting in the alteration of GSH regulation following exposure to radiation.

Although the biological effects of alcohol administration have been investigated in various experimental models [32, 38, 46, 47], in the present study, we exposed mice to alcohol for one month as an experimental model. Chronic alcohol administration for 2 or 6 weeks has been used to evaluate the effects of alcohol on metabolism in mice [32, 38, 47], and consistent with these models, an increase in TG was also observed here, indicating that this model is considered as an appropriate model to evaluate chronic alcohol administration.

For the evaluation of factors in mechanisms related to liver diseases, it is necessary to identify the proteins and metabolites that are altered under various conditions [48]. Metabolome analysis provides insight into the underlying mechanisms that lead to the development of diseases, such as cancers, and may lead to the identification of potential therapeutic targets [49]. In the case of alcohol-related disease, a relationship between chronic alcohol consumption and fatty liver has been confirmed [2]. In addition, the development of fatty liver appears to be influenced by radiation [50]. The present findings that *sake* consumption mitigates the effects of radiation may provide insight into relationship between alcohol-related diseases and radiation effects.

As an experimental model, we used mice administered either *sake* or ethanol that were then exposed to fractionated irradiation (0.75 Gy, 4 times). Fractionated irradiation with approximately 0.75 Gy has been performed at various intervals in several studies [51–53]. The dose per fraction used here was similar to the lowest value used in a report evaluating the combined effects of radiation and chemical treatment on carcinogenesis [51]. Compared to single irradiation, fractionated irradiation increases the potential that radiation-related effects that are altered in response to chronic *sake* administration can be detected. In the present model, to evaluate effects of radiation on mice exposed to chronic alcohol intake, the fractionated irradiation was performed during the last week of the intake period. In addition, because the fractionated dose has been used in experimental models for evaluating the effects of radiation on normal cells or carcinomas in radiation therapies [52, 53], the results from the present study may contribute to medical applications. Though the development of second primary cancers after radiation therapy may be influenced by lifestyles, there are limited data for the influence of specific factors, with the exception of tobacco, on their epidemiology [54]. For this reason, the National Council on Radiation Protection & Measurements (NCRP) has recommended that the relationships between second primary cancers and lifestyles should be investigated [55,56].

Alcohol consumption represents a type of caloric intake and considered to influence body weight, although this relationship is controversial [57, 58]. Although the final body weights of the mice administered ethanol or *sake* did not significantly differ from that of the control group (S1 and S2 Tables), fluctuations in body weights were observed during the administration period. In particular, clear decreases in mice administered 15% ethanol or *sake* were detected approximately two to three weeks after the start of the administration period. As decreases in food intake in mice administered 15% ethanol were observed during this period (S7 Table), the observed decreases in body weight were likely related to decreased food intake. Although the reduction in food intake appeared to have recovered at the end of the administration period, the observed changes in metabolites or TG may have resulted from alterations in calorie consumption after alcohol administration.

Drinking *sake* in moderation has beneficial and protective health effects, and may provide partial protection against accidental or medical radiation exposure. Although the amount of *sake* administration in this study is not small, the level appears to induce protective effects and is practical for evaluation in mouse experimental model [15]. If the natural components in *sake* that promote these protective effects, are identified, they may contribute to the understanding of the benefits of certain foods on health and potentially be used in the clinical setting. To this end, evaluation of effects of single irradiation on metabolisms or identification of metabolic markers by lipidomics would be needed. The findings presented here warrant the further study of the beneficial components present in *sake* and analysis of the metabolic networks altered by *sake* consumption.

## Conclusions

Chronic Japanese *sake* consumption induces specific metabolic alterations in the liver in response to irradiation. Although excess *sake* consumption may induce adverse effects on the liver, *sake* intake has the potential to promote anti-oxidative stress activities following radiation exposure. The findings presented here suggest that moderate *sake* consumption may promote anti-oxidative activity following exposure to stress such as radiation, thereby limiting the adverse effects typically associated with these stresses.

## Supporting Information

S1 Fig Effects of *sake* administration on the body weight of mice (n = 4). (PDF)

S2 Fig Effects of 15% ethanol administration on the body weight of mice (n = 5). (PDF)

S3 Fig Heat map for metabolic analysis by HCA in the *sake* administration experiment. The X-axis is labeled with group names and sample numbers and the Y-axis shows peaks. Sample numbers on the X-axis correspond to the numbers in Fig 2A. Peaks were analyzed by HCA and distances are depicted by a tree diagram. In the color legend, the lowest value in the map is represented by a bright green, and the highest value is represented by a bright red.
(PDF)

S1 Table Changes in weights during the administration period of *sake* (0.6ml). (PDF)

S2 Table Changes in weights during the administration period of 15% ethanol. (PDF)

S3 Table Metabolome data for the livers of irradiated mice administered *sake*. Values represent the relative areas of each metabolite peak to the peak area of an internal standard.
(PDF)

S4 Table Metabolome data for the livers of irradiated mice administered 15% ethanol. Values represent the relative areas of each metabolite peak to the peak area of an internal standard.
(PDF)

S5 Table Selected metabolites. These have significant correlation (P<0.01) based on factor loadings between the PC2 scores and variables in the metabolome data for irradiated mice administered sake.
(PDF)

S6 Table Selected metabolites. These have significant correlation (P<0.01) based on factor loadings between the PC1 scores and variables in the metabolome data for irradiated mice administered *sake*.
(PDF)

S7 Table Changes in food intake during the administration of 15% ethanol. (PDF)
