# Supplementary figures and images for: Chronic Intake of Japanese Sake Mediates Radiation-Induced Metabolic Alterations in Mouse Liver
Source: PLoS One. 2016 Jan 11;11(1):e0146730. doi: 10.1371/journal.pone.0146730 (PMC4713437; doi:10.1371/journal.pone.0146730)

S1 Fig. Effects of sake administration on the body weight of mice (n=4).

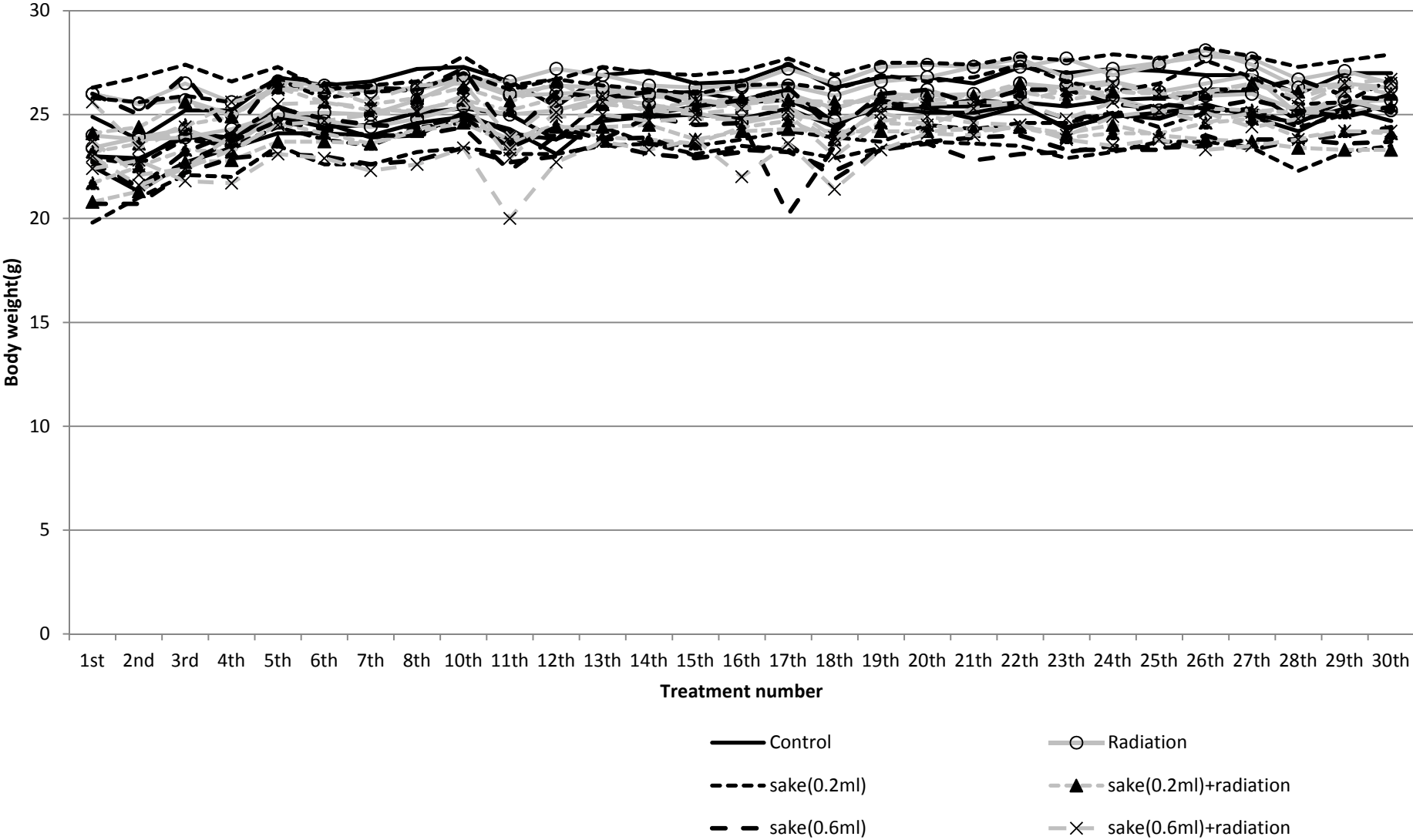

Supplement: S1 Fig — (PDF) [file pone.0146730.s001.pdf]

S2 Fig. **Effects of 15% ethanol administration on the body weight of mice (n=5).**

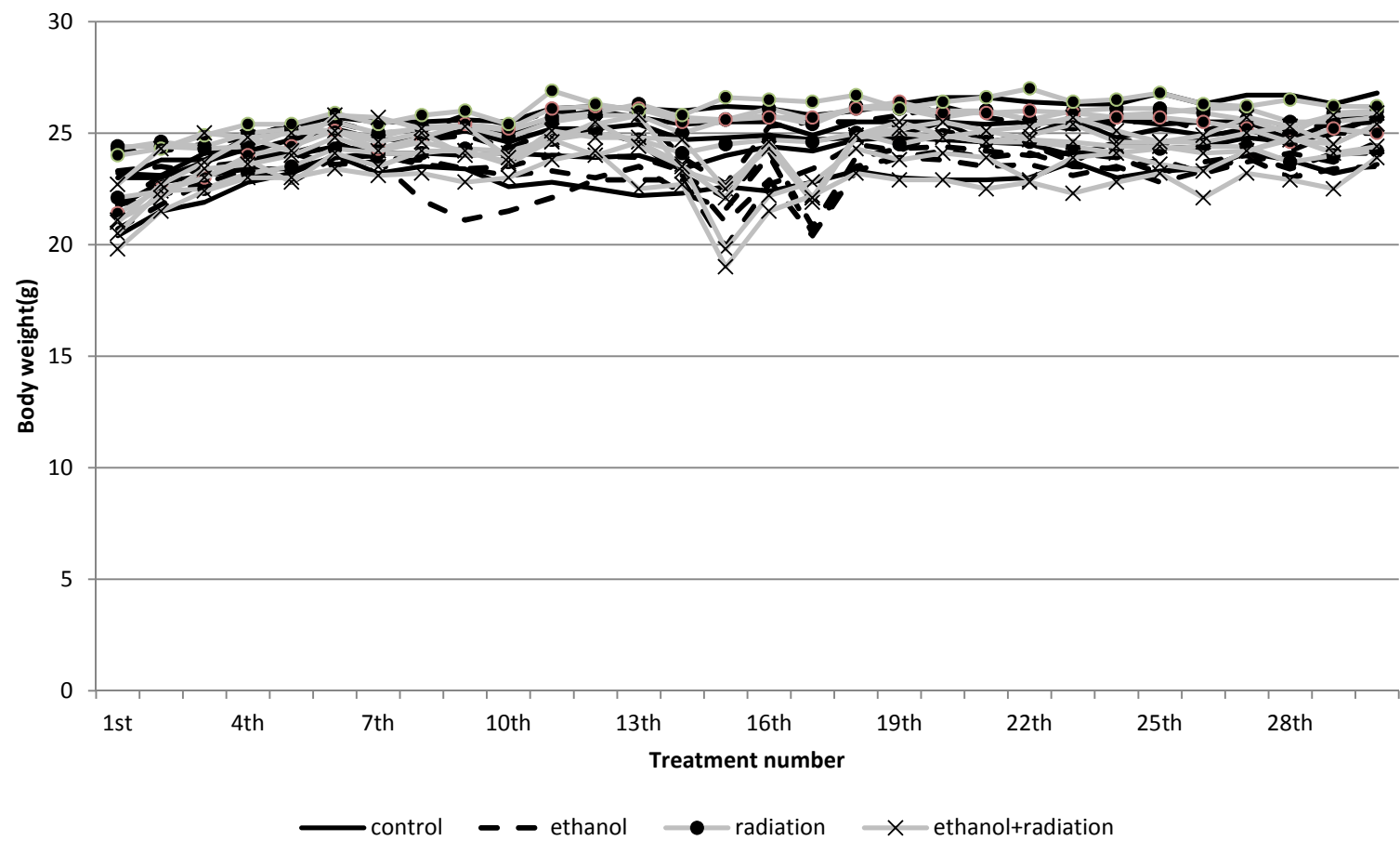

Supplement: S2 Fig — (PDF) [file pone.0146730.s002.pdf]

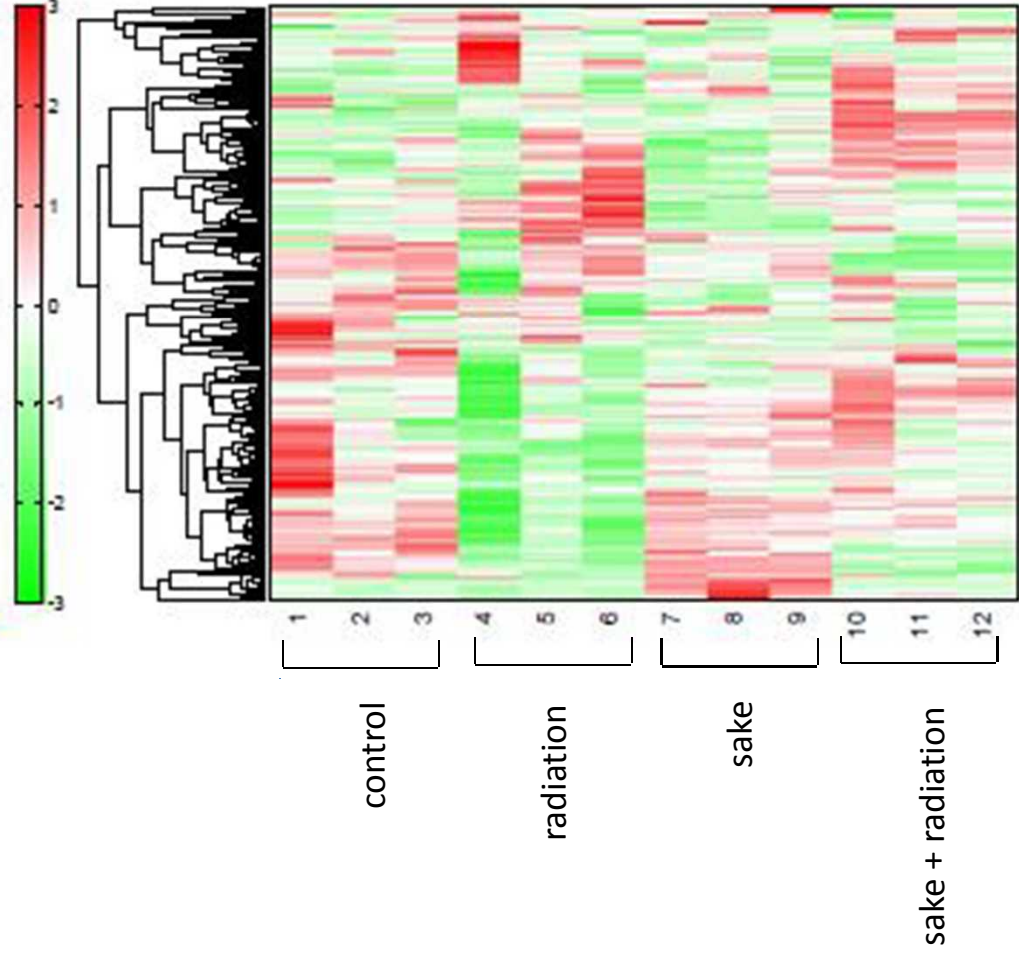

Supplement: S3 Fig — The X-axis is labeled with group names and sample numbers and the Y-axis shows peaks. Sample numbers on the X-axis correspond to the numbers in Fig 2A. Peaks were analyzed by HCA and distances are depicted by a tree diagram. In the color legend, the lowest value in the map is represented by a bright green, and the highest value is represented by a bright red. (PDF) [file pone.0146730.s003.pdf]
