# Supplementary material for: Chronic Intake of Japanese Sake Mediates Radiation-Induced Metabolic Alterations in Mouse Liver
Source: PLoS One. 2016 Jan 11;11(1):e0146730. doi: 10.1371/journal.pone.0146730 (PMC4713437; doi:10.1371/journal.pone.0146730)
Supplement: S1 Table — (PDF) [file pone.0146730.s004.pdf]

S1 Table. Mean body weight of the treatment groups throughout the administration period.

Administration Day	Weight of control group	Weight of radiation group	Weight in sake group	Weight in sake+radiation group	Statistic analysis, no intake (control+radiation) groups vs sake intake groups (N=8,before irradiation)	Statistic analysis (N=4, after irradiation)
1	23.35±1.1	24.85±1.4	23.05±2.2	23.68±1.4	NS	
2	22.70±1.0	24.69±0.97	22.18±1.9	22.48±0.79	NS	
3	23.98±1.0	25.10±1.2	23.75±2.1	22.75±1.1	NS	
4	24.08±0.78	24.60±0.91	23.68±0.54	23.65±1.6	NS	
5	25.30±1.1	25.73±0.85	25.00±1.6	24.83±1.3	NS	
6	24.88±1.0	25.60±0.83	24.65±1.4	24.80±1.4	NS	
7	24.75±1.3	25.45±0.9	24.35±1.5	24.58±1.5	NS	
8	25.23±1.4	25.75±0.69	24.35±1.4	24.83±1.6	NS	
9	ND	ND	ND	ND		
10	25.65±1.1	26.43±0.7	24.98±1.6	25.38±1.4	NS	
11	25.00±1.1	25.88±0.66	23.08±0.71	22.53±1.7	P<0.01	
12	24.03±0.97	26.20±0.83	24.38±1.3	24.58±1.3	NS	
13	25.55±1.0	26.28±0.46	24.25±1.0	25.05±0.97	P<0.05	
14	25.75±1.0	25.95±0.37	24.50±1.3	24.58±0.88	P<0.05	
15	25.75±0.68	25.75±0.5	23.95±1.2	24.83±0.78	P<0.01	
16	25.68±0.74	25.70±0.84	24.20±1.2	24.38±1.6	P<0.05	
17	26.15±0.91	26.15±0.91	23.33±2.5	24.80±0.82	P<0.05	
18	25.00±0.88	25.60±0.77	22.98±1.1	22.98±1.1	P<0.01	
19	25.80±0.69	26.33±0.81	24.55±1.5	24.63±0.93	P<0.01	
20	25.65±0.78	26.58±0.67	24.70±1.3	24.75±0.6	P<0.01	
21	25.45±0.74	26.53±0.92	24.48±1.4	24.88±0.72	P<0.05	
22	25.95±0.9	26.88±0.77	24.70±1.4	25.05±0.64	P<0.01	
23	25.28±1.3	26.80±0.64	24.38±1.4	24.70±1.1	P<0.05	
24	25.70±1.1	26.63±0.6	24.33±1.1	25.13±1.3	P<0.05	
25	25.70±1.0	26.55±1.1	24.38±1.2	25.05±0.91	P<0.01	
26	25.98±0.69	27.08±1.0	24.75±1.2	24.78±1.2	P<0.01	
27	25.73±0.95	27.00±0.74	24.75±1.4	24.88±1.3	P<0.05	
28	24.88±0.78	25.80±0.83	24.88±1.4	24.95±0.87		NS to control
29	25.83±0.81	26.23±0.71	24.60±1.0	25.83±1.1		NS to control
30	25.70±1.0	25.93±0.59	24.8±0.92	25.88±1.1		NS to control

Data are presented as the mean (g) ± SD
Body weight on administration day 9 was not not determined (ND). and the sake administration was therefore performed based on the 8th weight.
Statiistic analysis was performed by the two-tailed unpaired t-test.
NS, not significant.
