# Supplementary material for: Chronic Intake of Japanese Sake Mediates Radiation-Induced Metabolic Alterations in Mouse Liver
Source: PLoS One. 2016 Jan 11;11(1):e0146730. doi: 10.1371/journal.pone.0146730 (PMC4713437; doi:10.1371/journal.pone.0146730)
Supplement: S2 Table — (PDF) [file pone.0146730.s005.pdf]

S2 Table. Mean body weight of the treatment groups throughout the administration period.

Administration day	Weight in control group	Weight of radiation group	Weight of ethanol group	Weight of ethanol+radiation group	Statistic analysis, no intake (control+radiation groups) groups vs ethanol intake groups (N=10, before irradiation)	Statistic analysis (N=5, after irradiation)
1	22.26±1.2	23.24±1.4	22.34±1.4	21.02±1.1	NS	
2	22.84±0.84	23.64±1.1	22.96±0.97	22.60±1.0	NS	
3	23.32±0.87	23.82±1.0	23.36±0.7	23.50±0.97	NS	
4	23.90±0.79	24.40±0.86	23.84±0.57	24.00±0.79	NS	
5	24.14±0.92	24.72±0.78	24.12±0.92	23.80 ±0.91	NS	
6	24.64± 0.78	25.18 ±0.59	24.22± 0.83	24.64± 0.92	NS	
7	24.34±0.84	24.72 ±0.53	24.16 ±0.52	24.22± 1.0	NS	
8	24.52 ±0.76	25.04 ±0.63	23.84 ±1.2	24.44 ±0.79	NS	
9	24.64 ±0.91	25.28 ±0.61	23.72± 1.8	24.10± 0.89	P<0.05	
10	24.36± 1.1	24.94± 0.47	23.56 ±1.4	23.66± 0.42	P<0.05	
11	24.76 ±1.4	25.78 ±0.81	24.10± 1.5	24.60 ±0.48	P<0.05	
12	24.76± 1.6	25.84± 0.51	24.20±1.3	24.78 ±0.68	NS	
13	24.68± 1.6	25.78± 0.63	24.16 ±1.2	24.42 ±1.2	P<0.05	
14	24.36 ±1.5	25.22 ±0.70	23.10±0.58	23.56 ±0.73	P<0.01	
15	24.62 ±1.4	25.58 ±0.74	21.46± 1.1	21.22 ±1.7	P<0.01	
16	24.66 ±1.4	25.76 ±0.67	23.80±1.12	23.44± 1.5	P<0.01	
17	24.48± 1.1	25.54± 0.65	22.12 ±2.3	22.22 ±0.34	P<0.01	
18	24.86 ±1.0	26.02±0.62	24.14 ±0.89	24.42 ±0.73	P<0.01	
19	24.90 ±1.2	25.84 ±0.76	24.42 ±0.79	24.48± 1.1	P<0.05	
20	25.04± 1.4	25.78 ±0.55	24.74± 0.94	24.44 ±0.99	P<0.05	
21	24.82± 1.3	25.82 ±0.74	24.40±0.85	24.24± 1.1	P<0.05	
22	24.86 ±1.3	25.76 ±0.86	24.32 ±0.68	24.12 ±1.2	P<0.05	
23	25.00 ±1.1	25.64 ±0.76	23.92 ±0.8	24.38± 1.4	P<0.05	
24	24.86 ±1.2	25.64± 0.92	24.18 ±0.85	24.20 ±0.86	P<0.05	
25	25.00 ±1.3	25.76 ±0.92	24.00±1.1	24.08 ±0.64	P<0.01	
26	24.90 ±1.1	25.64± 0.75	24.02 ±0.88	23.80 ±1.1	P<0.01	
27	25.24 ±0.96	25.50 ±0.73	24.48 ±0.86	24.46 ±0.94	P<0.05	
28	24.92± 1.1	25.06± 1.0	23.98 ±0.84	24.6± 0.99		NS (each group to Control)
29	25.02± 1.1	25.36± 0.85	24.32 ±0.85	24.28 ±1.2		NS (each group to Control)
30	25.56± 1.2	25.42 ±0.82	24.54± 0.78	25.00±0.82		NS (each group to Control)

Data are presented as mean (g) ± SD

Statiistic analysis was performed by the two-tailed unpaired t-test.

NS, not significant.
