# Supplementary material for: Chronic Intake of Japanese Sake Mediates Radiation-Induced Metabolic Alterations in Mouse Liver
Source: PLoS One. 2016 Jan 11;11(1):e0146730. doi: 10.1371/journal.pone.0146730 (PMC4713437; doi:10.1371/journal.pone.0146730)
Supplement: S3 Table — Values represent the relative areas of each metabolite peak to the peak area of an internal standard. (PDF) [file pone.0146730.s006.pdf]

S3 Table. Metabolome data for the livers of irradiated mice administered sake. Values represent the relative areas of each metabolite peak to the peak area of an internal standard.

Relative Area

Compound name	control			rad			sake			sake+rad			control		rad		sake		sake+rad	
	1	2	3	4	5	6	7	8	9	10	11	12	Mean	S.D.	Mean	S.D.	Mean	S.D.	Mean	S.D.
Octanoic acid	N.D.	N.D.	N.D.	N.D.	N.D.	1.3E-04	3.8E-04	5.4E-04	4.2E-04	N.D.	N.D.	N.D.	N.D.	N.A.	1.3E-04	N.A.	4.4E-04	8.7E-05	N.D.	N.A.
XA0013	N.D.	N.D.	N.D.	1.7E-04	N.D.	N.D.	N.D.	N.D.	4.6E-04	N.D.	N.D.	N.D.	N.D.	N.A.	1.7E-04	N.A.	4.6E-04	N.A.	N.D.	N.A.
Fructose 1,6-diphosphate	2.9E-04	3.6E-04	3.1E-04	3.2E-03	4.9E-04	3.8E-04	3.1E-04	2.8E-04	2.8E-04	2.7E-04	3.7E-04	3.8E-04	3.2E-04	3.8E-05	1.4E-03	1.6E-03	2.9E-04	1.8E-05	3.4E-04	6.2E-05
XA0055	2.5E-04	2.4E-04	4.7E-04	2.0E-03	6.2E-04	3.9E-04	N.D.	3.0E-04	2.4E-04	1.6E-04	8.5E-04	6.0E-04	3.2E-04	1.3E-04	9.9E-04	8.5E-04	2.7E-04	4.2E-05	5.4E-04	3.5E-04
UDP-glucose UDP-galactose	7.8E-03	1.1E-02	1.4E-02	2.4E-02	2.9E-02	3.6E-02	9.5E-03	8.9E-03	1.2E-02	1.9E-02	1.6E-02	1.8E-02	1.1E-02	3.2E-03	3.0E-02	6.1E-03	1.0E-02	1.6E-03	1.8E-02	1.5E-03
Taurocholic acid	4.6E-02	4.1E-02	3.3E-02	1.2E-01	4.9E-02	8.3E-02	5.0E-02	7.5E-02	2.3E-02	4.7E-02	1.8E-02	3.0E-02	4.0E-02	6.6E-03	8.3E-02	3.4E-02	4.9E-02	2.6E-02	3.2E-02	1.4E-02
3',5'-ADP	2.3E-04	2.5E-04	2.6E-04	3.9E-04	3.5E-04	6.7E-04	2.3E-04	2.3E-04	2.6E-04	1.9E-04	1.8E-04	1.5E-04	2.5E-04	1.6E-05	4.7E-04	1.7E-04	2.4E-04	1.8E-05	1.7E-04	2.2E-05
Dihydroxyacetone phosphate	2.1E-03	2.4E-03	3.8E-03	9.4E-03	4.2E-03	2.1E-03	1.7E-03	2.6E-03	2.4E-03	1.2E-03	4.7E-03	2.8E-03	2.8E-03	8.8E-04	5.2E-03	3.8E-03	2.2E-03	4.6E-04	2.9E-03	1.7E-03
S-Lactoylglutathion	2.0E-04	1.4E-04	1.7E-04	2.3E-04	1.8E-04	4.8E-04	2.2E-04	2.6E-04	7.2E-04	1.6E-04	4.1E-04	2.2E-04	1.7E-04	2.8E-05	3.0E-04	1.6E-04	4.0E-04	2.7E-04	2.6E-04	1.3E-04
Glycerophosphocholine	4.9E-02	5.6E-02	8.2E-02	7.5E-02	1.1E-01	1.3E-01	5.2E-02	6.6E-02	7.9E-02	9.5E-02	9.3E-02	1.1E-01	6.3E-02	1.8E-02	1.1E-01	3.0E-02	6.6E-02	1.3E-02	9.8E-02	6.4E-03
5,6,7,8-Tetrahydrobiopterin	N.D.	N.D.	1.2E-04	N.D.	N.D.	1.9E-04	N.D.	N.D.	1.1E-04	1.4E-04	1.4E-04	1.2E-04	1.2E-04	N.A.	1.9E-04	N.A.	1.1E-04	N.A.	1.3E-04	9.8E-06
Triethanolamine	6.0E-05	7.4E-05	6.4E-05	1.4E-04	1.1E-04	6.7E-05	8.5E-04	6.4E-05	7.4E-05	6.1E-05	5.6E-05	9.0E-05	6.6E-05	7.5E-06	1.1E-04	3.7E-05	3.3E-04	4.5E-04	6.9E-05	1.8E-05
γ-Glu-2-aminobutyric	1.5E-04	1.5E-04	2.3E-04	2.3E-04	3.2E-04	3.1E-04	1.8E-04	1.6E-04	1.5E-04	1.7E-04	1.9E-04	2.0E-04	1.8E-04	4.7E-05	2.9E-04	5.0E-05	1.6E-04	1.2E-05	1.9E-04	1.4E-05
Cys	1.6E-04	1.3E-04	1.4E-04	2.0E-04	2.2E-04	2.6E-04	1.5E-04	2.4E-04	2.3E-04	4.2E-04	4.5E-04	4.0E-04	1.4E-04	1.8E-05	2.3E-04	2.7E-05	2.1E-04	4.7E-05	4.2E-04	2.9E-05
Inosine	2.0E-02	2.0E-02	2.5E-02	4.2E-02	3.1E-02	2.9E-02	2.3E-02	2.6E-02	3.2E-02	4.1E-02	3.3E-02	3.3E-02	2.1E-02	2.7E-03	3.4E-02	6.8E-03	2.7E-02	4.5E-03	3.6E-02	4.6E-03
Phosphoenolpyruvic acid	4.6E-04	5.4E-04	6.8E-04	8.5E-04	8.7E-04	9.0E-04	3.8E-04	4.5E-04	4.0E-04	5.7E-04	5.2E-04	7.5E-04	5.6E-04	1.1E-04	8.7E-04	2.7E-05	4.1E-04	3.7E-05	6.1E-04	1.2E-04
Creatine	3.2E-02	3.0E-02	2.8E-02	4.4E-02	4.9E-02	4.6E-02	2.5E-02	2.5E-02	2.6E-02	5.0E-02	3.6E-02	3.5E-02	3.0E-02	1.9E-03	4.6E-02	2.7E-03	2.5E-02	4.4E-04	4.0E-02	8.3E-03
3-Phosphoglyceric	1.0E-03	1.2E-03	1.4E-03	1.8E-03	1.7E-03	2.1E-03	6.9E-04	9.2E-04	8.7E-04	1.2E-03	1.2E-03	1.6E-03	1.2E-03	2.0E-04	1.9E-03	2.2E-04	8.3E-04	1.2E-04	1.3E-03	2.1E-04
XA0035	4.3E-04	4.3E-04	4.4E-04	5.7E-04	6.3E-04	7.5E-04	3.1E-04	4.1E-04	4.3E-04	5.4E-04	5.1E-04	4.2E-04	4.3E-04	7.0E-06	6.5E-04	9.1E-05	3.8E-04	6.0E-05	4.9E-04	6.0E-05
Homovanillic	1.6E-04	2.4E-04	2.0E-04	1.5E-04	4.2E-04	3.1E-04	2.4E-04	2.0E-04	2.0E-04	3.2E-04	1.9E-04	3.0E-04	2.0E-04	4.0E-05	2.9E-04	1.4E-04	2.1E-04	2.2E-05	2.7E-04	6.7E-05
Ascorbic acid	1.6E-02	2.0E-02	2.3E-02	2.0E-02	2.8E-02	3.6E-02	1.5E-02	1.7E-02	2.7E-02	3.6E-02	3.1E-02	2.8E-02	1.9E-02	3.7E-03	2.8E-02	7.5E-03	2.0E-02	6.6E-03	3.2E-02	3.8E-03
3'-Dephospho	1.8E-04	1.2E-04	1.7E-04	2.0E-04	2.0E-04	2.8E-04	1.6E-04	2.0E-04	2.7E-04	4.8E-04	3.8E-04	4.8E-04	1.6E-04	3.3E-05	2.2E-04	4.8E-05	2.1E-04	5.7E-05	4.4E-04	5.7E-05
N-Acetylglucosami	3.8E-04	4.4E-04	5.5E-04	5.4E-04	5.1E-04	8.7E-04	4.2E-04	4.2E-04	4.0E-04	5.8E-04	4.9E-04	5.3E-04	4.6E-04	8.6E-05	6.4E-04	2.0E-04	4.1E-04	8.4E-06	5.3E-04	4.7E-05
2,3-Diphosphoglyceric acid	5.6E-03	7.9E-03	4.9E-03	1.3E-02	6.7E-03	5.9E-03	6.2E-03	5.9E-03	4.2E-03	5.1E-03	5.6E-03	5.9E-03	6.1E-03	1.6E-03	8.5E-03	3.9E-03	5.4E-03	1.1E-03	5.5E-03	4.0E-04
5-Oxoproline	6.3E-04	4.4E-04	4.9E-04	9.8E-04	5.8E-04	6.0E-04	7.7E-04	5.7E-04	5.5E-04	8.7E-04	7.7E-04	7.5E-04	5.2E-04	9.8E-05	7.2E-04	2.3E-04	6.3E-04	1.2E-04	8.0E-04	6.5E-05
Ribulose 5-phosphate	6.0E-03	5.8E-03	6.2E-03	9.4E-03	6.8E-03	8.2E-03	5.9E-03	6.5E-03	6.2E-03	8.8E-03	7.6E-03	7.8E-03	6.0E-03	2.0E-04	8.1E-03	1.3E-03	6.2E-03	3.1E-04	8.1E-03	6.8E-04
Fructose 6-phosphate	6.7E-03	4.9E-03	6.9E-03	1.2E-02	6.3E-03	6.7E-03	6.5E-03	7.2E-03	6.9E-03	2.6E-03	6.0E-03	4.2E-03	6.2E-03	1.1E-03	8.4E-03	3.1E-03	6.8E-03	3.5E-04	4.3E-03	1.7E-03
Adenosine	2.8E-02	3.0E-02	2.9E-02	2.7E-02	4.2E-02	4.7E-02	2.7E-02	2.4E-02	1.7E-02	3.5E-02	2.5E-02	2.9E-02	2.9E-02	8.8E-04	3.9E-02	1.1E-02	2.2E-02	5.1E-03	2.9E-02	5.1E-03
XC0126	1.8E-04	1.4E-04	1.4E-04	1.7E-04	1.7E-04	2.6E-04	1.4E-04	1.5E-04	1.7E-04	2.3E-04	2.0E-04	1.7E-04	1.6E-04	2.3E-05	2.0E-04	5.0E-05	1.5E-04	1.8E-05	2.0E-04	3.0E-05
Gln	2.5E-01	2.3E-01	2.2E-01	3.5E-01	2.6E-01	2.8E-01	2.1E-01	2.4E-01	2.1E-01	2.7E-01	2.6E-01	2.6E-01	2.3E-01	1.7E-02	3.0E-01	4.4E-02	2.2E-01	1.7E-02	2.6E-01	4.9E-03
Hexanoic acid	2.4E-04	N.D.	2.4E-04	N.D.	3.0E-04	N.D.	4.6E-04	9.1E-04	5.6E-04	3.0E-04	2.5E-04	N.D.	2.4E-04	4.3E-06	3.0E-04	N.A.	6.4E-04	2.4E-04	2.8E-04	3.6E-05
Hypoxanthine	3.4E-03	4.2E-03	4.2E-03	7.4E-03	4.9E-03	2.4E-03	5.5E-03	5.8E-03	5.1E-03	9.0E-03	6.9E-03	7.2E-03	3.9E-03	4.6E-04	4.9E-03	2.5E-03	5.5E-03	3.9E-04	7.7E-03	1.1E-03
N-	6.1E-04	5.4E-04	6.5E-04	8.0E-04	6.7E-04	7.7E-04	5.8E-04	6.4E-04	4.9E-04	7.2E-04	5.8E-04	6.2E-04	6.0E-04	5.5E-05	7.5E-04	6.7E-05	5.7E-04	7.7E-05	6.4E-04	7.1E-05
CoA_divalent	5.5E-03	4.2E-03	6.2E-03	5.9E-03	6.1E-03	7.6E-03	5.1E-03	6.3E-03	7.1E-03	8.0E-03	8.0E-03	7.3E-03	5.3E-03	9.9E-04	6.6E-03	9.4E-04	6.1E-03	1.0E-03	7.7E-03	4.3E-04
Glutathione	3.8E-01	3.4E-01	4.5E-01	4.5E-01	5.0E-01	5.1E-01	4.0E-01	3.9E-01	5.0E-01	5.8E-01	6.4E-01	5.5E-01	3.9E-01	5.4E-02	4.8E-01	3.4E-02	4.3E-01	5.9E-02	5.9E-01	4.1E-02
Lactic acid	4.0E-01	4.0E-01	4.8E-01	4.6E-01	5.4E-01	5.6E-01	4.4E-01	4.0E-01	4.7E-01	5.4E-01	5.8E-01	5.2E-01	4.3E-01	5.0E-02	5.2E-01	5.3E-02	4.4E-01	3.3E-02	5.5E-01	3.5E-02
NMN	3.3E-04	4.3E-04	3.8E-04	4.1E-04	4.5E-04	5.0E-04	3.4E-04	3.6E-04	3.8E-04	4.3E-04	3.9E-04	3.6E-04	3.8E-04	5.1E-05	4.6E-04	4.6E-05	3.6E-04	1.9E-05	3.9E-04	3.2E-05
Gly	1.2E-01	1.1E-01	1.2E-01	1.2E-01	1.5E-01	1.5E-01	1.1E-01	1.3E-01	1.2E-01	1.6E-01	1.4E-01	1.4E-01	1.1E-01	5.7E-03	1.4E-01	1.8E-02	1.2E-01	8.6E-03	1.4E-01	9.4E-03
XC0089	1.1E-04	1.3E-04	1.0E-04	1.0E-04	1.5E-04	1.6E-04	1.1E-04	1.1E-04	1.3E-04	1.0E-04	9.0E-05	9.9E-05	1.1E-04	1.3E-05	1.4E-04	2.9E-05	1.2E-04	1.1E-05	9.8E-05	7.4E-06
Nicotinamide	6.2E-02	6.3E-02	7.6E-02	7.8E-02	8.5E-02	7.6E-02	7.6E-02	8.3E-02	6.5E-02	1.2E-01	1.2E-01	1.2E-01	6.7E-02	7.5E-03	8.0E-02	4.8E-03	7.5E-02	8.6E-03	1.2E-01	3.6E-03
3-Aminobutyric acid	5.3E-04	5.5E-04	6.1E-04	4.7E-04	7.9E-04	7.4E-04	6.3E-04	4.8E-04	5.4E-04	6.3E-04	4.8E-04	4.2E-04	5.6E-04	4.2E-05	6.7E-04	1.8E-04	5.5E-04	7.3E-05	5.1E-04	1.1E-04
Taurocyamine	3.2E-04	3.4E-04	3.9E-04	3.3E-04	3.9E-04	5.3E-04	3.3E-04	2.5E-04	4.1E-04	3.5E-04	3.7E-04	3.5E-04	3.5E-04	3.7E-05	4.1E-04	1.0E-04	3.3E-04	7.7E-05	3.6E-04	1.2E-05
Tyr	1.2E-02	1.0E-02	7.2E-03	1.1E-02	1.2E-02	1.2E-02	7.9E-03	9.6E-03	9.5E-03	1.6E-02	1.0E-02	1.3E-02	9.8E-03	2.4E-03	1.1E-02	5.7E-04	9.0E-03	9.2E-04	1.3E-02	2.7E-03
Thiamine	2.7E-04	2.6E-04	2.8E-04	3.2E-04	3.1E-04	3.1E-04	1.9E-04	2.5E-04	2.9E-04	2.7E-04	2.2E-04	2.2E-04	2.7E-04	8.0E-06	3.1E-04	4.2E-06	2.4E-04	4.8E-05	2.4E-04	2.5E-05
Isethionic acid	1.6E-03	1.6E-03	1.6E-03	1.7E-03	1.9E-03	1.9E-03	1.3E-03	1.4E-03	1.7E-03	1.6E-03	1.6E-03	1.3E-03	1.6E-03	2.6E-05	1.8E-03	7.5E-05	1.5E-03	2.1E-04	1.5E-03	1.4E-04
Guanosine	3.0E-03	2.7E-03	3.7E-03	2.4E-03	4.0E-03	4.5E-03	2.8E-03	2.7E-03	3.3E-03	3.4E-03	3.4E-03	3.2E-03	3.1E-03	5.1E-04	3.6E-03	1.1E-03	3.0E-03	3.4E-04	3.3E-03	1.1E-04
Pyruvic acid	2.6E-03	2.5E-03	3.3E-03	4.0E-03	2.7E-03	2.9E-03	2.4E-03	2.4E-03	3.1E-03	3.1E-03	3.5E-03	3.2E-03	2.8E-03	4.4E-04	3.2E-03	6.9E-04	2.6E-03	4.1E-04	3.3E-03	2.1E-04
myo-Inositol 1-phosphate *myo*-Inositol 3-phosphate	4.5E-04	4.7E-04	3.9E-04	6.5E-04	4.5E-04	3.9E-04	4.2E-04	5.0E-04	3.0E-04	4.9E-04	3.8E-04	4.5E-04	4.3E-04	3.8E-05	5.0E-04	1.4E-04	4.1E-04	1.0E-04	4.4E-04	5.8E-05
Uracil	2.8E-04	3.5E-04	3.1E-04	3.8E-04	3.7E-04	3.1E-04	3.7E-04	4.2E-04	3.2E-04	5.0E-04	3.8E-04	4.3E-04	3.1E-04	3.5E-05	3.6E-04	3.9E-05	3.7E-04	4.9E-05	4.3E-04	6.0E-05
UDP-*N*-acetylglucosami	1.3E-02	1.4E-02	1.6E-02	1.3E-02	1.8E-02	1.8E-02	1.3E-02	1.4E-02	1.7E-02	1.7E-02	1.4E-02	1.5E-02	1.4E-02	1.2E-03	1.6E-02	2.8E-03	1.5E-02	2.2E-03	1.5E-02	1.7E-03
$NADP^+$	4.0E-03	4.0E-03	4.4E-03	3.6E-03	5.2E-03	5.3E-03	3.5E-03	3.4E-03	4.2E-03	4.0E-03	3.2E-03	3.6E-03	4.1E-03	2.1E-04	4.7E-03	9.4E-04	3.7E-03	4.1E-04	3.6E-03	3.6E-04
Serotonin	4.2E-04	6.6E-04	1.9E-04	9.3E-04	2.8E-04	2.2E-04	4.2E-04	4.2E-04	1.9E-04	1.8E-04	2.3E-04	2.9E-04	4.2E-04	2.4E-04	4.8E-04	3.9E-04	3.4E-04	1.3E-04	2.3E-04	5.2E-05
Adenine	1.0E-03	6.9E-04	6.6E-04	8.2E-04	1.1E-03	8.1E-04	9.7E-04	9.5E-04	9.7E-04	8.2E-04	9.4E-04	7.6E-04	8.0E-04	2.2E-04	9.0E-04	1.5E-04	9.6E-04	1.3E-05	8.4E-04	9.2E-05
Thiaproline	6.8E-04	4.7E-04	4.7E-04	5.1E-04	7.0E-04	5.8E-04	5.6E-04	6.9E-04	4.7E-04	6.6E-04	5.7E-04	7.0E-04	5.4E-04	1.2E-04	6.0E-04	9.3E-05	5.7E-04	1.1E-04	6.4E-04	6.3E-05
Cysteine glutathione disulfide	1.6E-04	1.4E-04	1.3E-04	1.3E-04	1.8E-04	1.5E-04	1.7E-04	1.9E-04	2.1E-04	2.9E-04	2.5E-04	2.5E-04	1.4E-04	1.6E-05	1.6E-04	2.5E-05	1.9E-04	2.1E-05	2.6E-04	2.4E-05
Ribose 5-phosphate	3.1E-03	2.9E-03	4.3E-03	3.4E-03	4.2E-03	3.6E-03	2.4E-03	3.1E-03	3.4E-03	4.5E-03	4.2E-03	3.9E-03	3.4E-03	7.4E-04	3.7E-03	4.3E-04	3.0E-03	5.1E-04	4.2E-03	2.9E-04
Xanthine	2.0E-03	2.4E-03	2.6E-03	3.7E-03	2.3E-03	1.6E-03	2.8E-03	3.0E-03	2.9E-03	3.5E-03	2.7E-03	2.6E-03	2.3E-03	3.2E-04	2.5E-03	1.1E-03	2.9E-03	9.3E-05	2.9E-03	5.2E-04
Glycerol 3-phosphate	7.4E-02	6.7E-02	7.6E-02	8.2E-02	7.3E-02	8.1E-02	6.3E-02	6.8E-02	7.4E-02	8.7E-02	9.1E-02	8.1E-02	7.2E-02	4.7E-03	7.9E-02	4.8E-03	6.8E-02	5.8E-03	8.6E-02	4.7E-03
Spermine	4.9E-04	5.4E-04	5.7E-04	6.9E-04	5.5E-04	4.9E-04	6.0E-04	5.2E-04	4.7E-04	6.6E-04	5.3E-04	5.5E-04	5.3E-04	4.1E-05	5.8E-04	1.0E-04	5.3E-04	6.8E-05	5.8E-04	7.1E-05
Acetyl CoA_divalent	2.5E-04	2.2E-04	3.2E-04	3.6E-04	2.4E-04	2.6E-04	1.7E-04	3.1E-04	2.6E-04	2.7E-04	4.1E-04	1.6E-04	2.7E-04	5.1E-05	2.9E-04	6.5E-05	2.5E-04	6.7E-05	2.8E-04	1.3E-04
GDP-glucose	6.1E-04	5.7E-04	7.4E-04	6.0E-04	6.4E-04	8.3E-04	5.4E-04	5.6E-04	7.3E-04	6.5E-04	8.0E-04	6.4E-04	6.4E-04	8.6E-05	6.9E-04	1.2E-04	6.1E-04	1.1E-04	7.0E-04	8.9E-05
S-Methylglutathion	1.9E-03	1.6E-03	1.5E-03	1.5E-03	2.1E-03	1.8E-03	1.5E-03	1.3E-03	2.0E-03	2.2E-03	2.1E-03	2.0E-03	1.7E-03	2.0E-04	1.8E-03	3.0E-04	1.6E-03	3.8E-04	2.1E-03	9.6E-05
Pelargonic acid	3.8E-04	4.7E-04	4.2E-04	4.2E-04	5.3E-04	4.2E-04	4.9E-04	4.2E-04	4.1E-04	4.2E-04	3.7E-04	4.4E-04	4.2E-04	4.3E-05	4.5E-04	6.3E-05	4.4E-04	4.5E-05	4.1E-04	3.6E-05
6-Phosphogluconic acid	1.7E-03	1.8E-03	2.0E-03	1.7E-03	2.0E-03	2.2E-03	1.3E-03	1.7E-03	1.6E-03	2.2E-03	2.4E-03	2.1E-03	1.8E-03	1.4E-04	1.9E-03	2.6E-04	1.6E-03	2.1E-04	2.2E-03	1.3E-04
3-Indoxylsulfuric acid	2.8E-04	2.5E-04	2.7E-04	3.9E-04	2.5E-04	2.1E-04	3.4E-04	3.1E-04	1.9E-04	3.8E-04	3.3E-04	2.5E-04	2.7E-04	1.7E-05	2.8E-04	9.5E-05	2.8E-04	8.1E-05	3.2E-04	6.3E-05
Theobromine	N.D.	4.9E-04	4.6E-04	5.1E-04	5.3E-04	4.9E-04	4.1E-04	3.7E-04	3.7E-04	5.2E-04	4.6E-04	4.8E-04	4.8E-04	2.0E-05	5.1E-04	2.1E-05	3.8E-04	2.1E-05	4.9E-04	3.2E-05
Glucosamine	2.5E-04	3.1E-04	1.8E-04	3.5E-04	2.2E-04	2.3E-04	3.5E-04	3.6E-04	3.4E-04	2.5E-04	2.5E-04	2.6E-04	2.5E-04	6.5E-05	2.7E-04	7.2E-05	3.5E-04	1.1E-05	2.5E-04	6.4E-06

Comparative Analysis

Compound name	rad vs control			sake vs control			sake+rad vs control			sake+rad vs rad			sake+rad vs sake		
	Ratio ¶	*p*-value ‖		Ratio ¶	*p*-value ‖		Ratio ¶	*p*-value ‖		Ratio ¶	*p*-value ‖		Ratio ¶	*p*-value ‖	
Octanoic acid	1<	N.A.		1<	N.A.		N.A.	N.A.		<1	N.A.		<1	N.A.	
XA0013	1<	N.A.		1<	N.A.		N.A.	N.A.		<1	N.A.		<1	N.A.	
Fructose 1,6-diphosphate	4.2	0.378		0.9	0.317		1.1	0.713		0.2	0.385		1.2	0.325	
XA0055	3.1	0.305		0.8	0.554		1.7	0.403		0.5	0.464		2.0	0.315	
UDP-glucose UDP-galactose	2.7	0.018	*	0.9	0.743		1.6	0.047	*	0.6	0.072		1.8	0.004	**
Taurocholic acid	2.1	0.158		1.2	0.610		0.8	0.428		0.4	0.106		0.6	0.382	
3',5'-ADP	1.9	0.154		1.0	0.629		0.7	0.011	*	0.4	0.093		0.7	0.015	*
Dihydroxyacetone phosphate	1.9	0.380		0.8	0.413		1.0	0.930		0.6	0.407		1.3	0.588	
S-Lactoylglutathion	1.8	0.298		2.4	0.277		1.6	0.336		0.9	0.793		0.7	0.493	
Glycerophosphocholine	1.7	0.111		1.0	0.827		1.6	0.059		0.9	0.698		1.5	0.035	*
5,6,7,8-Tetrahydrobiopterin	1.7	N.A.		1.0	N.A.		1.1	N.A.		0.7	N.A.		1.2	N.A.	
Triethanolamine	1.6	0.191		5.0	0.419		1.0	0.813		0.6	0.211		0.2	0.423	
γ-Glu-2-aminobutyric	1.6	0.053		0.9	0.665		1.1	0.723		0.7	0.071		1.2	0.074	
Cys	1.6	0.014	*	1.4	0.129		3.0	4.1E-04	***	1.9	0.001	**	2.1	0.004	**
Inosine	1.6	0.070		1.3	0.139		1.7	0.015	*	1.1	0.699		1.3	0.078	
Phosphoenolpyruvic acid	1.6	0.033	*	0.7	0.131		1.1	0.608		0.7	0.063		1.5	0.094	
Creatine	1.5	0.002	**	0.8	0.044	*	1.3	0.156		0.9	0.345		1.6	0.087	
3-Phosphoglyceric	1.5	0.020	*	0.7	0.053		1.1	0.550		0.7	0.041	*	1.6	0.036	*
XA0035	1.5	0.055		0.9	0.260		1.1	0.244		0.8	0.078		1.3	0.088	
Homovanillic	1.5	0.362		1.1	0.633		1.4	0.212		0.9	0.805		1.3	0.283	
Ascorbic acid	1.4	0.184		1.0	0.935		1.6	0.017	*	1.1	0.510		1.6	0.072	
3'-Dephospho	1.4	0.124		1.3	0.248		2.8	0.004	**	2.0	0.008	**	2.1	0.007	**
N-Acetylglucosami	1.4	0.255		0.9	0.457		1.2	0.278		0.8	0.458		1.3	0.044	*
2,3-Diphosphoglyceric acid	1.4	0.405		0.9	0.543		0.9	0.574		0.6	0.312		1.0	0.861	
5-Oxoproline	1.4	0.264		1.2	0.280		1.5	0.019	*	1.1	0.613		1.3	0.116	
Ribulose 5-phosphate	1.4	0.100		1.0	0.337		1.3	0.027	*	1.0	0.954		1.3	0.027	*
Fructose 6-phosphate	1.4	0.356		1.1	0.410		0.7	0.193		0.5	0.140		0.6	0.119	
Adenosine	1.3	0.260		0.8	0.148		1.0	0.899		0.8	0.277		1.3	0.163	
XC0126	1.3	0.263		1.0	0.839		1.3	0.141		1.0	0.919		1.3	0.108	
Gln	1.3	0.115		0.9	0.302		1.1	0.082		0.9	0.312		1.2	0.035	*
Hexanoic acid	1.3	N.A.		2.7	0.099		1.2	0.384		0.9	N.A.		0.4	0.112	
Hypoxanthine	1.2	0.574		1.4	0.012	*	2.0	0.017	*	1.6	0.182		1.4	0.061	
N-	1.2	0.043	*	1.0	0.628		1.1	0.450		0.9	0.136		1.1	0.298	
CoA_divalent	1.2	0.183		1.2	0.356		1.5	0.035	*	1.2	0.150		1.3	0.092	
Glutathione	1.2	0.077		1.1	0.467		1.5	0.008	**	1.2	0.027	*	1.4	0.022	*
Lactic acid	1.2	0.082		1.0	0.751		1.3	0.033	*	1.0	0.591		1.2	0.018	*
NMN	1.2	0.121		0.9	0.580		1.0	0.707		0.9	0.127		1.1	0.201	
Gly	1.2	0.148		1.1	0.348		1.3	0.013	*	1.1	0.559		1.2	0.029	*
XC0089	1.2	0.313		1.0	0.722		0.9	0.168		0.7	0.141		0.8	0.064	
Nicotinamide	1.2	0.078		1.1	0.312		1.8	0.002	**	1.5	4.4E-04	***	1.6	0.005	**
3-Aminobutyric acid	1.2	0.411		1.0	0.850		0.9	0.490		0.8	0.269		0.9	0.602	
Taurocyamine	1.2	0.391		0.9	0.695		1.0	0.780		0.9	0.432		1.1	0.589	
Tyr	1.2	0.366		0.9	0.647		1.3	0.228		1.1	0.475		1.4	0.128	
Thiamine	1.2	0.004	**	0.9	0.438		0.9	0.133		0.8	0.031	*	1.0	0.838	
Isethionic acid	1.2	0.020	*	0.9	0.547		1.0	0.439		0.8	0.038	*	1.0	0.968	
Guanosine	1.2	0.536		0.9	0.646		1.1	0.616		0.9	0.671		1.1	0.205	
Pyruvic acid	1.2	0.432		0.9	0.708		1.2	0.185		1.0	0.883		1.2	0.099	
myo-Inositol 1-phosphate *myo*-Inositol 3-phosphate	1.1	0.516		0.9	0.716		1.0	0.897		0.9	0.559		1.1	0.675	
Uracil	1.1	0.214		1.2	0.192		1.4	0.050		1.2	0.144		1.2	0.215	
UDP-*N*-acetylglucosami	1.1	0.345		1.0	0.739		1.1	0.501		0.9	0.585		1.0	0.830	
$NADP^+$	1.1	0.419		0.9	0.182		0.9	0.102		0.8	0.173		1.0	0.805	
Serotonin	1.1	0.850		0.8	0.639		0.6	0.297		0.5	0.396		0.7	0.282	
Adenine	1.1	0.548		1.2	0.312		1.1	0.789		0.9	0.588		0.9	0.134	
Thiaproline	1.1	0.540		1.1	0.740		1.2	0.285		1.1	0.553		1.1	0.415	
Cysteine glutathione disulfide	1.1	0.445		1.4	0.030	*	1.8	0.003	**	1.7	0.007	**	1.4	0.021	*
Ribose 5-phosphate	1.1	0.539		0.9	0.484		1.2	0.207		1.1	0.234		1.4	0.037	*
Xanthine	1.1	0.758		1.2	0.080		1.3	0.162		1.2	0.598		1.0	0.875	
Glycerol 3-phosphate	1.1	0.169		0.9	0.404		1.2	0.022	*	1.1	0.131		1.3	0.015	*
Spermine	1.1	0.552		1.0	0.926		1.1	0.392		1.0	0.965		1.1	0.415	
Acetyl CoA_divalent	1.1	0.679		0.9	0.724		1.0	0.878		1.0	0.928		1.1	0.725	
GDP-glucose	1.1	0.591		1.0	0.707		1.1	0.474		1.0	0.954		1.1	0.333	
S-Methylglutathion	1.1	0.577		1.0	0.843		1.3	0.047	*	1.2	0.218		1.3	0.148	
Pelargonic acid	1.1	0.510		1.0	0.608		1.0	0.785		0.9	0.390		0.9	0.430	
6-Phosphogluconic acid	1.1	0.497		0.9	0.179		1.2	0.020	*	1.2	0.182		1.4	0.013	*
3-Indoxylsulfuric acid	1.1	0.771		1.1	0.770		1.2	0.283		1.1	0.633		1.1	0.569	
Theobromine	1.1	0.200		0.8	0.024	*	1.0	0.678		1.0	0.392		1.3	0.013	*
Glucosamine	1.1	0.788		1.4	0.115		1.0	0.915		1.0	0.806		0.7	7.5E-04	***

XA0019	4.9E-03	2.7E-03	2.6E-03	2.3E-03	5.1E-03	3.4E-03	3.1E-03	3.7E-03	2.7E-03	2.7E-03	2.5E-03	3.3E-03	3.4E-03	1.3E-03	3.6E-03	1.4E-03	3.1E-03	4.7E-04	2.8E-03	4.4E-04	1.1	0.856	0.9	0.778		0.8	0.529		0.8	0.451		0.9	0.437	
Cytidine	3.2E-04	3.6E-04	3.2E-04	3.6E-04	3.9E-04	3.2E-04	3.5E-04	2.9E-04	3.3E-04	3.2E-04	3.8E-04	4.0E-04	3.3E-04	2.6E-05	3.6E-04	3.4E-05	3.2E-04	3.1E-05	3.6E-04	4.2E-05	1.1	0.447	1.0	0.709		1.1	0.365		1.0	0.782		1.1	0.271	
CDP-choline	4.2E-04	5.6E-04	4.8E-04	3.3E-04	5.7E-04	6.4E-04	6.1E-04	5.6E-04	4.3E-04	5.1E-04	4.7E-04	5.0E-04	4.8E-04	7.1E-05	5.1E-04	1.6E-04	5.3E-04	9.7E-05	4.9E-04	2.3E-05	1.1	0.815	1.1	0.532		1.0	0.875		1.0	0.860		0.9	0.550	
Uridine	3.7E-03	4.1E-03	4.0E-03	4.2E-03	4.2E-03	4.1E-03	4.5E-03	5.4E-03	4.4E-03	5.5E-03	4.9E-03	5.0E-03	3.9E-03	2.2E-04	4.2E-03	2.5E-05	4.8E-03	5.3E-04	5.2E-03	3.0E-04	1.1	0.242	1.2	0.102		1.3	0.006	**	1.2	0.028	*	1.1	0.348	
CMP	7.5E-04	8.9E-04	7.0E-04	5.6E-04	9.8E-04	9.2E-04	8.1E-04	7.9E-04	7.4E-04	1.1E-03	1.1E-03	1.0E-03	7.8E-04	1.0E-04	8.2E-04	2.2E-04	7.8E-04	3.4E-05	1.0E-03	3.3E-05	1.1	0.798	1.0	0.992		1.3	0.034	*	1.3	0.224		1.3	6.7E-04	***
Trp	8.2E-03	7.9E-03	5.2E-03	7.6E-03	8.0E-03	6.7E-03	6.9E-03	6.8E-03	7.6E-03	7.6E-03	5.5E-03	6.5E-03	7.1E-03	1.6E-03	7.4E-03	7.1E-04	7.1E-03	4.2E-04	6.5E-03	1.0E-03	1.1	0.755	1.0	0.975		0.9	0.659		0.9	0.285		0.9	0.439	
Ala	4.9E-01	3.4E-01	3.5E-01	4.1E-01	4.1E-01	4.2E-01	3.4E-01	3.8E-01	4.0E-01	4.4E-01	3.7E-01	3.8E-01	3.9E-01	8.3E-02	4.1E-01	7.0E-03	3.7E-01	3.0E-02	4.0E-01	4.2E-02	1.0	0.728	1.0	0.735		1.0	0.972		1.0	0.552		1.1	0.516	
XC0132	5.6E-04	5.9E-04	6.5E-04	5.7E-04	6.8E-04	6.4E-04	5.2E-04	5.6E-04	5.7E-04	6.5E-04	6.9E-04	6.6E-04	6.0E-04	4.9E-05	6.3E-04	5.5E-05	5.5E-04	2.6E-05	6.6E-04	2.3E-05	1.0	0.566	0.9	0.222		1.1	0.140		1.1	0.370		1.2	0.005	**
XC0016	9.2E-04	7.4E-04	8.4E-04	6.4E-04	1.0E-03	9.5E-04	7.2E-04	6.7E-04	7.6E-04	1.1E-03	9.8E-04	8.8E-04	8.4E-04	8.7E-05	8.7E-04	2.0E-04	7.2E-04	4.2E-05	9.7E-04	9.4E-05	1.0	0.804	0.9	0.128		1.2	0.135		1.1	0.482		1.4	0.027	*
XA0003	3.8E-04	2.2E-04	4.9E-04	7.7E-04	2.2E-04	1.4E-04	1.6E-04	3.4E-04	3.3E-04	3.7E-04	9.4E-04	6.5E-04	3.6E-04	1.4E-04	3.8E-04	3.4E-04	2.7E-04	1.0E-04	6.5E-04	2.9E-04	1.0	0.950	0.8	0.427		1.8	0.216		1.7	0.350		2.4	0.137	
Gly-Asp	2.1E-04	2.0E-04	2.5E-04	1.8E-04	2.8E-04	2.1E-04	2.0E-04	1.9E-04	2.3E-04	2.7E-04	2.3E-04	2.4E-04	2.2E-04	2.6E-05	2.2E-04	4.8E-05	2.1E-04	1.7E-05	2.5E-04	2.1E-05	1.0	0.859	1.0	0.594		1.1	0.219		1.1	0.525		1.2	0.072	
Thiamine phosphate	8.1E-04	7.9E-04	8.8E-04	6.8E-04	8.6E-04	1.0E-03	6.5E-04	5.4E-04	7.3E-04	9.8E-04	9.6E-04	1.0E-03	8.3E-04	4.8E-05	8.5E-04	1.7E-04	6.4E-04	9.8E-05	9.9E-04	4.6E-05	1.0	0.840	0.8	0.060		1.2	0.013	*	1.2	0.283		1.5	0.013	*
Xanthosine	3.3E-04	5.0E-04	3.7E-04	6.8E-04	2.9E-04	2.5E-04	4.2E-04	4.8E-04	4.2E-04	4.4E-04	3.3E-04	2.9E-04	4.0E-04	8.9E-05	4.1E-04	2.4E-04	4.4E-04	3.5E-05	3.5E-04	7.7E-05	1.0	0.964	1.1	0.548		0.9	0.515		0.9	0.733		0.8	0.181	
Cysteinesulfinic acid	1.1E-03	8.1E-04	5.8E-04	6.3E-04	7.7E-04	1.1E-03	6.6E-04	7.3E-04	8.0E-04	8.8E-04	7.5E-04	7.0E-04	8.3E-04	2.6E-04	8.4E-04	2.5E-04	7.3E-04	6.9E-05	7.8E-04	9.1E-05	1.0	0.958	0.9	0.579		0.9	0.779		0.9	0.722		1.1	0.492	
CMP-*N*-acetylneuraminat	4.9E-03	4.8E-03	4.1E-03	3.4E-03	5.7E-03	4.9E-03	4.9E-03	4.2E-03	4.2E-03	4.3E-03	3.6E-03	4.6E-03	4.6E-03	4.5E-04	4.7E-03	1.2E-03	4.4E-03	3.8E-04	4.2E-03	5.1E-04	1.0	0.938	1.0	0.642		0.9	0.317		0.9	0.536		0.9	0.493	
His	7.6E-02	6.5E-02	7.9E-02	7.6E-02	7.1E-02	7.4E-02	7.2E-02	7.7E-02	7.8E-02	8.4E-02	7.3E-02	7.8E-02	7.3E-02	7.5E-03	7.4E-02	2.5E-03	7.5E-02	3.4E-03	7.8E-02	5.5E-03	1.0	0.882	1.0	0.682		1.1	0.420		1.1	0.325		1.0	0.514	
Gly-Gly	4.0E-04	4.0E-04	3.8E-04	3.5E-04	4.5E-04	3.9E-04	3.9E-04	4.2E-04	4.5E-04	7.5E-04	6.1E-04	6.5E-04	3.9E-04	7.6E-06	4.0E-04	5.1E-05	4.2E-04	3.1E-05	6.7E-04	7.6E-05	1.0	0.924	1.1	0.286		1.7	0.023	*	1.7	0.009	**	1.6	0.018	*
Glucose 6-phosphate	3.5E-02	2.2E-02	3.1E-02	3.4E-02	2.4E-02	2.9E-02	3.1E-02	3.4E-02	3.5E-02	1.2E-02	2.5E-02	2.0E-02	2.9E-02	6.6E-03	2.9E-02	4.7E-03	3.3E-02	2.2E-03	1.9E-02	6.6E-03	1.0	0.963	1.1	0.387		0.7	0.140		0.7	0.115		0.6	0.053	
Glucose 1-phosphate	3.4E-03	2.0E-03	3.2E-03	3.8E-03	2.3E-03	2.5E-03	2.7E-03	3.3E-03	3.1E-03	1.3E-03	3.1E-03	2.3E-03	2.9E-03	7.5E-04	2.9E-03	7.8E-04	3.0E-03	2.9E-04	2.2E-03	8.9E-04	1.0	0.963	1.0	0.825		0.8	0.379		0.8	0.406		0.7	0.266	
AMP	1.1E-01	1.1E-01	1.2E-01	9.7E-02	1.2E-01	1.2E-01	1.2E-01	1.2E-01	1.2E-01	1.7E-01	1.6E-01	1.6E-01	1.1E-01	2.5E-03	1.1E-01	1.3E-02	1.2E-01	3.0E-03	1.6E-01	6.4E-03	1.0	0.846	1.1	0.053		1.4	0.002	**	1.4	0.010	**	1.4	0.002	**
Creatinine	1.3E-03	1.3E-03	9.9E-04	1.2E-03	1.4E-03	1.1E-03	1.1E-03	1.1E-03	1.1E-03	1.4E-03	1.0E-03	1.1E-03	1.2E-03	2.1E-04	1.2E-03	1.5E-04	1.1E-03	4.6E-05	1.2E-03	2.2E-04	1.0	0.881	0.9	0.439		1.0	0.844		1.0	0.936		1.1	0.609	
Asn	7.0E-03	6.5E-03	5.3E-03	5.9E-03	6.6E-03	5.9E-03	5.9E-03	6.6E-03	6.3E-03	7.4E-03	5.9E-03	6.7E-03	6.3E-03	8.5E-04	6.1E-03	4.0E-04	6.3E-03	3.7E-04	6.7E-03	7.3E-04	1.0	0.834	1.0	0.976		1.1	0.539		1.1	0.326		1.1	0.442	
Kynurenine	1.5E-04	1.6E-04	1.6E-04	2.1E-04	1.7E-04	8.5E-05	1.4E-04	1.2E-04	1.5E-04	1.7E-04	2.5E-04	2.6E-04	1.6E-04	9.1E-06	1.5E-04	6.2E-05	1.4E-04	1.1E-05	2.3E-04	4.7E-05	1.0	0.932	0.9	0.079		1.5	0.112		1.5	0.170		1.7	0.070	
FAD_divalent	1.2E-03	1.1E-03	1.2E-03	9.6E-04	1.2E-03	1.3E-03	1.1E-03	1.2E-03	1.0E-03	1.3E-03	1.3E-03	1.1E-03	1.2E-03	2.9E-05	1.1E-03	1.6E-04	1.1E-03	8.4E-05	1.2E-03	1.1E-04	1.0	0.800	0.9	0.305		1.1	0.412		1.1	0.464		1.1	0.181	
N^8-Acetylspermidine	2.3E-04	1.3E-04	1.0E-04	1.4E-04	1.7E-04	1.4E-04	1.5E-04	1.2E-04	1.7E-04	2.6E-04	1.6E-04	1.8E-04	1.5E-04	7.0E-05	1.5E-04	1.4E-05	1.5E-04	2.7E-05	2.0E-04	5.1E-05	1.0	0.921	1.0	0.916		1.3	0.421		1.3	0.229		1.3	0.229	
Guanidinosuccinic acid	2.5E-03	1.8E-03	2.4E-03	1.6E-03	2.3E-03	2.5E-03	1.5E-03	1.4E-03	1.8E-03	2.0E-03	1.6E-03	1.9E-03	2.2E-03	3.6E-04	2.1E-03	4.8E-04	1.6E-03	2.2E-04	1.8E-03	2.3E-04	1.0	0.851	0.7	0.069		0.8	0.232		0.9	0.425		1.2	0.188	
Phe	4.0E-02	3.8E-02	2.9E-02	3.1E-02	3.8E-02	3.5E-02	3.5E-02	3.6E-02	4.0E-02	4.2E-02	3.2E-02	3.4E-02	3.6E-02	5.7E-03	3.5E-02	3.6E-03	3.7E-02	2.3E-03	3.6E-02	5.1E-03	1.0	0.764	1.0	0.760		1.0	0.968		1.0	0.709		1.0	0.778	
Pyridoxal	1.6E-04	1.4E-04	1.6E-04	1.1E-04	1.6E-04	1.7E-04	1.5E-04	1.4E-04	1.5E-04	1.3E-04	1.4E-04	1.4E-04	1.5E-04	1.4E-05	1.5E-04	3.2E-05	1.5E-04	4.2E-06	1.4E-04	7.9E-06	1.0	0.807	1.0	0.698		0.9	0.183		0.9	0.636		0.9	0.100	
N-Glycolylneuraminic acid	1.1E-03	1.1E-03	1.2E-03	9.0E-04	9.7E-04	1.4E-03	1.0E-03	1.1E-03	1.4E-03	1.3E-03	1.2E-03	1.2E-03	1.1E-03	5.0E-05	1.1E-03	2.7E-04	1.2E-03	1.8E-04	1.2E-03	4.2E-05	1.0	0.813	1.0	0.771		1.1	0.094		1.1	0.505		1.0	0.697	
Threonic acid	8.2E-04	6.4E-04	7.0E-04	6.1E-04	7.5E-04	7.1E-04	6.4E-04	6.8E-04	6.3E-04	6.9E-04	6.4E-04	6.4E-04	7.2E-04	9.1E-05	6.9E-04	7.1E-05	6.5E-04	2.6E-05	6.6E-04	3.0E-05	1.0	0.671	0.9	0.314		0.9	0.364		1.0	0.540		1.0	0.741	
Ala-Ala	7.2E-04	6.1E-04	7.2E-04	4.9E-04	7.3E-04	7.4E-04	7.2E-04	7.5E-04	8.1E-04	9.6E-04	9.1E-04	9.3E-04	6.8E-04	6.4E-05	6.5E-04	1.4E-04	7.6E-04	4.7E-05	9.3E-04	2.6E-05	1.0	0.741	1.1	0.180		1.4	0.012	*	1.4	0.069		1.2	0.010	*
Thiamine diphosphate	5.2E-04	5.7E-04	5.3E-04	4.5E-04	5.6E-04	5.4E-04	4.4E-04	5.0E-04	4.9E-04	4.8E-04	3.6E-04	3.8E-04	5.4E-04	2.7E-05	5.1E-04	6.0E-05	4.8E-04	3.1E-05	4.1E-04	6.1E-05	0.9	0.514	0.9	0.057		0.8	0.046	*	0.8	0.098		0.9	0.172	
Diethanolamine	2.0E-04	1.7E-04	2.8E-04	3.0E-04	1.7E-04	1.4E-04	3.1E-04	1.1E-04	1.4E-04	1.4E-04	2.3E-04	1.7E-04	2.1E-04	5.7E-05	2.0E-04	8.5E-05	1.8E-04	1.1E-04	1.8E-04	4.4E-05	0.9	0.851	0.9	0.704		0.8	0.439		0.9	0.695		1.0	0.932	
NAD^+	1.6E-02	2.5E-02	2.5E-02	1.5E-02	2.3E-02	2.4E-02	2.0E-02	2.1E-02	2.3E-02	1.6E-02	1.5E-02	1.6E-02	2.2E-02	5.3E-03	2.1E-02	5.3E-03	2.1E-02	1.3E-03	1.6E-02	5.6E-04	0.9	0.786	1.0	0.854		0.7	0.172		0.8	0.237		0.7	0.009	**
XC0137	5.6E-03	5.5E-03	6.5E-03	5.3E-03	5.8E-03	5.6E-03	5.6E-03	5.2E-03	6.0E-03	2.9E-03	3.2E-03	3.3E-03	5.9E-03	5.6E-04	5.5E-03	2.5E-04	5.6E-03	3.8E-04	3.1E-03	1.8E-04	0.9	0.391	0.9	0.469		0.5	0.008	**	0.6	2.7E-04	***	0.6	0.003	**
Gly-Leu	4.9E-04	4.6E-04	5.5E-04	3.9E-04	5.1E-04	5.0E-04	5.3E-04	4.8E-04	4.8E-04	5.7E-04	4.6E-04	3.7E-04	5.0E-04	4.7E-05	4.7E-04	6.9E-05	5.0E-04	2.8E-05	4.7E-04	9.8E-05	0.9	0.534	1.0	0.916		0.9	0.622		1.0	0.986		0.9	0.644	
XA0065	1.9E-03	2.4E-03	3.0E-03	1.8E-03	2.6E-03	2.3E-03	1.9E-03	2.4E-03	2.1E-03	2.0E-03	1.8E-03	2.4E-03	2.4E-03	5.8E-04	2.2E-03	3.7E-04	2.1E-03	2.5E-04	2.1E-03	2.7E-04	0.9	0.672	0.9	0.495		0.9	0.413		0.9	0.560		1.0	0.764	
Ergothioneine	2.7E-02	3.1E-02	3.4E-02	2.1E-02	3.5E-02	2.9E-02	2.7E-02	2.5E-02	2.5E-02	2.9E-02	2.5E-02	2.6E-02	3.1E-02	3.6E-03	2.8E-02	7.2E-03	2.6E-02	1.4E-03	2.7E-02	2.2E-03	0.9	0.647	0.8	0.138		0.9	0.200		0.9	0.750		1.0	0.605	
Lauric acid	9.1E-04	9.3E-04	9.8E-04	8.4E-04	9.6E-04	8.0E-04	7.1E-04	8.3E-04	7.9E-04	7.5E-04	7.6E-04	6.3E-04	9.4E-04	3.8E-05	8.7E-04	8.4E-05	7.8E-04	6.4E-05	7.1E-04	7.1E-05	0.9	0.266	0.8	0.028	*	0.8	0.016	*	0.8	0.074		0.9	0.297	
ADP	5.1E-02	5.1E-02	5.4E-02	3.7E-02	5.1E-02	5.6E-02	4.3E-02	4.3E-02	5.5E-02	3.8E-02	3.8E-02	3.8E-02	5.2E-02	1.4E-03	4.8E-02	1.0E-02	4.7E-02	6.6E-03	3.8E-02	1.0E-04	0.9	0.537	0.9	0.308		0.7	0.003	**	0.8	0.239		0.8	0.148	
Glutathione (GSSG)_divalent	2.3E-01	2.5E-01	2.1E-01	1.6E-01	2.7E-01	2.1E-01	2.6E-01	2.1E-01	2.1E-01	1.9E-01	1.5E-01	1.9E-01	2.3E-01	2.0E-02	2.1E-01	5.1E-02	2.3E-01	3.1E-02	1.7E-01	2.1E-02	0.9	0.585	1.0	0.808		0.7	0.024	*	0.8	0.306		0.8	0.074	
Ethanolamine phosphate	5.3E-03	6.7E-03	6.1E-03	4.6E-03	5.6E-03	6.2E-03	5.1E-03	5.9E-03	5.7E-03	5.5E-03	5.3E-03	5.3E-03	6.0E-03	6.9E-04	5.5E-03	8.0E-04	5.6E-03	4.1E-04	5.4E-03	1.4E-04	0.9	0.408	0.9	0.426		0.9	0.243		1.0	0.874		1.0	0.445	
Pyridoxamine 5'-phosphate	1.1E-03	1.0E-03	1.1E-03	8.6E-04	1.1E-03	1.0E-03	1.0E-03	9.7E-04	1.1E-03	1.2E-03	1.1E-03	9.2E-04	1.1E-03	7.0E-05	9.8E-04	1.0E-04	1.0E-03	5.4E-05	1.1E-03	1.3E-04	0.9	0.231	0.9	0.278		1.0	0.775		1.1	0.452		1.0	0.665	
ATP	4.5E-02	4.8E-02	5.2E-02	2.8E-02	4.7E-02	5.6E-02	3.1E-02	3.1E-02	4.0E-02	1.3E-02	1.6E-02	1.8E-02	4.8E-02	3.5E-03	4.4E-02	1.4E-02	3.4E-02	5.6E-03	1.6E-02	2.8E-03	0.9	0.622	0.7	0.027	*	0.3	3.0E-04	***	0.4	0.073		0.5	0.016	*
Ethanolamine	7.2E-04	1.0E-03	9.2E-04	8.6E-04	8.2E-04	6.8E-04	1.1E-03	1.2E-03	9.0E-04	1.1E-03	9.8E-04	9.2E-04	8.8E-04	1.5E-04	7.9E-04	9.3E-05	1.0E-03	1.4E-04	1.0E-03	1.0E-04	0.9	0.426	1.2	0.234		1.1	0.301		1.3	0.055		1.0	0.720	
Carnitine	7.8E-02	9.1E-02	1.0E-01	5.3E-02	1.0E-01	8.8E-02	7.3E-02	6.5E-02	7.7E-02	9.6E-02	9.7E-02	8.7E-02	9.0E-02	1.1E-02	8.0E-02	2.4E-02	7.2E-02	6.2E-03	9.3E-02	5.4E-03	0.9	0.585	0.8	0.088		1.0	0.671		1.2	0.457		1.3	0.011	*
Spermidine	3.3E-03	3.2E-03	2.7E-03	3.4E-03	2.9E-03	1.9E-03	3.4E-03	3.1E-03	2.8E-03	2.6E-03	2.5E-03	2.6E-03	3.1E-03	3.4E-04	2.7E-03	7.5E-04	3.1E-03	3.0E-04	2.6E-03	8.5E-05	0.9	0.539	1.0	0.978		0.8	0.111		0.9	0.715		0.8	0.087	
Thr	4.8E-02	3.9E-02	3.0E-02	3.3E-02	3.9E-02	3.3E-02	3.7E-02	4.2E-02	4.1E-02	4.8E-02	4.0E-02	4.1E-02	3.9E-02	8.9E-03	3.5E-02	3.8E-03	4.0E-02	2.5E-03	4.3E-02	4.1E-03	0.9	0.501	1.0	0.836		1.1	0.550		1.2	0.066		1.1	0.413	
Fumaric acid	1.1E-02	8.8E-03	8.5E-03	8.1E-03	8.3E-03	8.4E-03	6.6E-03	6.9E-03	7.1E-03	7.3E-03	6.2E-03	6.5E-03	9.4E-03	1.3E-03	8.2E-03	1.6E-04	6.9E-03	2.4E-04	6.7E-03	5.4E-04	0.9	0.266	0.7	0.075		0.7	0.052		0.8	0.028	*	1.0	0.550	
Citric acid	6.0E-03	6.2E-03	6.3E-03	5.8E-03	5.9E-03	4.5E-03	4.8E-03	5.1E-03	4.9E-03	5.7E-03	4.9E-03	6.2E-03	6.2E-03	1.3E-04	5.4E-03	7.9E-04	4.9E-03	1.7E-04	5.6E-03	6.5E-04	0.9	0.241	0.8	7.4E-04	***	0.9	0.272		1.0	0.773		1.1	0.215	
UDP	2.7E-03	2.8E-03	3.0E-03	1.4E-03	2.7E-03	3.4E-03	2.2E-03	2.3E-03	3.1E-03	1.6E-03	1.3E-03	1.2E-03	2.8E-03	1.5E-04	2.5E-03	1.0E-03	2.5E-03	4.7E-04	1.4E-03	2.1E-04	0.9	0.616	0.9	0.389		0.5	9.9E-04	***	0.5	0.191		0.5	0.034	*
Taurine	1.1E-01	1.2E-01	1.1E-01	9.3E-02	1.2E-01	8.9E-02	1.0E-01	9.0E-02	1.1E-01	1.2E-01	1.0E-01	1.0E-01	1.1E-01	6.4E-03	1.0E-01	1.6E-02	9.9E-02	8.5E-03	1.1E-01	7.0E-03	0.9	0.260	0.9	0.077		0.9	0.309		1.1	0.504		1.1	0.254	
threo-β-Methylaspartic acid	4.4E-04	3.8E-04	3.3E-04	3.4E-04	3.5E-04	3.2E-04	3.0E-04	3.0E-04	3.4E-04	3.1E-04	3.7E-04	2.2E-04	3.8E-04	5.3E-05	3.4E-04	1.8E-05	3.1E-04	2.4E-05	3.0E-04	7.6E-05	0.9	0.260	0.8	0.121		0.8	0.204		0.9	0.503		1.0	0.862	
GMP	9.9E-03	8.4E-03	9.7E-03	6.5E-03	9.5E-03	8.6E-03	9.4E-03	1.0E-02	1.1E-02	1.2E-02	1.2E-02	1.3E-02	9.3E-03	8.4E-04	8.2E-03	1.5E-03	1.0E-02	5.5E-04	1.2E-02	3.0E-05	0.9	0.335	1.1	0.324		1.3	0.023	*	1.5	0.040	*	1.2	0.016	*
Dyphylline	3.4E-03	4.4E-03	4.2E-03	4.0E-03	3.4E-03	3.1E-03	3.3E-03	3.1E-03	3.6E-03	4.1E-03	2.9E-03	3.7E-03	4.0E-03	4.9E-04	3.5E-03	4.6E-04	3.3E-03	3.0E-04	3.6E-03	5.9E-04	0.9	0.268	0.8	0.129		0.9	0.390		1.0	0.878		1.1	0.580	
Succinic acid	5.3E-02	4.5E-02	5.4E-02	2.7E-02	4.8E-02	5.7E-02	4.9E-02	4.8E-02	5.6E-02	5.5E-02	5.0E-02	4.5E-02	5.1E-02	4.7E-03	4.4E-02	1.5E-02	5.1E-02	4.2E-03	5.0E-02	4.8E-03	0.9	0.538	1.0	0.962		1.0	0.859		1.1	0.580		1.0	0.816	
1-Methyl-4-imidazoleacetic acid	2.6E-04	3.2E-04	3.3E-04	1.5E-04	3.3E-04	3.1E-04	2.8E-04	2.3E-04	2.8E-04	3.4E-04	3.6E-04	2.8E-04	3.0E-04	4.0E-05	2.6E-04	9.9E-05	2.6E-04	2.9E-05	3.2E-04	4.2E-05	0.9	0.571	0.9	0.230		1.1	0.547		1.2	0.403		1.2	0.108	
UTP	1.5E-03	1.8E-03	2.0E-03	8.0E-04	1.7E-03	2.1E-03	1.2E-03	1.1E-03	1.5E-03	4.5E-04	4.7E-04	4.6E-04	1.8E-03	2.2E-04	1.5E-03	6.8E-04	1.3E-03	2.4E-04	4.6E-04	7.6E-06	0.9	0.621	0.7	0.059		0.3	0.009	**	0.3	0.113		0.4	0.028	*
Ile	8.0E-02	7.0E-02	6.2E-02	5.9E-02	6.8E-02	5.6E-02	7.2E-02	7.2E-02	7.6E-02	7.5E-02	6.2E-02	6.7E-02	7.1E-02	9.2E-03	6.1E-02	6.2E-03	7.3E-02	2.3E-03	6.8E-02	6.7E-03	0.9	0.226	1.0	0.705		1.0	0.737		1.1	0.254		0.9	0.346	
Leu	1.4E-01	1.2E-01	1.1E-01	1.1E-01	1.2E-01	1.0E-01	1.2E-01	1.2E-01	1.3E-01	1.4E-01	1.2E-01	1.3E-01	1.3E-01	1.8E-02	1.1E-01	8.9E-03	1.3E-01	2.1E-03	1.3E-01	1.2E-02	0.9	0.238	1.0	0.955		1.0	0.903		1.2	0.099		1.0	0.901	
β-Ala-Lys	4.3E-04	3.7E-04	4.8E-04	2.9E-04	4.4E-04	3.7E-04	4.2E-04	4.7E-04	4.6E-04	4.4E-04	5.7E-04	4.6E-04	4.3E-04	5.4E-05	3.7E-04	7.4E-05	4.5E-04	3.0E-05	4.9E-04	7.1E-05	0.9	0.307	1.0	0.608		1.1	0.292		1.3	0.100		1.1	0.413	
Homoserinelactone	3.3E-04	3.4E-04	3.9E-04	2.7E-04	3.5E-04	2.9E-04	3.2E-04	2.7E-04	3.5E-04	3.3E-04	4.3E-04	3.7E-04	3.6E-04	3.3E-05	3.0E-04	4.4E-05	3.1E-04	3.6E-05	3.8E-04	5.1E-05	0.8	0.166	0.9	0.201		1.1	0.580		1.3	0.124		1.2	0.156	
Asp	7.9E-02	4.0E-02	3.2E-02	3.6E-02	4.3E-02	4.7E-02	2.8E-02	3.6E-02	3.9E-02	3.8E-02	2.8E-02	3.0E-02	5.0E-02	2.5E-02	4.2E-02	5.7E-03	3.4E-02	5.9E-03	3.2E-02	5.0E-03	0.8	0.651	0.7	0.388		0.6	0.336		0.8	0.077		0.9	0.638	
Guanidoacetic acid	1.2E-03	1.2E-03	1.3E-03	9.2E-04	1.1E-03	1.2E-03	1.5E-03	1.5E-03	1.6E-03	1.9E-03	1.5E-03	1.4E-03	1.3E-03	5.9E-05	1.1E-03	1.4E-04	1.5E-03	3.2E-05	1.6E-03	2.7E-04	0.8	0.129	1.2	0.005	**	1.3	0.172		1.5	0.062		1.0	0.818	
Glycerol	2.4E-02	3.1E-02	2.5E-02	2.5E-02	2.5E-02	1.7E-02	2.0E-02	1.8E-02	2.3E-02	2.9E-02	1.7E-02	2.4E-02	2.7E-02	3.9E-03	2.2E-02	4.4E-03	2.0E-02	2.5E-03	2.3E-02	6.1E-03	0.8	0.285	0.8	0.082		0.9	0.503		1.0	0.824		1.2	0.451	
O-	6.0E-02	6.2E-02	7.4E-02	4.5E-02	6.6E-02	5.3E-02	5.5E-02	5.1E-02	6.0E-02	5.6E-02	6.2E-02	5.7E-02	6.5E-02	7.3E-03	5.5E-02	1.0E-02	5.5E-02	4.4E-03	5.8E-02	3.0E-03	0.8	0.231	0.8	0.119		0.9	0.236		1.1	0.614		1.1	0.345	
Betaine aldehyde_+H_2O	1.9E-03	2.1E-03	2.2E-03	2.0E-03	1.9E-03	1.3E-03	1.6E-03	2.0E-03	1.6E-03	1.7E-03	1.1E-03	1.5E-03	2.0E-03	1.3E-04	1.7E-03	3.6E-04	1.7E-03	2.6E-04	1.5E-03	3.1E-04	0.8	0.250	0.8	0.156		0.7	0.067		0.9	0.410		0.8	0.318	
GTP	1.4E-03	1.7E-03	1.9E-03	8.0E-04	1.5E-03	1.9E-03	1.2E-03	1.0E-03	1.5E-03	3.8E-04	4.0E-04	4.3E-04	1.7E-03	2.7E-04	1.4E-03	5.5E-04	1.2E-03	2.3E-04	4.1E-04	2.4E-05	0.8	0.468	0.7	0.083		0.2	0.014	*	0.3	0.092		0.3	0.025	*
Hypotaurine	6.1E-03	4.5E-03	5.1E-03	2.5E-03	5.9E-03	4.5E-03	5.1E-03	4.0E-03	4.1E-03	6.2E-03	4.6E-03	6.9E-03	5.2E-03	7.8E-04	4.3E-03	1.7E-03	4.4E-03	6.5E-04	5.9E-03	1.2E-03	0.8	0.457	0.8	0.224		1.1	0.467		1.4	0.259		1.3	0.149	
GDP	2.1E-03	2.4E-03	2.4E-03	1.3E-03	2.0E-03	2.4E-03	2.0E-03	1.9E-03	2.2E-03	1.2E-03	1.0E-03	1.2E-03	2.3E-03	1.5E-04	1.9E-03	5.8E-04	2.0E-03	1.5E-04	1.1E-03	7.9E-05	0.8	0.341	0.9	0.061		0.5	0.001	**	0.6	0.142		0.6	0.002	**
ADP-ribose	4.8E-03	4.6E-03	7.7E-03	3.6E-03	7.2E-03	3.3E-03	6.4E-03	8.0E-03	3.6E-03	8.4E-03	7.8E-03	8.8E-03	5.7E-03	1.7E-03	4.7E-03	2.2E-03	6.0E-03	2.2E-03	8.3E-03	4.7E-04	0.8	0.563	1.1	0.864		1.5	0.112		1.8	0.095		1.4	0.204	
Ser	4.8E-02	4.1E-02	3.2E-02	3.6E-02	3.6E-02	2.6E-02	2.7E-02	3.7E-02	3.0E-02	3.1E-02	3.0E-02	3.6E-02	4.0E-02	8.4E-03	3.3E-02	5.6E-03	3.1E-02	5.2E-03	3.2E-02	2.8E-03	0.8	0.265	0.8	0.208		0.8	0.236		1.0	0.949		1.0	0.810	
Ectoine	9.4E-04	7.7E-04	7.4E-04	4.6E-04	8.7E-04	6.3E-04	6.1E-04	5.8E-04	6.9E-04	9.8E-04	7.9E-04	8.1E-04	8.1E-04	1.1E-04	6.5E-04	2.1E-04	6.3E-04	5.8E-05	8.6E-04	1.0E-04	0.8	0.320	0.8	0.073		1.1	0.604		1.3	0.220		1.4	0.040	*
Adenylosuccinic acid	1.3E-03	7.8E-04	6.8E-04	8.2E-04	7.7E-04	5.9E-04	7.4E-04	9.0E-04	9.1E-04	1.1E-03	1.0E-03	1.2E-03	9.1E-04	3.2E-04	7.3E-04	1.2E-04	8.5E-04	9.6E-05	1.1E-03	1.1E-04	0.8	0.426	0.9	0.760		1.2	0.446		1.5	0.019	*	1.3	0.045	*
N,*N*-Dimethylglycine	1.7E-02	1.8E-02	1.7E-02	5.7E-03	1.9E-02	1.5E-02	1.9E-02	1.4E-02	1.6E-02	2.4E-02	1.8E-02	1.8E-02	1.7E-02	5.9E-04	1.3E-02	6.8E-03	1.6E-02	2.7E-03	2.0E-02	3.4E-03	0.8	0.451	1.0	0.694		1.2	0.256		1.5	0.226		1.2	0.208	

S-Adenosylmethionine	6.2E-03	5.3E-03	7.3E-03	4.6E-03	5.7E-03	4.3E-03	5.2E-03	5.1E-03	5.4E-03	5.2E-03	6.7E-03	5.5E-03	6.3E-03	1.0E-03	4.9E-03	7.4E-04	5.2E-03	1.3E-04	5.8E-03	7.6E-04	0.8	0.126		0.8	0.205		0.9	0.525		1.2	0.210		1.1	0.329	
Ophthalmic acid	1.8E-03	1.3E-03	1.4E-03	8.3E-04	1.4E-03	1.2E-03	1.8E-03	1.3E-03	1.4E-03	1.4E-03	1.4E-03	1.3E-03	1.5E-03	2.8E-04	1.1E-03	3.0E-04	1.5E-03	2.7E-04	1.4E-03	7.5E-05	0.8	0.221		1.0	0.914		0.9	0.623		1.2	0.283		0.9	0.522	
Trimethylamine	6.2E-04	9.1E-04	8.6E-04	3.6E-04	8.2E-04	6.6E-04	1.5E-03	9.6E-04	1.3E-03	3.0E-03	3.5E-03	3.3E-03	8.0E-04	1.5E-04	6.1E-04	2.3E-04	1.3E-03	2.9E-04	3.3E-03	2.2E-04	0.8	0.323		1.6	0.092		4.1	1.8E-04	***	5.3	1.4E-04	***	2.6	9.7E-04	***
Pro	1.2E-01	8.7E-02	6.4E-02	5.8E-02	8.2E-02	6.6E-02	7.9E-02	8.1E-02	8.9E-02	9.9E-02	6.3E-02	7.7E-02	9.0E-02	2.8E-02	6.9E-02	1.2E-02	8.3E-02	5.3E-03	8.0E-02	1.8E-02	0.8	0.322		0.9	0.704		0.9	0.618		1.2	0.447		1.0	0.780	
Citrulline	8.4E-03	9.5E-03	8.9E-03	6.5E-03	9.2E-03	4.8E-03	9.8E-03	1.0E-02	7.4E-03	8.9E-03	6.6E-03	7.7E-03	9.0E-03	5.5E-04	6.8E-03	2.2E-03	9.2E-03	1.6E-03	7.7E-03	1.2E-03	0.8	0.229		1.0	0.811		0.9	0.204		1.1	0.574		0.8	0.273	
β-Ala	1.6E-02	1.4E-02	1.1E-02	1.1E-02	1.1E-02	8.2E-03	1.1E-02	1.1E-02	9.2E-03	1.4E-02	9.8E-03	1.1E-02	1.4E-02	2.7E-03	1.0E-02	1.8E-03	1.0E-02	8.5E-04	1.2E-02	2.2E-03	0.8	0.170		0.7	0.150		0.9	0.408		1.1	0.457		1.2	0.352	
Gluconic acid	1.5E-02	1.3E-02	1.7E-02	8.3E-03	1.3E-02	1.4E-02	1.5E-02	1.6E-02	1.9E-02	2.0E-02	1.8E-02	1.7E-02	1.5E-02	1.6E-03	1.2E-02	2.9E-03	1.6E-02	1.8E-03	1.8E-02	1.8E-03	0.8	0.148		1.1	0.415		1.2	0.079		1.6	0.033	*	1.1	0.246	
Choline	5.4E-02	6.2E-02	7.1E-02	5.5E-02	5.7E-02	3.0E-02	5.5E-02	7.6E-02	5.8E-02	5.9E-02	4.4E-02	5.9E-02	6.2E-02	8.9E-03	4.7E-02	1.5E-02	6.3E-02	1.1E-02	5.4E-02	8.4E-03	0.8	0.225		1.0	0.960		0.9	0.305		1.1	0.546		0.9	0.343	
Gluconolactone	6.6E-03	5.9E-03	6.7E-03	3.7E-03	5.5E-03	5.3E-03	6.5E-03	6.5E-03	7.4E-03	8.1E-03	7.2E-03	6.7E-03	6.4E-03	4.5E-04	4.8E-03	9.7E-04	6.8E-03	5.5E-04	7.3E-03	6.9E-04	0.8	0.090		1.1	0.364		1.2	0.125		1.5	0.025	*	1.1	0.350	
UMP	1.9E-02	1.9E-02	2.0E-02	1.1E-02	1.7E-02	1.5E-02	1.9E-02	2.0E-02	1.9E-02	2.5E-02	2.5E-02	2.6E-02	1.9E-02	4.0E-04	1.4E-02	2.8E-03	1.9E-02	4.8E-04	2.5E-02	8.3E-04	0.8	0.100		1.0	0.962		1.3	0.002	**	1.8	0.015	*	1.3	0.001	**
3-Ureidopropionic	4.9E-04	6.0E-04	2.5E-04	3.7E-04	2.9E-04	3.6E-04	6.1E-04	4.4E-04	5.2E-04	5.7E-04	2.8E-04	4.0E-04	4.5E-04	1.8E-04	3.4E-04	4.2E-05	5.3E-04	8.5E-05	4.2E-04	1.5E-04	0.8	0.397		1.2	0.552		0.9	0.829		1.2	0.456		0.8	0.351	
Malic acid	5.2E-02	3.4E-02	3.1E-02	3.2E-02	2.9E-02	2.8E-02	2.4E-02	2.2E-02	2.2E-02	2.6E-02	2.2E-02	2.3E-02	3.9E-02	1.1E-02	2.9E-02	2.3E-03	2.3E-02	1.2E-03	2.4E-02	1.9E-03	0.8	0.265		0.6	0.124		0.6	0.134		0.8	0.032	*	1.0	0.556	
Glu	2.1E-01	9.6E-02	1.0E-01	1.1E-01	9.5E-02	1.0E-01	9.2E-02	1.1E-01	1.1E-01	1.3E-01	1.2E-01	1.0E-01	1.4E-01	6.3E-02	1.0E-01	5.7E-03	1.1E-01	1.2E-02	1.2E-01	1.5E-02	0.7	0.438		0.8	0.502		0.8	0.633		1.1	0.238		1.1	0.466	
Pantothenic acid	3.5E-04	3.5E-04	3.9E-04	2.9E-04	2.5E-04	2.6E-04	3.0E-04	2.7E-04	2.9E-04	3.3E-04	3.2E-04	2.8E-04	3.6E-04	2.6E-05	2.7E-04	1.9E-05	2.9E-04	1.4E-05	3.1E-04	2.5E-05	0.7	0.010	*	0.8	0.022	*	0.9	0.081		1.2	0.081		1.1	0.225	
Isobutyryl CoA_divalent	8.5E-05	5.0E-05	1.5E-04	5.7E-05	7.8E-05	7.7E-05	1.4E-04	4.3E-04	2.8E-04	1.9E-04	2.5E-04	1.2E-04	9.5E-05	5.1E-05	7.1E-05	1.2E-05	2.8E-04	1.5E-04	1.9E-04	6.6E-05	0.7	0.498		3.0	0.144		2.0	0.132		2.7	0.086		0.7	0.379	
Homocitrulline	2.3E-04	2.0E-04	1.8E-04	1.5E-04	1.8E-04	1.3E-04	2.4E-04	2.3E-04	2.1E-04	1.5E-04	1.8E-04	1.3E-04	2.0E-04	2.2E-05	1.5E-04	2.5E-05	2.2E-04	1.3E-05	1.5E-04	2.7E-05	0.7	0.053		1.1	0.238		0.8	0.070		1.0	0.901		0.7	0.028	*
Val	1.3E-01	1.1E-01	1.1E-01	7.8E-02	9.6E-02	7.8E-02	1.1E-01	1.1E-01	1.2E-01	1.1E-01	1.1E-01	1.1E-01	1.1E-01	1.1E-02	8.4E-02	1.0E-02	1.1E-01	3.3E-03	1.1E-01	3.9E-03	0.7	0.025	*	1.0	0.886		1.0	0.476		1.3	0.038	*	1.0	0.203	
IMP	3.9E-03	5.0E-03	4.5E-03	4.1E-03	2.8E-03	3.0E-03	5.0E-03	5.3E-03	4.9E-03	3.5E-03	2.5E-03	2.4E-03	4.5E-03	5.5E-04	3.3E-03	7.2E-04	5.1E-03	1.7E-04	2.8E-03	6.2E-04	0.7	0.090		1.1	0.218		0.6	0.025	*	0.8	0.408		0.6	0.019	*
Isovalerylcarnitin	2.6E-03	2.1E-03	2.2E-03	4.1E-04	2.9E-03	1.8E-03	3.7E-03	3.3E-03	4.4E-03	3.2E-03	2.5E-03	2.7E-03	2.3E-03	2.9E-04	1.7E-03	1.2E-03	3.8E-03	5.6E-04	2.8E-03	3.8E-04	0.7	0.463		1.6	0.028	*	1.2	0.160		1.7	0.249		0.7	0.076	
SDMA	1.8E-04	1.5E-04	1.4E-04	1.0E-04	1.4E-04	9.6E-05	1.2E-04	1.5E-04	1.5E-04	1.3E-04	1.2E-04	1.4E-04	1.6E-04	2.0E-05	1.1E-04	2.3E-05	1.4E-04	1.6E-05	1.3E-04	1.1E-05	0.7	0.072		0.9	0.391		0.9	0.190		1.2	0.248		0.9	0.552	
N-Acetylhistidine	9.5E-04	7.6E-04	9.0E-04	5.5E-04	6.5E-04	6.6E-04	8.4E-04	7.5E-04	6.8E-04	7.8E-04	6.2E-04	7.0E-04	8.7E-04	9.9E-05	6.2E-04	6.1E-05	7.6E-04	7.6E-05	7.0E-04	7.8E-05	0.7	0.029	*	0.9	0.208		0.8	0.089		1.1	0.235		0.9	0.422	
N-Acetylserine	3.7E-04	3.3E-04	2.8E-04	2.1E-04	2.8E-04	2.2E-04	2.7E-04	3.0E-04	2.5E-04	3.1E-04	2.5E-04	2.7E-04	3.3E-04	4.7E-05	2.3E-04	3.9E-05	2.7E-04	2.4E-05	2.8E-04	3.4E-05	0.7	0.059		0.8	0.173		0.8	0.223		1.2	0.214		1.0	0.839	
N-	3.9E-04	4.5E-04	5.4E-04	2.2E-04	3.9E-04	3.5E-04	5.3E-04	4.1E-04	5.2E-04	4.4E-04	5.4E-04	4.9E-04	4.6E-04	7.1E-05	3.2E-04	8.9E-05	4.9E-04	6.8E-05	4.9E-04	5.1E-05	0.7	0.109		1.1	0.681		1.1	0.615		1.5	0.064		1.0	0.959	
Met	1.4E-02	1.1E-02	1.1E-02	9.7E-03	8.2E-03	7.3E-03	1.2E-02	1.2E-02	1.1E-02	1.3E-02	1.1E-02	9.3E-03	1.2E-02	1.6E-03	8.4E-03	1.2E-03	1.2E-02	7.7E-04	1.1E-02	2.1E-03	0.7	0.036	*	1.0	0.770		0.9	0.602		1.3	0.128		1.0	0.710	
Isovalerylalanine	4.2E-04	3.3E-04	2.3E-04	1.9E-04	2.7E-04	2.1E-04	3.3E-04	3.0E-04	3.8E-04	4.1E-04	2.4E-04	3.0E-04	3.3E-04	9.4E-05	2.2E-04	4.0E-05	3.4E-04	4.3E-05	3.2E-04	8.9E-05	0.7	0.195		1.0	0.869		1.0	0.915		1.4	0.207		0.9	0.759	
N^5-	3.8E-04	3.7E-04	5.3E-04	2.4E-04	3.3E-04	3.0E-04	5.8E-04	5.5E-04	5.2E-04	4.5E-04	4.8E-04	4.1E-04	4.3E-04	8.8E-05	2.9E-04	4.6E-05	5.5E-04	3.0E-05	4.5E-04	3.6E-05	0.7	0.100		1.3	0.120		1.1	0.718		1.5	0.011	*	0.8	0.019	*
GABA	4.2E-03	3.2E-03	4.0E-03	2.0E-03	3.1E-03	2.5E-03	3.7E-03	3.8E-03	3.7E-03	4.5E-03	3.9E-03	3.8E-03	3.8E-03	5.4E-04	2.6E-03	5.4E-04	3.7E-03	6.1E-05	4.0E-03	3.8E-04	0.7	0.049	*	1.0	0.824		1.1	0.558		1.6	0.022	*	1.1	0.275	
Imidazole-4-acetic acid	1.7E-04	1.7E-04	1.9E-04	8.5E-05	1.6E-04	1.2E-04	1.3E-04	1.3E-04	1.7E-04	1.7E-04	1.7E-04	2.1E-04	1.8E-04	1.3E-05	1.2E-04	3.9E-05	1.4E-04	2.7E-05	1.8E-04	2.0E-05	0.7	0.111		0.8	0.136		1.0	0.808		1.5	0.093		1.3	0.121	
Cystathionine	1.2E-02	7.1E-03	5.8E-03	3.7E-03	6.9E-03	6.1E-03	6.6E-03	7.5E-03	8.5E-03	1.0E-02	6.4E-03	7.6E-03	8.3E-03	3.2E-03	5.6E-03	1.7E-03	7.5E-03	9.3E-04	8.2E-03	2.1E-03	0.7	0.286		0.9	0.732		1.0	0.970		1.5	0.173		1.1	0.660	
Glucuronic acid	4.9E-03	5.4E-03	5.6E-03	2.7E-03	4.5E-03	3.5E-03	6.1E-03	6.0E-03	5.6E-03	5.7E-03	5.8E-03	6.1E-03	5.3E-03	3.7E-04	3.6E-03	9.3E-04	5.9E-03	2.6E-04	5.8E-03	2.0E-04	0.7	0.069		1.1	0.102		1.1	0.112		1.6	0.046	*	1.0	0.868	
N-Acetyllysine	2.0E-03	1.9E-03	2.3E-03	1.0E-03	1.8E-03	1.4E-03	2.1E-03	2.1E-03	2.1E-03	2.0E-03	1.8E-03	1.9E-03	2.1E-03	2.4E-04	1.4E-03	3.8E-04	2.1E-03	2.5E-05	1.9E-03	7.1E-05	0.7	0.069		1.0	0.883		0.9	0.380		1.4	0.135		0.9	0.036	*
Galacturonic	5.1E-04	3.8E-04	4.1E-04	2.1E-04	3.5E-04	3.1E-04	5.2E-04	5.1E-04	4.6E-04	4.5E-04	4.3E-04	4.9E-04	4.3E-04	7.0E-05	2.9E-04	7.2E-05	5.0E-04	3.6E-05	4.6E-04	3.0E-05	0.7	0.068		1.1	0.254		1.1	0.611		1.6	0.041	*	0.9	0.227	
2-Hydroxybutyric acid	2.9E-03	2.0E-03	2.4E-03	9.9E-04	1.9E-03	2.0E-03	2.6E-03	2.8E-03	3.7E-03	3.3E-03	2.9E-03	2.6E-03	2.4E-03	4.8E-04	1.6E-03	5.6E-04	3.0E-03	5.7E-04	3.0E-03	3.6E-04	0.7	0.129		1.2	0.234		1.2	0.212		1.8	0.033	*	1.0	0.834	
Glutaric acid	7.7E-04	5.1E-04	3.8E-04	1.8E-04	4.4E-04	4.8E-04	7.2E-04	6.0E-04	4.4E-04	7.6E-04	4.8E-04	5.6E-04	5.6E-04	2.0E-04	3.7E-04	1.7E-04	5.9E-04	1.4E-04	6.0E-04	1.5E-04	0.7	0.282		1.1	0.840		1.1	0.776		1.6	0.148		1.0	0.914	
Octanoylcarnitin	2.2E-04	8.9E-05	1.6E-04	N.D.	1.3E-04	7.9E-05	7.8E-04	1.0E-03	8.3E-04	1.2E-04	1.1E-04	1.2E-04	1.6E-04	6.5E-05	1.0E-04	3.3E-05	8.8E-04	1.4E-04	1.2E-04	8.2E-06	0.7	0.318		5.7	0.004	**	0.8	0.421		1.1	0.630		0.13	0.010	*
γ-Glu-Cys	8.4E-04	4.6E-04	6.9E-04	4.5E-04	4.1E-04	4.3E-04	6.5E-04	6.9E-04	6.8E-04	7.7E-04	6.1E-04	5.7E-04	6.7E-04	1.9E-04	4.3E-04	2.0E-05	6.8E-04	2.0E-05	6.5E-04	1.1E-04	0.7	0.165		1.0	0.934		1.0	0.904		1.5	0.068		1.0	0.712	
Urea	5.6E-01	5.5E-01	5.5E-01	3.3E-01	4.2E-01	3.2E-01	5.4E-01	5.3E-01	5.2E-01	4.9E-01	5.2E-01	4.8E-01	5.5E-01	4.7E-03	3.6E-01	5.8E-02	5.3E-01	1.3E-02	5.0E-01	2.2E-02	0.6	0.028	*	1.0	0.079		0.9	0.051		1.4	0.037	*	0.9	0.131	
Acetoacetamide	1.9E-04	1.9E-04	2.1E-04	N.D.	1.3E-04	N.D.	1.6E-04	1.6E-04	1.5E-04	1.8E-04	1.8E-04	1.5E-04	1.9E-04	1.3E-05	1.3E-04	N.A.	1.6E-04	7.2E-06	1.7E-04	1.9E-05	0.6	N.A.		0.8	0.017	*	0.9	0.120		1.3	N.A.		1.1	0.367	
Ornithine	9.2E-02	8.9E-02	9.2E-02	4.3E-02	7.9E-02	5.4E-02	7.4E-02	7.5E-02	6.7E-02	8.9E-02	8.5E-02	8.6E-02	9.1E-02	1.6E-03	5.8E-02	1.8E-02	7.2E-02	4.7E-03	8.7E-02	2.2E-03	0.6	0.089		0.8	0.012	*	1.0	0.063		1.5	0.112		1.2	0.018	*
3-Aminoisobutyric	2.6E-03	1.9E-03	1.6E-03	1.2E-03	1.6E-03	1.0E-03	1.5E-03	1.7E-03	1.4E-03	1.7E-03	1.6E-03	1.7E-03	2.0E-03	5.3E-04	1.3E-03	2.9E-04	1.6E-03	1.4E-04	1.6E-03	5.6E-05	0.6	0.124		0.8	0.278		0.8	0.350		1.3	0.163		1.0	0.445	
Isoglutamic acid	1.1E-03	7.9E-04	1.4E-03	6.7E-04	7.9E-04	6.4E-04	9.0E-04	8.9E-04	9.9E-04	8.9E-04	1.1E-03	6.5E-04	1.1E-03	3.0E-04	7.0E-04	8.2E-05	9.3E-04	5.7E-05	8.9E-04	2.4E-04	0.6	0.140		0.8	0.434		0.8	0.419		1.3	0.299		1.0	0.836	
XC0061	6.5E-03	6.3E-03	7.2E-03	2.5E-03	5.3E-03	4.9E-03	6.6E-03	7.2E-03	7.4E-03	7.8E-03	6.4E-03	5.1E-03	6.7E-03	4.4E-04	4.2E-03	1.5E-03	7.0E-03	4.2E-04	6.4E-03	1.3E-03	0.6	0.103		1.1	0.346		1.0	0.787		1.5	0.141		0.9	0.516	
S-Adenosylhomocysteine	4.9E-03	3.9E-03	3.0E-03	2.5E-03	2.7E-03	2.2E-03	4.2E-03	4.6E-03	4.3E-03	4.5E-03	3.2E-03	3.4E-03	3.9E-03	9.5E-04	2.5E-03	2.6E-04	4.4E-03	1.8E-04	3.7E-03	7.0E-04	0.6	0.112		1.1	0.503		0.9	0.779		1.5	0.079		0.9	0.243	
4-Guanidinobutyric	1.5E-02	1.3E-02	1.7E-02	5.8E-03	1.3E-02	9.8E-03	1.5E-02	1.4E-02	1.6E-02	2.0E-02	1.9E-02	1.9E-02	1.5E-02	1.9E-03	9.7E-03	3.8E-03	1.5E-02	6.0E-04	1.9E-02	7.6E-04	0.6	0.107		1.0	0.868		1.3	0.048	*	2.0	0.043	*	1.3	0.002	**
5'-Deoxy-5'-methylthioadenosine	2.7E-04	1.9E-04	1.7E-04	1.2E-04	1.5E-04	1.3E-04	1.9E-04	1.6E-04	1.6E-04	1.4E-04	1.6E-04	1.4E-04	2.1E-04	5.4E-05	1.3E-04	1.9E-05	1.7E-04	1.6E-05	1.5E-04	1.0E-05	0.6	0.115		0.8	0.300		0.7	0.161		1.1	0.400		0.9	0.112	
Sedoheptulose 7-phosphate	7.5E-03	5.4E-03	5.3E-03	4.0E-03	3.5E-03	3.8E-03	6.0E-03	6.1E-03	6.8E-03	2.3E-03	2.2E-03	2.2E-03	6.0E-03	1.2E-03	3.8E-03	2.9E-04	6.3E-03	4.4E-04	2.2E-03	3.3E-05	0.6	0.080		1.0	0.747		0.4	0.034	*	0.6	0.011	*	0.4	0.004	**
Arg	1.4E-04	2.0E-04	1.9E-04	1.0E-04	1.4E-04	8.9E-05	2.4E-04	6.0E-04	2.4E-04	2.2E-04	1.9E-04	1.9E-04	1.8E-04	3.2E-05	1.1E-04	2.6E-05	3.6E-04	2.1E-04	2.0E-04	1.6E-05	0.6	0.052		2.0	0.267		1.1	0.382		1.8	0.013	*	0.5	0.312	
1-Pyrroline 5-carboxylic acid	9.1E-04	5.2E-04	6.5E-04	5.0E-04	3.8E-04	3.9E-04	5.2E-04	5.4E-04	7.0E-04	6.3E-04	6.4E-04	5.4E-04	6.9E-04	2.0E-04	4.2E-04	6.4E-05	5.9E-04	1.0E-04	6.1E-04	5.9E-05	0.6	0.133		0.9	0.489		0.9	0.543		1.4	0.022	*	1.0	0.820	
Homoserine	4.1E-04	3.1E-04	3.5E-04	1.5E-04	2.6E-04	2.4E-04	3.6E-04	3.3E-04	3.3E-04	2.9E-04	3.0E-04	2.6E-04	3.6E-04	5.2E-05	2.2E-04	5.8E-05	3.4E-04	1.7E-05	2.8E-04	2.2E-05	0.6	0.037	*	0.9	0.619		0.8	0.121		1.3	0.175		0.8	0.031	*
cis-4-Hydroxyproline	3.7E-04	2.4E-04	2.6E-04	1.9E-04	1.7E-04	1.7E-04	2.6E-04	2.5E-04	2.8E-04	2.9E-04	2.8E-04	2.3E-04	2.9E-04	7.1E-05	1.8E-04	9.0E-06	2.6E-04	1.6E-05	2.7E-04	3.5E-05	0.6	0.102		0.9	0.537		0.9	0.605		1.5	0.040	*	1.0	0.867	
XA0027	4.2E-04	2.4E-04	N.D.	N.D.	2.0E-04	N.D.	N.D.	N.D.	N.D.	N.D.	N.D.	N.D.	3.3E-04	1.3E-04	2.0E-04	N.A.	N.D.	N.A.	N.D.	N.A.	0.6	N.A.		<1	N.A.		<1	N.A.		<1	N.A.		N.A.	N.A.	
Sarcosine	3.4E-02	2.5E-02	2.2E-02	9.5E-03	2.1E-02	1.7E-02	3.1E-02	2.5E-02	3.1E-02	3.7E-02	2.6E-02	2.9E-02	2.7E-02	6.1E-03	1.6E-02	5.8E-03	2.9E-02	3.3E-03	3.1E-02	6.0E-03	0.6	0.085		1.1	0.596		1.1	0.511		1.9	0.038	*	1.0	0.782	
XA0012	1.2E-03	1.0E-03	1.0E-03	5.5E-04	7.8E-04	6.1E-04	1.0E-03	1.1E-03	1.0E-03	9.0E-04	6.5E-04	7.2E-04	1.1E-03	1.3E-04	6.5E-04	1.2E-04	1.1E-03	5.9E-05	7.5E-04	1.3E-04	0.6	0.010	*	1.0	0.614		0.7	0.030	*	1.2	0.355		0.7	0.042	*
2-Methylserine	3.1E-04	2.3E-04	2.7E-04	N.D.	1.9E-04	1.2E-04	2.8E-04	3.4E-04	3.3E-04	2.3E-04	2.2E-04	1.7E-04	2.7E-04	3.9E-05	1.6E-04	5.0E-05	3.2E-04	3.4E-05	2.0E-04	3.2E-05	0.6	0.127		1.2	0.204		0.8	0.088		1.3	0.385		0.6	0.015	*
Lys	1.6E-01	1.4E-01	1.6E-01	8.6E-02	9.6E-02	8.2E-02	1.2E-01	1.2E-01	1.3E-01	1.4E-01	1.2E-01	1.2E-01	1.5E-01	1.4E-02	8.8E-02	7.1E-03	1.3E-01	7.5E-03	1.2E-01	9.2E-03	0.6	0.006	**	0.8	0.066		0.8	0.055		1.4	0.007	**	1.0	0.797	
Hydroxyproline	1.5E-02	8.8E-03	9.4E-03	4.8E-03	8.1E-03	6.0E-03	1.1E-02	8.4E-03	1.2E-02	1.2E-02	9.8E-03	9.8E-03	1.1E-02	3.3E-03	6.3E-03	1.7E-03	1.0E-02	1.7E-03	1.1E-02	1.3E-03	0.6	0.115		0.9	0.807		1.0	0.838		1.7	0.028	*	1.0	0.932	
XA0033	3.7E-02	3.4E-02	3.5E-02	1.1E-02	2.6E-02	2.2E-02	2.5E-02	2.7E-02	3.1E-02	3.6E-02	2.6E-02	2.7E-02	3.5E-02	1.4E-03	2.0E-02	7.8E-03	2.7E-02	2.9E-03	3.0E-02	5.5E-03	0.6	0.072		0.8	0.028	*	0.8	0.229		1.5	0.154		1.1	0.569	
Argininosuccinic acid	1.4E-03	1.4E-03	1.8E-03	7.6E-04	1.1E-03	7.1E-04	1.5E-03	1.7E-03	1.2E-03	6.4E-04	6.1E-04	6.0E-04	1.5E-03	2.0E-04	8.5E-04	2.0E-04	1.5E-03	2.7E-04	6.2E-04	1.8E-05	0.6	0.014	*	0.9	0.705		0.4	0.015	*	0.7	0.178		0.4	0.031	*
Trigonelline	5.6E-03	5.0E-03	4.6E-03	1.8E-03	4.0E-03	2.5E-03	4.8E-03	4.2E-03	5.1E-03	6.3E-03	5.2E-03	5.8E-03	5.1E-03	5.2E-04	2.8E-03	1.2E-03	4.7E-03	5.0E-04	5.8E-03	5.4E-04	0.5	0.058		0.9	0.414		1.1	0.176		2.1	0.030	*	1.2	0.063	
3-Hydroxybutyric acid	1.8E-02	1.5E-02	1.4E-02	8.3E-03	8.8E-03	8.4E-03	1.6E-02	1.7E-02	1.5E-02	1.1E-02	9.9E-03	1.0E-02	1.6E-02	2.4E-03	8.5E-03	2.7E-04	1.6E-02	9.7E-04	1.0E-02	5.3E-04	0.5	0.035	*	1.0	0.798		0.7	0.058		1.2	0.013	*	0.6	0.003	**
Phosphorylcholi	2.1E-01	1.9E-01	1.9E-01	5.5E-02	1.4E-01	1.1E-01	1.4E-01	1.4E-01	1.7E-01	1.9E-01	1.4E-01	1.4E-01	1.9E-01	1.0E-02	1.0E-01	4.6E-02	1.5E-01	1.8E-02	1.6E-01	3.3E-02	0.5	0.069		0.8	0.026	*	0.8	0.176		1.5	0.187		1.1	0.714	
Methionine sulfoxide	1.8E-03	1.1E-03	1.5E-03	6.8E-04	8.8E-04	7.6E-04	1.7E-03	1.8E-03	1.4E-03	1.3E-03	1.6E-03	1.1E-03	1.4E-03	3.6E-04	7.7E-04	1.0E-04	1.6E-03	2.1E-04	1.4E-03	2.6E-04	0.5	0.073		1.1	0.440		1.0	0.816		1.8	0.043	*	0.8	0.235	
3-Methylhistidine	2.8E-03	2.0E-03	2.5E-03	1.2E-03	1.5E-03	1.2E-03	2.4E-03	2.0E-03	2.9E-03	1.8E-03	1.9E-03	1.6E-03	2.4E-03	3.9E-04	1.3E-03	1.5E-04	2.4E-03	4.1E-04	1.8E-03	1.2E-04	0.5	0.027	*	1.0	0.937		0.7	0.097		1.4	0.014	*	0.7	0.096	
Imidazolelactic acid	3.2E-04	3.5E-04	3.4E-04	9.8E-05	2.6E-04	1.8E-04	2.7E-04	2.0E-04	3.5E-04	3.0E-04	2.6E-04	3.4E-04	3.4E-04	1.6E-05	1.8E-04	7.9E-05	2.7E-04	7.6E-05	3.0E-04	3.5E-05	0.5	0.067		0.8	0.268		0.9	0.216		1.7	0.095		1.1	0.578	
N^6-Acetyllysine	4.9E-04	5.2E-04	4.8E-04	2.0E-04	3.2E-04	2.7E-04	4.7E-04	4.0E-04	4.2E-04	4.1E-04	3.5E-04	3.7E-04	5.0E-04	2.1E-05	2.6E-04	6.0E-05	4.3E-04	3.4E-05	3.8E-04	3.0E-05	0.5	0.013	*	0.9	0.050		0.8	0.006	**	1.4	0.062		0.9	0.106	
Pipecolic acid	3.6E-03	2.5E-03	2.9E-03	1.4E-03	1.9E-03	1.4E-03	2.7E-03	2.3E-03	2.9E-03	2.9E-03	2.4E-03	2.0E-03	3.0E-03	5.6E-04	1.6E-03	2.9E-04	2.6E-03	2.8E-04	2.4E-03	4.5E-04	0.5	0.030	*	0.9	0.414		0.8	0.245		1.5	0.060		0.9	0.509	
Trimethylamine *N*-oxide	7.1E-03	8.9E-03	7.3E-03	2.7E-03	5.0E-03	4.5E-03	1.7E-02	6.1E-03	7.3E-03	1.5E-02	1.2E-02	1.5E-02	7.8E-03	9.9E-04	4.1E-03	1.2E-03	1.0E-02	5.7E-03	1.4E-02	1.5E-03	0.5	0.016	*	1.3	0.576		1.8	0.006	**	3.5	0.001	**	1.4	0.334	
2-Aminobutyric acid	1.0E-02	5.5E-03	6.1E-03	2.9E-03	4.6E-03	4.0E-03	6.6E-03	6.1E-03	7.6E-03	6.9E-03	6.0E-03	5.2E-03	7.3E-03	2.6E-03	3.8E-03	8.5E-04	6.8E-03	7.2E-04	6.0E-03	8.5E-04	0.5	0.136		0.9	0.765		0.8	0.482		1.6	0.035	*	0.9	0.289	
γ-Butyrobetaine	6.2E-03	6.8E-03	8.0E-03	2.7E-03	4.5E-03	3.3E-03	6.3E-03	5.6E-03	5.8E-03	5.8E-03	5.1E-03	4.9E-03	7.0E-03	9.0E-04	3.5E-03	9.4E-04	5.9E-03	3.5E-04	5.3E-03	5.0E-04	0.5	0.010	**	0.8	0.162		0.8	0.062		1.5	0.060		0.9	0.169	
Stachydrine	3.0E-02	2.8E-02	3.0E-02	1.1E-02	2.1E-02	1.2E-02	3.1E-02	2.9E-02	2.8E-02	2.8E-02	3.3E-02	2.6E-02	2.9E-02	9.3E-04	1.5E-02	5.5E-03	2.9E-02	1.5E-03	2.9E-02	3.4E-03	0.5	0.039	*	1.0	0.859		1.0	0.825		2.0	0.025	*	1.0	0.896	
1-Methylnicotinami	7.7E-04	5.2E-04	5.5E-04	3.7E-04	2.4E-04	2.9E-04	5.5E-04	6.4E-04	5.1E-04	5.9E-04	3.9E-04	3.8E-04	6.1E-04	1.4E-04	3.0E-04	7.0E-05	5.7E-04	6.4E-05	4.5E-04	1.2E-04	0.5	0.038	*	0.9	0.658		0.7	0.202		1.5	0.135		0.8	0.226	
Saccharopine	2.2E-03	1.2E-03	9.9E-04	2.4E-04	8.3E-04	1.1E-03	1.5E-03	6.6E-04	6.8E-04	1.9E-03	9.9E-04	8.4E-04	1.5E-03	6.7E-04	7.2E-04	4.4E-04	9.3E-04	4.6E-04	1.3E-03	5.9E-04	0.5	0.188		0.6	0.318		0.8	0.681		1.7	0.282		1.3	0.503	
Betaine	7.1E-01	6.6E-01	8.2E-01	2.7E-01	4.8E-01	2.9E-01	8.0E-01	6.4E-01	6.0E-01	5.8E-01	6.8E-01	6.1E-01	7.3E-01	8.2E-02	3.5E-01	1.2E-01	6.8E-01	1.0E-01	6.3E-01	5.0E-02	0.5	0.013	*	0.9	0.539		0.9	0.143		1.8	0.040	*	0.9	0.469	
N-Acetylglutamic	7.7E-03	5.7E-03	5.8E-03	2.2E-03	3.5E-03	3.0E-03	4.4E-03	4.7E-03	5.0E-03	4.6E-03	4.0E-03	4.1E-03	6.4E-03	1.1E-03	2.9E-03	6.7E-04	4.7E-03	3.0E-04	4.2E-03	3.3E-04	0.5	0.017	*	0.7	0.113		0.7	0.071		1.4	0.059		0.9	0.155	
4-Acetamidobutanoic acid	5.6E-04	2.7E-04	3.7E-04	3.8E-05	3.1E-04	1.9E-04	1.3E-03	2.4E-03	1.6E-03	6.8E-04	5.4E-04	6.1E-04	4.0E-04	1.5E-04	1.8E-04	1.4E-04	1.8E-03	5.7E-04	6.1E-04	6.9E-05	0.5	0.139		4.4	0.046	*	1.5	0.112		3.4	0.017	*	0.3	0.072	

Butyrylcarnitine	8.7E-03	3.4E-03	6.9E-03	8.6E-04	4.4E-03	3.2E-03	2.0E-02	2.7E-02	2.1E-02	7.1E-03	8.6E-03	5.9E-03	6.3E-03	2.7E-03	2.8E-03	1.8E-03	2.3E-02	3.6E-03	7.2E-03	1.3E-03	0.4	0.140		3.6	0.004	**	1.1	0.645		2.6	0.030	*	0.3	0.010	**
5-Aminovaleric acid	1.2E-02	1.1E-02	1.4E-02	3.8E-03	7.6E-03	4.6E-03	1.2E-02	1.1E-02	1.3E-02	8.3E-03	8.9E-03	8.6E-03	1.2E-02	1.6E-03	5.4E-03	2.0E-03	1.2E-02	6.8E-04	8.6E-03	2.9E-04	0.4	0.011	*	1.0	0.696		0.7	0.054		1.6	0.104		0.7	0.007	**
2-Aminoadipic	6.2E-03	2.6E-03	1.6E-03	1.2E-03	1.5E-03	1.5E-03	2.3E-03	2.1E-03	1.7E-03	3.0E-03	2.3E-03	1.8E-03	3.5E-03	2.4E-03	1.4E-03	1.7E-04	2.1E-03	3.2E-04	2.4E-03	5.9E-04	0.4	0.275		0.6	0.417		0.7	0.516		1.7	0.097		1.2	0.479	
XC0040	6.1E-04	7.4E-04	8.0E-04	1.6E-04	4.2E-04	2.2E-04	7.4E-04	7.3E-04	5.6E-04	4.8E-04	5.4E-04	6.8E-04	7.2E-04	9.7E-05	2.7E-04	1.3E-04	6.7E-04	9.9E-05	5.7E-04	1.0E-04	0.4	0.011	*	0.9	0.601		0.8	0.138		2.1	0.039	*	0.8	0.268	
N^6-Methyllysine	5.9E-04	3.9E-04	3.9E-04	1.9E-04	1.7E-04	1.3E-04	5.1E-04	4.7E-04	4.9E-04	2.0E-04	2.9E-04	2.3E-04	4.6E-04	1.1E-04	1.6E-04	2.7E-05	4.9E-04	2.3E-05	2.4E-04	4.7E-05	0.4	0.039	*	1.1	0.657		0.5	0.063		1.5	0.085		0.5	0.004	**
1*H*-Imidazole-4-propionic acid	3.0E-04	1.9E-04	3.5E-04	7.0E-05	1.5E-04	6.7E-05	4.3E-04	2.0E-04	2.4E-04	9.1E-05	9.3E-05	8.3E-05	2.8E-04	8.1E-05	9.7E-05	4.9E-05	2.9E-04	1.2E-04	8.9E-05	5.0E-06	0.3	0.036	*	1.0	0.933		0.3	0.053		0.9	0.804		0.3	0.102	
Betonicine	5.5E-04	4.7E-04	6.7E-04	1.3E-04	2.8E-04	1.6E-04	5.7E-04	6.1E-04	5.1E-04	3.8E-04	5.6E-04	4.2E-04	5.6E-04	9.7E-05	1.9E-04	7.6E-05	5.6E-04	5.5E-05	4.5E-04	9.4E-05	0.3	0.007	**	1.0	0.993		0.8	0.234		2.4	0.021	*	0.8	0.176	
Ethyl	1.7E-03	8.3E-04	2.8E-03	7.9E-04	4.8E-04	2.0E-04	5.4E-04	1.5E-03	1.4E-03	1.3E-03	4.8E-03	3.2E-03	1.8E-03	1.0E-03	4.9E-04	3.0E-04	1.2E-03	5.4E-04	3.1E-03	1.8E-03	0.3	0.149		0.7	0.420		1.8	0.337		6.3	0.122		2.7	0.192	
UDP-glucuronic acid	4.8E-03	4.4E-03	5.9E-03	3.5E-04	2.0E-03	6.6E-04	4.3E-03	3.7E-03	2.7E-03	1.8E-03	3.2E-03	3.5E-03	5.0E-03	7.7E-04	1.0E-03	8.8E-04	3.6E-03	8.1E-04	2.8E-03	8.9E-04	0.2	0.004	**	0.7	0.092		0.6	0.034	*	2.8	0.064		0.8	0.343	
Putrescine	1.6E-03	1.6E-03	2.3E-03	1.7E-04	1.9E-04	1.0E-04	1.4E-03	1.4E-03	8.8E-04	1.5E-04	6.2E-04	3.3E-04	1.8E-03	3.8E-04	1.5E-04	4.6E-05	1.2E-03	3.1E-04	3.7E-04	2.3E-04	0.09	0.016	*	0.7	0.111		0.2	0.009	**	2.4	0.251		0.3	0.021	*
N-Methylalanine	2.3E-04	1.6E-04	1.4E-04	N.D.	N.D.	N.D.	N.D.	1.1E-04	N.D.	1.4E-04	1.4E-04	N.D.	1.8E-04	4.5E-05	N.D.	N.A.	1.1E-04	N.A.	1.4E-04	1.4E-06	<1	N.A.		0.6	N.A.		0.8	0.324		1<	N.A.		1.3	N.A.	
Butyric acid	N.D.	N.D.	N.D.	N.D.	N.D.	N.D.	3.1E-04	6.4E-04	3.5E-04	N.D.	2.9E-04	N.D.	N.D.	N.A.	N.D.	N.A.	4.4E-04	1.8E-04	2.9E-04	N.A.	N.A.	N.A.		1<	N.A.		1<	N.A.		1<	N.A.		0.7	N.A.	
Cytosine	N.D.	N.D.	N.D.	N.D.	N.D.	N.D.	4.3E-05	5.7E-05	8.7E-05	N.D.	N.D.	N.D.	N.D.	N.A.	N.D.	N.A.	6.2E-05	2.2E-05	N.D.	N.A.	N.A.	N.A.		1<	N.A.		N.A.	N.A.		N.A.	N.A.		<1	N.A.	

N.D.: Not Detected.

N.A.: Not Available.

¶ in the case of A vs B, the ratio of the average was calculated as A/B.

|| Welch's *t*-test , *p*-value (*<0.05, **<0.01, ***<0.001)

Description of XA･･･or XC･･･ in compound name indicates unknown peaks, which has been detected in some other organism samples in the HMT database for metabolite identification.
