# Supplementary material for: Chronic Intake of Japanese Sake Mediates Radiation-Induced Metabolic Alterations in Mouse Liver
Source: PLoS One. 2016 Jan 11;11(1):e0146730. doi: 10.1371/journal.pone.0146730 (PMC4713437; doi:10.1371/journal.pone.0146730)
Supplement: S4 Table — Values represent the relative areas of each metabolite peak to the peak area of an internal standard. (PDF) [file pone.0146730.s007.pdf]

S4 Table.Metabolome data for the livers of irradiated mice administered 15% ethanol. Values represent the relative areas of each metabolite peak to the peak area of an internal standard.

Compound name	Relative Area																				Comparative Analysis														
	control			rad			ethanol			ethanol+rad			control		rad		ethanol		ethanol+rad		rad vs control			ethanol vs control			ethanol+rad vs control			ethanol+rad vs rad			ethanol+rad vs ethanol		
	1	2	3	4	5	6	7	8	9	10	11	12	Mean	S.D.	Mean	S.D.	Mean	S.D.	Mean	S.D.	Ratio ¶	*p*-value ‖		Ratio ¶	*p*-value ‖		Ratio ¶	*p*-value ‖		Ratio ¶	*p*-value ‖		Ratio ¶	*p*-value ‖	
UDP-glucose UDP-galactose	1.2E-02	9.7E-03	1.5E-02	2.5E-02	2.6E-02	2.7E-02	1.5E-02	1.5E-02	1.6E-02	2.7E-02	2.5E-02	1.8E-02	1.2E-02	2.4E-03	2.6E-02	1.1E-03	1.6E-02	6.2E-04	2.4E-02	4.7E-03	2.1	0.004	**	1.3	0.131		2.0	0.033	*	0.9	0.517		1.5	0.092	
Rhein	2.1E-04	2.4E-04	3.4E-04	5.7E-04	5.7E-04	5.2E-04	2.7E-04	3.1E-04	3.6E-04	3.3E-04	5.7E-04	3.4E-04	2.6E-04	6.9E-05	5.6E-04	3.1E-05	3.1E-04	4.6E-05	4.2E-04	1.4E-04	2.1	0.008	**	1.2	0.364		1.6	0.184		0.7	0.214		1.3	0.321	
5,6,7,8-Tetrahydrobiopterin	1.2E-04	2.4E-05	7.4E-05	7.5E-05	1.3E-04	1.9E-04	1.6E-04	7.1E-05	1.4E-04	1.5E-04	8.3E-05	N.D.	7.2E-05	4.6E-05	1.3E-04	6.0E-05	1.2E-04	4.7E-05	1.2E-04	4.7E-05	1.8	0.245		1.7	0.238		1.6	0.392		0.9	0.768		0.9	0.867	
Fructose 6-phosphate	5.3E-03	6.8E-03	7.6E-03	1.0E-02	1.1E-02	1.1E-02	7.3E-03	8.9E-03	8.7E-03	7.2E-03	6.8E-03	6.4E-03	6.5E-03	1.2E-03	1.1E-02	4.9E-04	8.3E-03	8.6E-04	6.8E-03	4.1E-04	1.6	0.015	*	1.3	0.118		1.0	0.733		0.6	5.4E-04	***	0.8	0.083	
Glucose 6-phosphate	2.3E-02	3.0E-02	3.4E-02	4.3E-02	5.2E-02	4.2E-02	3.0E-02	3.7E-02	3.6E-02	3.4E-02	3.1E-02	2.6E-02	2.9E-02	5.4E-03	4.6E-02	5.2E-03	3.4E-02	3.4E-03	3.0E-02	4.1E-03	1.6	0.017	*	1.2	0.221		1.0	0.752		0.7	0.016	*	0.9	0.245	
Serotonin	2.0E-04	2.4E-04	7.0E-04	8.2E-04	3.5E-04	6.5E-04	2.9E-04	3.5E-04	3.9E-04	3.9E-04	3.7E-04	5.6E-04	3.8E-04	2.8E-04	6.1E-04	2.4E-04	3.4E-04	5.1E-05	4.4E-04	1.0E-04	1.6	0.350		0.9	0.836		1.2	0.751		0.7	0.359		1.3	0.224	
XA0055	2.6E-04	6.4E-04	8.0E-04	7.5E-04	9.3E-04	9.3E-04	N.D.	7.5E-04	8.7E-04	N.D.	4.6E-04	7.7E-04	5.7E-04	2.8E-04	8.7E-04	1.0E-04	8.1E-04	8.9E-05	6.2E-04	2.2E-04	1.5	0.191		1.4	0.266		1.1	0.838		0.7	0.331		0.8	0.417	
Glucose 1-phosphate	2.0E-03	3.3E-03	3.1E-03	3.8E-03	5.1E-03	3.9E-03	2.8E-03	3.8E-03	3.2E-03	3.8E-03	3.6E-03	3.0E-03	2.8E-03	7.3E-04	4.3E-03	7.0E-04	3.3E-03	4.9E-04	3.5E-03	4.3E-04	1.5	0.069		1.2	0.445		1.2	0.270		0.8	0.182		1.1	0.601	
2-Methylserine	4.7E-04	4.3E-04	4.4E-04	6.5E-04	7.9E-04	5.1E-04	7.4E-04	5.1E-04	4.8E-04	9.2E-04	7.3E-04	4.7E-04	4.5E-04	2.1E-05	6.5E-04	1.4E-04	5.8E-04	1.4E-04	7.1E-04	2.2E-04	1.4	0.130		1.3	0.244		1.6	0.183		1.1	0.716		1.2	0.460	
Dihydroxyacetone phosphate	2.6E-03	3.3E-03	5.8E-03	4.8E-03	5.6E-03	6.4E-03	3.6E-03	4.7E-03	5.4E-03	5.5E-03	4.4E-03	5.0E-03	3.9E-03	1.7E-03	5.6E-03	7.8E-04	4.6E-03	8.7E-04	5.0E-03	5.5E-04	1.4	0.215		1.2	0.589		1.3	0.391		0.9	0.318		1.1	0.545	
Uric acid	N.D.	1.5E-04	3.5E-04	4.8E-04	3.9E-04	2.0E-04	1.2E-04	1.9E-04	1.1E-04	2.1E-04	2.6E-04	1.3E-04	2.5E-04	1.5E-04	3.6E-04	1.4E-04	1.4E-04	4.2E-05	2.0E-04	6.5E-05	1.4	0.496		0.6	0.482		0.8	0.718		0.6	0.185		1.4	0.276	
Sedoheptulose 7-phosphate	5.7E-03	7.0E-03	8.0E-03	9.4E-03	1.2E-02	7.9E-03	6.2E-03	8.5E-03	6.0E-03	6.1E-03	7.6E-03	5.3E-03	6.9E-03	1.2E-03	9.6E-03	1.8E-03	6.9E-03	1.4E-03	6.3E-03	1.1E-03	1.4	0.104		1.0	0.984		0.9	0.572		0.7	0.065		0.9	0.620	
Glycerophosphocholine	5.4E-02	4.7E-02	4.3E-02	7.3E-02	5.7E-02	6.8E-02	5.7E-02	4.9E-02	5.5E-02	5.8E-02	6.7E-02	5.2E-02	4.8E-02	5.9E-03	6.6E-02	8.2E-03	5.4E-02	4.3E-03	5.9E-02	7.9E-03	1.4	0.043	*	1.1	0.253		1.2	0.132		0.9	0.358		1.1	0.382	
Ser	2.5E-02	3.3E-02	4.2E-02	4.2E-02	4.9E-02	4.1E-02	3.2E-02	2.7E-02	4.5E-02	4.9E-02	3.3E-02	3.9E-02	3.3E-02	8.4E-03	4.4E-02	4.3E-03	3.5E-02	9.2E-03	4.0E-02	8.0E-03	1.3	0.133		1.0	0.848		1.2	0.341		0.9	0.507		1.2	0.459	
Ribose 5-phosphate	1.7E-03	1.7E-03	2.3E-03	2.3E-03	2.5E-03	2.9E-03	2.5E-03	3.0E-03	4.0E-03	4.1E-03	3.6E-03	4.8E-03	1.9E-03	3.4E-04	2.6E-03	2.9E-04	3.1E-03	7.6E-04	4.2E-03	6.1E-04	1.3	0.069		1.6	0.092		2.2	0.010	**	1.6	0.028	*	1.3	0.140	
Betaine aldehyde_+H₂O	2.1E-03	2.1E-03	2.0E-03	2.6E-03	2.9E-03	2.7E-03	3.0E-03	1.9E-03	3.1E-03	2.9E-03	2.0E-03	2.7E-03	2.1E-03	4.7E-05	2.7E-03	1.6E-04	2.7E-03	6.5E-04	2.5E-03	4.7E-04	1.3	0.013	*	1.3	0.250		1.2	0.255		0.9	0.504		0.9	0.731	
Homocarnosine	N.D.	5.0E-05	N.D.	N.D.	6.3E-05	N.D.	7.0E-05	N.D.	6.0E-05	N.D.	5.1E-05	5.3E-05	5.0E-05	N.A.	6.3E-05	N.A.	6.5E-05	7.0E-06	5.2E-05	1.2E-06	1.3	N.A.		1.3	N.A.		1.1	N.A.		0.8	N.A.		0.8	0.218	
S-Methylglutathione	2.1E-03	2.2E-03	2.3E-03	2.7E-03	3.0E-03	2.7E-03	2.9E-03	2.7E-03	2.7E-03	3.1E-03	2.7E-03	2.9E-03	2.2E-03	1.3E-04	2.8E-03	1.7E-04	2.8E-03	1.0E-04	2.9E-03	1.7E-04	1.3	0.011	*	1.2	0.005	**	1.3	0.005	**	1.0	0.465		1.1	0.240	
Glucosamine	7.7E-04	1.2E-03	1.1E-03	1.2E-03	1.5E-03	1.3E-03	8.2E-04	8.1E-04	8.4E-04	1.0E-03	1.0E-03	2.0E-03	1.0E-03	2.5E-04	1.3E-03	1.5E-04	8.2E-04	1.5E-05	1.4E-03	6.0E-04	1.3	0.187		0.8	0.256		1.3	0.481		1.0	0.945		1.6	0.264	
Acetyl CoA_divalent	9.0E-04	1.7E-03	1.3E-03	1.2E-03	1.8E-03	1.8E-03	1.2E-03	1.1E-03	1.5E-03	9.9E-04	9.6E-04	2.1E-03	1.3E-03	3.8E-04	1.6E-03	3.5E-04	1.3E-03	2.4E-04	1.4E-03	6.5E-04	1.3	0.312		1.0	0.957		1.0	0.911		0.8	0.542		1.1	0.878	
His	7.9E-02	8.7E-02	9.5E-02	1.1E-01	1.2E-01	9.6E-02	1.0E-01	9.0E-02	1.0E-01	9.4E-02	8.0E-02	9.0E-02	8.7E-02	8.0E-03	1.1E-01	1.4E-02	1.0E-01	8.0E-03	8.8E-02	7.0E-03	1.3	0.086		1.1	0.137		1.0	0.929		0.8	0.092		0.9	0.134	
Cytosine	4.1E-05	4.2E-05	3.7E-05	6.4E-05	5.1E-05	3.6E-05	6.6E-05	3.3E-05	3.6E-05	6.3E-05	5.3E-05	6.4E-05	4.0E-05	2.3E-06	5.0E-05	1.4E-05	4.5E-05	1.8E-05	6.0E-05	6.3E-06	1.3	0.343		1.1	0.684		1.5	0.022	*	1.2	0.379		1.3	0.299	
cis-4-Hydroxyproline	2.2E-04	1.7E-04	1.8E-04	2.3E-04	2.1E-04	2.8E-04	3.4E-04	3.2E-04	4.6E-04	3.9E-04	3.1E-04	4.1E-04	1.9E-04	2.3E-05	2.4E-04	3.7E-05	3.7E-04	7.5E-05	3.7E-04	5.3E-05	1.3	0.146		1.9	0.043	*	2.0	0.015	*	1.6	0.027	*	1.0	0.984	
IMP	6.2E-03	7.7E-03	7.4E-03	9.5E-03	1.0E-02	7.0E-03	5.6E-03	5.6E-03	5.2E-03	6.4E-03	7.0E-03	5.3E-03	7.1E-03	7.7E-04	8.8E-03	1.6E-03	5.5E-03	2.0E-04	6.3E-03	8.7E-04	1.2	0.193		0.8	0.058		0.9	0.281		0.7	0.089		1.1	0.243	
Asp	4.1E-02	4.9E-02	3.7E-02	4.7E-02	6.2E-02	4.7E-02	6.2E-02	5.4E-02	7.0E-02	7.1E-02	3.8E-02	4.7E-02	4.2E-02	5.8E-03	5.2E-02	8.9E-03	6.2E-02	8.2E-03	5.2E-02	1.7E-02	1.2	0.198		1.5	0.031	*	1.2	0.424		1.0	0.986		0.8	0.428	
Pyruvic acid	N.D.	4.3E-03	4.1E-03	N.D.	5.1E-03	5.3E-03	4.2E-03	5.0E-03	N.D.	N.D.	5.8E-03	5.3E-03	4.2E-03	1.4E-04	5.2E-03	1.5E-04	4.6E-03	5.9E-04	5.6E-03	3.6E-04	1.2	0.021	*	1.1	0.547		1.3	0.089		1.1	0.372		1.2	0.211	
3-Phosphoglyceric acid	8.8E-04	8.2E-04	1.3E-03	1.3E-03	1.1E-03	1.3E-03	8.9E-04	1.2E-03	1.9E-03	1.5E-03	1.2E-03	1.9E-03	1.0E-03	2.8E-04	1.2E-03	9.8E-05	1.3E-03	5.5E-04	1.5E-03	3.6E-04	1.2	0.294		1.3	0.431		1.5	0.123		1.2	0.290		1.1	0.641	
Cys	4.6E-04	3.8E-04	5.1E-04	4.7E-04	6.9E-04	4.8E-04	6.1E-04	4.5E-04	5.3E-04	6.1E-04	6.9E-04	2.7E-04	4.5E-04	6.5E-05	5.5E-04	1.2E-04	5.3E-04	8.2E-05	5.2E-04	2.2E-04	1.2	0.301		1.2	0.244		1.2	0.629		1.0	0.876		1.0	0.952	
threo-β-Methylaspartic acid	4.1E-04	4.9E-04	4.8E-04	5.8E-04	5.4E-04	5.4E-04	4.2E-04	3.5E-04	4.1E-04	4.3E-04	4.3E-04	4.1E-04	4.6E-04	4.3E-05	5.5E-04	2.6E-05	3.9E-04	3.9E-05	4.2E-04	1.2E-05	1.2	0.036	*	0.9	0.127		0.9	0.291		0.8	0.006	**	1.1	0.321	
2,4-Diaminobutyric acid	1.4E-04	N.D.	1.4E-04	1.5E-04	1.8E-04	1.8E-04	2.0E-04	1.3E-04	1.4E-04	2.1E-04	1.8E-04	N.D.	1.4E-04	2.0E-06	1.7E-04	1.7E-05	1.6E-04	3.6E-05	2.0E-04	2.0E-05	1.2	0.091		1.1	0.459		1.4	0.149		1.2	0.245		1.2	0.215	
Triethanolamine	1.8E-04	2.0E-04	2.2E-04	2.2E-04	3.1E-04	1.9E-04	4.0E-04	2.5E-04	1.3E-04	2.9E-04	1.8E-04	1.9E-04	2.0E-04	2.3E-05	2.4E-04	6.5E-05	2.6E-04	1.4E-04	2.2E-04	5.9E-05	1.2	0.380		1.3	0.512		1.1	0.572		0.9	0.731		0.8	0.682	
1-Methylhistamine	7.6E-05	9.8E-05	1.2E-04	8.0E-05	1.4E-04	1.3E-04	1.2E-04	8.9E-05	1.0E-04	1.3E-04	1.0E-04	1.4E-04	9.7E-05	2.1E-05	1.2E-04	3.3E-05	1.1E-04	1.8E-05	1.2E-04	2.2E-05	1.2	0.432		1.1	0.657		1.3	0.193		1.1	0.774		1.2	0.301	
Diethanolamine	2.5E-04	2.5E-04	3.6E-04	3.4E-04	3.6E-04	3.4E-04	4.1E-04	2.1E-04	3.2E-04	3.7E-04	3.5E-04	2.4E-04	2.9E-04	6.5E-05	3.5E-04	9.9E-06	3.1E-04	1.0E-04	3.2E-04	7.1E-05	1.2	0.255		1.1	0.724		1.1	0.636		0.9	0.529		1.0	0.979	
Glutathione (GSH)	4.4E-01	3.9E-01	4.3E-01	4.4E-01	5.4E-01	5.4E-01	5.6E-01	4.6E-01	4.9E-01	5.1E-01	4.6E-01	3.6E-01	4.2E-01	2.9E-02	5.0E-01	5.7E-02	5.0E-01	5.0E-02	4.4E-01	7.6E-02	1.2	0.110		1.2	0.082		1.1	0.659		0.9	0.337		0.9	0.330	
$NADP^+$	5.7E-03	4.2E-03	3.8E-03	5.1E-03	5.3E-03	6.0E-03	4.8E-03	4.3E-03	3.6E-03	3.9E-03	3.7E-03	2.8E-03	4.5E-03	1.0E-03	5.5E-03	4.6E-04	4.2E-03	5.7E-04	3.4E-03	5.9E-04	1.2	0.257		0.9	0.669		0.8	0.192		0.6	0.011	*	0.8	0.168	
Gln	2.6E-01	2.9E-01	2.7E-01	3.0E-01	3.7E-01	3.1E-01	2.9E-01	2.7E-01	2.6E-01	2.7E-01	2.3E-01	2.6E-01	2.7E-01	1.7E-02	3.3E-01	4.0E-02	2.7E-01	1.6E-02	2.5E-01	1.8E-02	1.2	0.130		1.0	0.913		0.9	0.209		0.8	0.065		0.9	0.175	
XC0089	1.1E-04	1.3E-04	1.8E-04	1.7E-04	1.4E-04	2.0E-04	1.1E-04	1.1E-04	8.5E-05	7.5E-05	6.2E-05	7.5E-05	1.4E-04	3.7E-05	1.7E-04	3.1E-05	1.0E-04	1.7E-05	7.1E-05	7.6E-06	1.2	0.379		0.7	0.208		0.5	0.073		0.4	0.025	*	0.7	0.058	
GDP-glucose GDP-mannose GDP-galactose	8.1E-04	9.7E-04	1.1E-03	7.2E-04	1.4E-03	1.3E-03	8.5E-04	8.9E-04	1.0E-03	1.0E-03	1.1E-03	7.7E-04	9.6E-04	1.4E-04	1.1E-03	3.7E-04	9.2E-04	8.9E-05	9.6E-04	1.7E-04	1.2	0.491		1.0	0.696		1.0	0.998		0.8	0.498		1.0	0.734	
6-Phosphogluconic acid	2.0E-03	2.6E-03	2.4E-03	2.4E-03	2.9E-03	3.0E-03	2.6E-03	2.6E-03	2.3E-03	3.1E-03	2.9E-03	2.6E-03	2.3E-03	3.1E-04	2.7E-03	3.1E-04	2.5E-03	1.8E-04	2.9E-03	2.6E-04	1.2	0.163		1.1	0.448		1.2	0.084		1.0	0.662		1.1	0.128	
Spermine	6.1E-04	5.5E-04	7.9E-04	6.5E-04	7.7E-04	8.7E-04	9.0E-04	8.1E-04	9.0E-04	7.6E-04	8.7E-04	8.8E-04	6.5E-04	1.3E-04	7.6E-04	1.1E-04	8.7E-04	5.4E-05	8.4E-04	6.4E-05	1.2	0.298		1.3	0.074		1.3	0.103		1.1	0.384		1.0	0.531	
S-Lactoylglutathione	3.0E-04	3.5E-04	3.2E-04	3.7E-04	3.5E-04	4.0E-04	3.6E-04	3.1E-04	3.3E-04	3.6E-04	2.4E-04	2.8E-04	3.2E-04	2.5E-05	3.7E-04	2.3E-05	3.3E-04	2.8E-05	2.9E-04	6.1E-05	1.2	0.066		1.0	0.626		0.9	0.473		0.8	0.132		0.9	0.350	
Ribulose 5-phosphate	8.3E-03	9.7E-03	1.2E-02	1.0E-02	1.3E-02	1.2E-02	8.5E-03	1.1E-02	9.7E-03	1.0E-02	1.2E-02	1.1E-02	1.0E-02	2.0E-03	1.2E-02	1.2E-03	9.7E-03	1.2E-03	1.1E-02	6.2E-04	1.2	0.333		1.0	0.814		1.1	0.512		0.9	0.498		1.1	0.206	
XC0132	6.0E-04	7.0E-04	8.2E-04	7.6E-04	8.2E-04	8.5E-04	8.5E-04	6.5E-04	6.0E-04	6.2E-04	6.2E-04	8.3E-04	7.1E-04	1.1E-04	8.1E-04	4.8E-05	7.0E-04	1.3E-04	6.9E-04	1.2E-04	1.1	0.240		1.0	0.949		1.0	0.875		0.9	0.230		1.0	0.935	
ATP	4.9E-02	4.2E-02	3.3E-02	4.5E-02	4.3E-02	5.2E-02	4.7E-02	3.8E-02	4.8E-02	3.9E-02	3.1E-02	2.3E-02	4.1E-02	8.1E-03	4.7E-02	4.7E-03	4.4E-02	5.6E-03	3.1E-02	8.2E-03	1.1	0.368		1.1	0.644		0.8	0.203		0.7	0.059		0.7	0.096	
N-Glycolylneuraminic acid	1.7E-03	1.5E-03	1.5E-03	1.6E-03	1.8E-03	1.8E-03	1.4E-03	1.9E-03	1.5E-03	1.6E-03	1.5E-03	1.4E-03	1.5E-03	1.1E-04	1.7E-03	1.1E-04	1.6E-03	2.6E-04	1.5E-03	8.2E-05	1.1	0.073		1.1	0.655		1.0	0.769		0.9	0.044	*	0.9	0.560	
N-Acetylglutamine	7.4E-04	1.0E-03	7.4E-04	7.6E-04	1.1E-03	1.0E-03	9.3E-04	8.6E-04	1.1E-03	9.2E-04	7.5E-04	8.7E-04	8.3E-04	1.6E-04	9.4E-04	1.6E-04	9.6E-04	1.2E-04	8.5E-04	8.5E-05	1.1	0.436		1.2	0.331		1.0	0.895		0.9	0.416		0.9	0.260	
Taurocholic acid	4.0E-02	5.2E-02	7.2E-02	7.7E-02	4.3E-02	6.5E-02	3.9E-02	3.4E-02	4.0E-02	5.8E-02	4.6E-02	4.8E-02	5.5E-02	1.6E-02	6.2E-02	1.8E-02	3.8E-02	3.4E-03	5.1E-02	6.7E-03	1.1	0.626		0.7	0.205		0.9	0.728		0.8	0.390		1.4	0.057	
Metronidazole	2.7E-04	3.3E-04	3.2E-04	4.3E-04	3.1E-04	3.1E-04	2.6E-04	3.7E-04	2.7E-04	2.9E-04	3.8E-04	3.4E-04	3.1E-04	3.3E-05	3.5E-04	6.9E-05	3.0E-04	5.8E-05	3.4E-04	4.3E-05	1.1	0.433		1.0	0.790		1.1	0.473		1.0	0.765		1.1	0.442	
Thiamine	4.1E-04	5.0E-04	6.0E-04	4.9E-04	5.6E-04	6.6E-04	4.9E-04	3.2E-04	4.8E-04	5.5E-04	5.5E-04	5.7E-04	5.1E-04	9.4E-05	5.7E-04	9.0E-05	4.3E-04	9.6E-05	5.6E-04	8.1E-06	1.1	0.440		0.8	0.380		1.1	0.443		1.0	0.829		1.3	0.147	
UDP-*N*-acetylglucosamine	1.3E-02	1.2E-02	1.2E-02	1.4E-02	1.5E-02	1.3E-02	1.2E-02	1.2E-02	1.2E-02	1.3E-02	1.3E-02	1.2E-02	1.3E-02	7.5E-04	1.4E-02	1.1E-03	1.2E-02	1.9E-04	1.2E-02	5.4E-04	1.1	0.111		1.0	0.566		1.0	0.846		0.9	0.092		1.0	0.620	
Xanthosine	9.6E-04	1.5E-03	1.9E-03	1.5E-03	1.9E-03	1.5E-03	7.1E-04	4.7E-04	6.2E-04	7.3E-04	1.2E-03	1.1E-03	1.5E-03	4.8E-04	1.6E-03	2.5E-04	6.0E-04	1.2E-04	1.0E-03	2.4E-04	1.1	0.621		0.4	0.084		0.7	0.245		0.6	0.037	*	1.7	0.082	
N^8-Acetylspermidine	2.8E-05	4.6E-05	1.0E-04	4.1E-05	7.7E-05	7.5E-05	7.1E-05	8.2E-05	7.1E-05	7.9E-05	4.8E-05	7.6E-05	5.8E-05	3.7E-05	6.5E-05	2.0E-05	7.5E-05	6.5E-06	6.7E-05	1.7E-05	1.1	0.799		1.3	0.512		1.2	0.712		1.0	0.858		0.9	0.543	
Adenosine	2.1E-02	1.5E-02	1.5E-02	1.8E-02	1.7E-02	2.1E-02	1.7E-02	2.4E-02	1.6E-02	1.5E-02	1.7E-02	1.7E-02	1.7E-02	3.4E-03	1.9E-02	2.0E-03	1.9E-02	4.5E-03	1.6E-02	1.2E-03	1.1	0.454		1.2	0.476		1.0	0.890		0.9	0.182		0.8	0.384	
Malic acid	4.4E-02	5.6E-02	4.6E-02	4.6E-02	6.3E-02	5.2E-02	7.5E-02	6.5E-02	7.1E-02	9.6E-02	4.9E-02	6.0E-02	4.9E-02	6.2E-03	5.4E-02	8.2E-03	7.0E-02	4.9E-03	6.9E-02	2.5E-02	1.1	0.440		1.4	0.010	**	1.4	0.292		1.3	0.408		1.0	0.909	
Arg	5.5E-04	7.1E-04	9.2E-04	7.9E-04	8.4E-04	7.5E-04	1.4E-03	7.0E-04	8.4E-04	9.5E-04	8.4E-04	6.9E-04	7.3E-04	1.9E-04	8.0E-04	4.4E-05	9.9E-04	3.8E-04	8.2E-04	1.3E-04	1.1	0.585		1.4	0.364		1.1	0.503		1.0	0.756		0.8	0.542	
S-Adenosylmethionine	4.0E-03	4.3E-03	4.2E-03	4.3E-03	5.1E-03	4.4E-03	6.2E-03	5.2E-03	5.4E-03	6.0E-03	5.5E-03	6.1E-03	4.2E-03	1.4E-04	4.6E-03	4.3E-04	5.6E-03	5.5E-04	5.9E-03	3.1E-04	1.1	0.244		1.3	0.039	*	1.4	0.004	**	1.3	0.015	*	1.1	0.468	
N-Acetylglucosamine	4.5E-04	3.8E-04	4.5E-04	4.2E-04	4.3E-04	5.5E-04	4.9E-04	5.4E-04	4.4E-04	3.9E-04	3.9E-04	4.6E-04	4.3E-04	4.3E-05	4.7E-04	7.4E-05	4.9E-04	4.8E-05	4.2E-04	4.1E-05	1.1	0.470		1.1	0.166		1.0	0.775		0.9	0.373		0.9	0.116	
γ-Glu-2-aminobutyric acid	3.0E-04	3.3E-04	2.5E-04	3.1E-04	3.0E-04	3.6E-04	4.1E-04	3.2E-04	3.2E-04	3.1E-04	2.8E-04	3.4E-04	3.0E-04	3.9E-05	3.2E-04	2.9E-05	3.5E-04	5.4E-05	3.1E-04	3.4E-05	1.1	0.385		1.2	0.230		1.0	0.671		1.0	0.618		0.9	0.333	
N-Acetylhistidine	9.0E-04	1.2E-03	9.3E-04	1.0E-03	1.3E-03	1.0E-03	1.2E-03	1.1E-03	1.3E-03	1.2E-03	8.6E-04	1.2E-03	1.0E-03	1.9E-04	1.1E-03	1.4E-04	1.2E-03	8.0E-05	1.1E-03	1.9E-04	1.1	0.543		1.2	0.260		1.1	0.743		1.0	0.803		0.9	0.415	
XA0019	3.6E-03	6.2E-04	3.3E-03	4.0E-03	1.8E-03	2.4E-03	2.6E-03	4.6E-03	2.7E-03	3.0E-03	N.D.	7.7E-03	2.5E-03	1.6E-03	2.7E-03	1.1E-03	3.3E-03	1.2E-03	5.4E-03	3.4E-03	1.1	0.862		1.3	0.540		2.1	0.430		2.0	0.462		1.6	0.543	
Asn	7.2E-03	7.6E-03	1.1E-02	8.8E-03	9.9E-03	8.7E-03	8.7E-03	7.6E-03	8.5E-03	8.9E-03	9.3E-03	7.9E-03	8.5E-03	1.8E-03	9.1E-03	6.6E-04	8.3E-03	5.8E-04	8.7E-03	7.3E-04	1.1	0.595		1.0	0.873		1.0	0.839		1.0	0.499		1.1	0.456	
Homoserinelactone	5.2E-04	6.0E-04	4.8E-04	6.0E-04	5.8E-04	5.5E-04	9.1E-04	7.6E-04	7.6E-04	8.9E-04	7.0E-04	8.7E-04	5.3E-04	6.3E-05	5.8E-04	2.5E-05	8.1E-04	8.4E-05	8.2E-04	1.1E-04	1.1	0.372		1.5	0.012	*	1.5	0.025	*	1.4	0.054		1.0	0.905	
1-Methylnicotinamide	4.7E-04	6.5E-04	1.0E-03	7.6E-04	8.4E-04	6.8E-04	8.5E-04	8.9E-04	9.3E-04	8.0E-04	6.5E-04	8.5E-04	7.0E-04	2.7E-04	7.6E-04	7.9E-05	8.9E-04	3.6E-05	7.7E-04	1.0E-04	1.1	0.762		1.3	0.349		1.1	0.724		1.0	0.896		0.9	0.165	
Cysteinesulfinic acid	1.1E-03	1.1E-03	6.5E-04	1.1E-03	9.6E-04	1.1E-03	1.2E-03	1.3E-03	1.2E-03	8.5E-04	1.0E-03	1.1E-03	9.5E-04	2.6E-04	1.0E-03	5.3E-05	1.2E-03	9.0E-05	1.0E-03	1.5E-04	1.1	0.679		1.3	0.203		1.1	0.750		1.0	0.899		0.8	0.114	
Choline	4.6E-02	5.2E-02	6.7E-02	6.2E-02	6.3E-02	5.2E-02	7.0E-02	5.1E-02	6.4E-02	6.3E-02	5.9E-02	6.2E-02	5.5E-02	1.1E-02	5.9E-02	5.7E-03	6.2E-02	9.7E-03	6.1E-02	2.2E-03	1.1	0.617		1.1	0.447		1.1	0.418		1.0	0.545		1.0	0.912	
CoA_divalent	1.2E-02	9.4E-03	1.1E-02	1.1E-02	1.1E-02	1.3E-02	1.0E-02	9.8E-03	1.2E-02	9.9E-03	9.2E-03	6.7E-03	1.1E-02	1.2E-03	1.1E-02	1.1E-03	1.1E-02	1.0E-03	8.6E-03	1.7E-03	1.1	0.497		1.0	0.808		0.8	0.151		0.7	0.081		0.8	0.180	
Thiamine diphosphate	7.3E-04	6.8E-04	6.7E-04	7.3E-04	7.2E-04	7.5E-04	6.7E-04	5.6E-04	5.3E-04	6.0E-04	6.2E-04	5.5E-04	6.9E-04	3.0E-05	7.4E-04	1.6E-05	5.8E-04	7.6E-05	5.9E-04	3.8E-05	1.1	0.108		0.8	0.120		0.9	0.026	*	0.8	0.012	*	1.0	0.869	
Threonic acid	1.1E-03	7.1E-04	9.6E-04	1.1E-03	8.0E-04	9.9E-04	1.1E-03	1.3E-03	1.0E-03	1.0E-03	6.9E-04	1.4E-03	9.1E-04	1.8E-04	9.6E-04	1.5E-04	1.1E-03	1.3E-04	1.1E-03	3.7E-04	1.1	0.702		1.2	0.180		1.2	0.590		1.1	0.731		0.9	0.789	
Inosine	2.2E-02	3.4E-02	6.0E-02	4.4E-02	4.1E-02	3.8E-02	1.8E-02	2.8E-02	2.2E-02	2.7E-02	3.1E-02	3.4E-02	3.9E-02	2.0E-02	4.1E-02	2.9E-03	2.3E-02	5.1E-03	3.1E-02	3.4E-03	1.1	0.857		0.6	0.295		0.8	0.545		0.7	0.016	*	1.3	0.106	
ADP	4.4E-02	4.0E-02	3.6E-02	4.1E-02	3.9E-02	4.6E-02	4.4E-02	3.9E-02	4.3E-02	4.2E-02	3.8E-02	3.5E-02	4.0E-02	4.2E-03	4.2E-02	3.7E-03	4.2E-02	2.6E-03	3.9E-02	3.5E-03	1.1	0.557		1.1	0.454		1.0	0.738		0.9	0.335		0.9	0.237	
Thiamine phosphate	9.5E-04	8.5E-04	9.6E-04	8.7E-04	9.2E-04	1.1E-03	7.3E-04	7.4E-04	7.4E-04	7.3E-04	7.9E-04	8.7E-04	9.2E-04	6.0E-05	9.6E-04	1.2E-04	7.4E-04	5.5E-06	8.0E-04	6.8E-05	1.1	0.592		0.8	0.034	*	0.9	0.083		0.8	0.127		1.1	0.275	
Fumaric acid	9.4E-03	1.1E-02	8.5E-03	8.6E-03	1.1E-02	1.1E-02	1.3E-02	1.2E-02	1.4E-02	1.6E-02	1.0E-02	1.1E-02	9.7E-03	1.3E-03	1.0E-02	1.3E-03	1.3E-02	1.0E-03	1.2E-02	3.0E-03	1.0	0.682		1.3	0.033	*	1.3	0.280		1.2	0.365		0.9	0.739	

Thr	4.1E-02	4.3E-02	5.1E-02	4.7E-02	4.9E-02	4.6E-02	4.8E-02	4.1E-02	5.2E-02	5.2E-02	5.1E-02	4.6E-02	4.5E-02	5.4E-03	4.7E-02	1.7E-03	4.7E-02	5.6E-03	5.0E-02	2.9E-03	1.0	0.556		1.0	0.692		1.1	0.272		1.1	0.282		1.1	0.497	
Spermidine	3.3E-03	4.3E-03	5.8E-03	4.7E-03	5.0E-03	4.3E-03	5.8E-03	5.3E-03	5.7E-03	6.0E-03	5.7E-03	6.6E-03	4.5E-03	1.2E-03	4.7E-03	3.4E-04	5.6E-03	2.8E-04	6.1E-03	4.5E-04	1.0	0.813		1.3	0.250		1.4	0.142		1.3	0.014	*	1.1	0.211	
XC0126	3.6E-04	3.6E-04	2.5E-04	3.3E-04	3.7E-04	3.1E-04	2.7E-04	2.5E-04	2.6E-04	2.8E-04	2.5E-04	2.9E-04	3.2E-04	6.3E-05	3.4E-04	2.8E-05	2.6E-04	7.2E-06	2.7E-04	2.3E-05	1.0	0.785		0.8	0.226		0.8	0.294		0.8	0.040	*	1.0	0.482	
GTP	4.0E-03	3.4E-03	2.2E-03	3.3E-03	3.5E-03	3.2E-03	2.9E-03	2.5E-03	3.3E-03	1.6E-03	1.1E-03	8.8E-04	3.2E-03	8.9E-04	3.3E-03	1.6E-04	2.9E-03	3.8E-04	1.2E-03	3.6E-04	1.0	0.848		0.9	0.632		0.4	0.043	*	0.4	0.004	**	0.4	0.005	**
XA0065	2.9E-03	2.5E-03	2.6E-03	2.9E-03	3.0E-03	2.2E-03	3.1E-03	2.6E-03	3.5E-03	2.8E-03	2.2E-03	2.5E-03	2.6E-03	1.9E-04	2.7E-03	4.4E-04	3.1E-03	4.3E-04	2.5E-03	3.2E-04	1.0	0.774		1.2	0.213		1.0	0.595		0.9	0.537		0.8	0.148	
2,3-Diphosphoglyceric	5.8E-03	5.4E-03	8.0E-03	5.9E-03	6.4E-03	7.4E-03	7.0E-03	8.8E-03	8.5E-03	7.9E-03	7.5E-03	7.8E-03	6.4E-03	1.4E-03	6.6E-03	7.7E-04	8.1E-03	9.5E-04	7.7E-03	1.6E-04	1.0	0.857		1.3	0.168		1.2	0.245		1.2	0.120		1.0	0.563	
XA0012	1.2E-03	1.4E-03	1.1E-03	1.0E-03	1.6E-03	1.3E-03	1.5E-03	1.2E-03	1.1E-03	9.1E-04	1.0E-03	1.5E-03	1.3E-03	1.6E-04	1.3E-03	3.0E-04	1.3E-03	1.9E-04	1.2E-03	3.2E-04	1.0	0.887		1.0	0.884		0.9	0.658		0.9	0.629		0.9	0.601	
Ascorbic acid	3.2E-02	3.8E-02	3.1E-02	2.8E-02	3.8E-02	3.7E-02	3.4E-02	3.2E-02	3.4E-02	4.0E-02	5.3E-02	8.5E-03	3.4E-02	3.7E-03	3.4E-02	5.3E-03	3.3E-02	1.1E-03	3.4E-02	2.3E-02	1.0	0.848		1.0	0.933		1.0	0.985		1.0	0.974		1.0	0.974	
NMN	4.9E-04	3.3E-04	3.0E-04	3.8E-04	3.4E-04	4.2E-04	2.8E-04	3.2E-04	2.9E-04	2.6E-04	3.0E-04	3.7E-04	3.7E-04	1.0E-04	3.8E-04	4.0E-05	3.0E-04	1.7E-05	3.1E-04	5.4E-05	1.0	0.912		0.8	0.328		0.8	0.419		0.8	0.150		1.0	0.713	
Guanosine	2.1E-03	1.9E-03	2.2E-03	2.1E-03	2.1E-03	2.1E-03	2.2E-03	2.6E-03	1.9E-03	2.3E-03	2.7E-03	2.4E-03	2.1E-03	1.2E-04	2.1E-03	2.8E-06	2.3E-03	3.5E-04	2.4E-03	2.1E-04	1.0	0.609		1.1	0.389		1.2	0.062		1.2	0.099		1.1	0.503	
UTP	4.3E-03	3.8E-03	2.4E-03	3.6E-03	3.3E-03	3.7E-03	4.3E-03	2.7E-03	4.6E-03	1.8E-03	1.2E-03	9.5E-04	3.5E-03	9.8E-04	3.6E-03	2.5E-04	3.8E-03	9.9E-04	1.3E-03	4.4E-04	1.0	0.928		1.1	0.685		0.4	0.045	*	0.4	0.004	**	0.3	0.032	*
Glycerol 3-phosphate	8.8E-02	9.0E-02	8.6E-02	8.7E-02	9.1E-02	9.1E-02	8.4E-02	7.1E-02	8.2E-02	9.1E-02	8.7E-02	9.8E-02	8.8E-02	2.0E-03	9.0E-02	2.6E-03	7.9E-02	6.8E-03	9.2E-02	5.6E-03	1.0	0.547		0.9	0.134		1.0	0.347		1.0	0.508		1.2	0.063	
Pelargonic acid	6.2E-04	5.3E-04	4.7E-04	5.6E-04	5.3E-04	N.D.	6.8E-04	4.2E-04	4.7E-04	5.9E-04	5.7E-04	4.6E-04	5.4E-04	7.6E-05	5.4E-04	2.0E-05	5.2E-04	1.4E-04	5.4E-04	6.7E-05	1.0	0.891		1.0	0.895		1.0	0.961		1.0	0.931		1.0	0.868	
Glu	1.3E-01	1.8E-01	1.0E-01	1.2E-01	1.7E-01	1.3E-01	1.8E-01	1.4E-01	1.5E-01	1.6E-01	1.0E-01	1.6E-01	1.4E-01	3.8E-02	1.4E-01	2.8E-02	1.6E-01	2.0E-02	1.4E-01	3.2E-02	1.0	0.956		1.2	0.426		1.0	0.969		1.0	0.987		0.9	0.388	
Ala	1.2E-02	1.4E-02	1.2E-02	1.2E-02	1.4E-02	1.2E-02	1.8E-02	1.3E-02	1.7E-02	1.6E-02	1.3E-02	1.4E-02	1.3E-02	1.2E-03	1.3E-02	7.1E-04	1.6E-02	2.3E-03	1.4E-02	1.5E-03	1.0	0.879		1.3	0.108		1.1	0.243		1.1	0.246		0.9	0.307	
Hypoxanthine	6.3E-03	1.0E-02	1.6E-02	1.1E-02	1.1E-02	1.1E-02	5.7E-03	8.2E-03	6.1E-03	8.3E-03	9.9E-03	1.1E-02	1.1E-02	4.9E-03	1.1E-02	2.9E-04	6.7E-03	1.4E-03	9.7E-03	1.4E-03	1.0	0.976		0.6	0.280		0.9	0.744		0.9	0.277		1.5	0.053	
Xanthine	3.6E-03	6.1E-03	8.5E-03	5.8E-03	6.8E-03	5.6E-03	3.2E-03	4.2E-03	3.4E-03	4.0E-03	4.8E-03	4.3E-03	6.0E-03	2.4E-03	6.1E-03	6.5E-04	3.6E-03	5.3E-04	4.4E-03	4.3E-04	1.0	0.977		0.6	0.220		0.7	0.352		0.7	0.024	*	1.2	0.127	
GDP	4.6E-03	4.2E-03	3.1E-03	3.9E-03	3.9E-03	4.1E-03	4.2E-03	3.6E-03	3.9E-03	2.2E-03	3.7E-04	1.6E-03	4.0E-03	7.8E-04	4.0E-03	1.4E-04	3.9E-03	2.9E-04	1.4E-03	9.4E-04	1.0	0.954		1.0	0.918		0.4	0.024	*	0.3	0.039	*	0.4	0.035	*
Isethionic acid	3.0E-03	3.6E-03	3.1E-03	3.0E-03	3.5E-03	3.3E-03	2.3E-03	2.8E-03	2.1E-03	2.3E-03	3.2E-03	2.6E-03	3.2E-03	3.2E-04	3.3E-03	2.6E-04	2.4E-03	3.7E-04	2.7E-03	4.6E-04	1.0	0.937		0.7	0.044	*	0.8	0.160		0.8	0.143		1.1	0.491	
Histamine	1.5E-04	1.9E-04	1.8E-04	1.8E-04	1.6E-04	1.8E-04	1.4E-04	1.2E-04	1.5E-04	5.7E-05	1.5E-04	1.9E-04	1.7E-04	2.5E-05	1.8E-04	1.1E-05	1.4E-04	1.5E-05	1.3E-04	6.8E-05	1.0	0.959		0.8	0.100		0.8	0.394		0.8	0.382		1.0	0.933	
Pantothenic acid	4.2E-04	3.6E-04	3.6E-04	3.2E-04	4.0E-04	4.1E-04	4.8E-04	3.6E-04	4.9E-04	3.4E-04	4.2E-04	3.5E-04	3.8E-04	3.6E-05	3.8E-04	5.0E-05	4.5E-04	7.2E-05	3.7E-04	4.1E-05	1.0	0.989		1.2	0.246		1.0	0.800		1.0	0.820		0.8	0.206	
N-Acetylneuraminic acid	3.0E-04	4.6E-04	3.1E-04	3.3E-04	4.0E-04	3.4E-04	3.4E-04	3.5E-04	3.3E-04	3.1E-04	4.1E-04	3.4E-04	3.6E-04	8.9E-05	3.6E-04	4.0E-05	3.4E-04	1.0E-05	3.5E-04	5.3E-05	1.0	0.998		1.0	0.777		1.0	0.952		1.0	0.920		1.0	0.717	
Betaine	2.1E-02	2.0E-02	2.1E-02	2.2E-02	2.0E-02	2.0E-02	3.1E-02	2.4E-02	2.8E-02	2.9E-02	2.5E-02	2.8E-02	2.1E-02	4.5E-04	2.1E-02	1.4E-03	2.8E-02	3.4E-03	2.7E-02	1.9E-03	1.0	0.998		1.3	0.067		1.3	0.025	*	1.3	0.013	*	1.0	0.710	
Imidazolelactic acid	3.2E-04	2.8E-04	3.5E-04	2.9E-04	3.2E-04	3.3E-04	3.6E-04	4.3E-04	4.2E-04	4.6E-04	2.7E-04	3.7E-04	3.2E-04	3.5E-05	3.2E-04	2.3E-05	4.1E-04	3.7E-05	3.7E-04	9.8E-05	1.0	0.958		1.3	0.040	*	1.2	0.488		1.2	0.473		0.9	0.568	
XC0137	5.6E-03	4.7E-03	4.6E-03	5.4E-03	4.6E-03	4.8E-03	5.5E-03	5.2E-03	5.5E-03	4.0E-03	3.0E-03	2.0E-03	5.0E-03	5.4E-04	5.0E-03	4.0E-04	5.4E-03	1.7E-04	3.0E-03	1.0E-03	1.0	0.953		1.1	0.329		0.6	0.059		0.6	0.066		0.6	0.053	
Tyr	1.2E-02	1.4E-02	1.5E-02	1.3E-02	1.4E-02	1.3E-02	1.4E-02	1.2E-02	1.7E-02	1.7E-02	1.4E-02	1.9E-02	1.4E-02	1.5E-03	1.4E-02	5.6E-04	1.4E-02	2.3E-03	1.7E-02	2.2E-03	1.0	0.923		1.0	0.766		1.2	0.160		1.2	0.150		1.2	0.287	
Dyphylline	5.8E-03	6.6E-03	8.6E-03	6.6E-03	6.4E-03	7.7E-03	8.0E-03	5.6E-03	6.6E-03	6.9E-03	5.4E-03	6.1E-03	7.0E-03	1.4E-03	6.9E-03	7.0E-04	6.7E-03	1.2E-03	6.1E-03	7.3E-04	1.0	0.949		1.0	0.799		0.9	0.408		0.9	0.234		0.9	0.511	
NAD^+	2.1E-02	1.8E-02	1.6E-02	1.8E-02	1.7E-02	1.9E-02	1.8E-02	1.6E-02	1.9E-02	1.7E-02	1.6E-02	1.4E-02	1.8E-02	2.9E-03	1.8E-02	1.3E-03	1.8E-02	1.7E-03	1.5E-02	1.6E-03	1.0	0.933		1.0	0.772		0.8	0.216		0.8	0.080		0.9	0.150	
Gly	1.5E-01	1.5E-01	1.7E-01	1.5E-01	1.5E-01	1.7E-01	1.7E-01	1.3E-01	1.7E-01	1.6E-01	1.5E-01	1.4E-01	1.6E-01	8.9E-03	1.5E-01	1.4E-02	1.6E-01	2.4E-02	1.5E-01	1.2E-02	1.0	0.858		1.0	0.854		1.0	0.625		1.0	0.811		1.0	0.658	
5'-Deoxy-5'-methylthioadenosine	2.3E-04	2.3E-04	2.4E-04	2.3E-04	2.5E-04	2.1E-04	3.0E-04	2.7E-04	2.6E-04	2.8E-04	2.4E-04	2.6E-04	2.3E-04	4.7E-06	2.3E-04	1.9E-05	2.8E-04	1.8E-05	2.6E-04	2.1E-05	1.0	0.745		1.2	0.049	*	1.1	0.159		1.1	0.140		0.9	0.383	
Taurine	1.5E-01	1.6E-01	1.6E-01	1.5E-01	1.6E-01	1.5E-01	1.8E-01	1.6E-01	1.4E-01	1.1E-01	1.5E-01	1.4E-01	1.6E-01	2.8E-03	1.5E-01	8.0E-03	1.6E-01	1.8E-02	1.3E-01	1.7E-02	1.0	0.594		1.0	0.798		0.8	0.128		0.9	0.149		0.8	0.129	
Lys	7.6E-02	1.1E-01	1.0E-01	8.2E-02	1.1E-01	9.1E-02	1.3E-01	1.1E-01	1.4E-01	1.1E-01	1.0E-01	1.4E-01	9.7E-02	1.9E-02	9.4E-02	1.4E-02	1.3E-01	1.6E-02	1.2E-01	1.7E-02	1.0	0.881		1.3	0.117		1.2	0.229		1.2	0.149		0.9	0.591	
Putrescine	2.7E-04	4.2E-04	8.6E-04	5.4E-04	6.7E-04	3.0E-04	1.5E-03	1.2E-03	1.6E-03	1.8E-03	1.5E-03	1.0E-03	5.2E-04	3.1E-04	5.0E-04	1.8E-04	1.5E-03	2.3E-04	1.5E-03	4.2E-04	1.0	0.954		2.8	0.016	*	2.8	0.039	*	2.9	0.042	*	1.0	0.984	
Lauric acid	1.9E-03	2.0E-03	2.1E-03	1.8E-03	2.1E-03	1.9E-03	2.0E-03	1.9E-03	1.7E-03	2.0E-03	1.8E-03	1.4E-03	2.0E-03	1.1E-04	2.0E-03	1.4E-04	1.9E-03	2.0E-04	1.8E-03	2.9E-04	1.0	0.624		0.9	0.388		0.9	0.262		0.9	0.358		0.9	0.571	
Ectoine	1.4E-03	1.3E-03	9.3E-04	1.2E-03	1.0E-03	1.3E-03	1.6E-03	1.5E-03	1.5E-03	1.8E-03	1.4E-03	2.6E-03	1.2E-03	2.6E-04	1.2E-03	1.8E-04	1.5E-03	8.0E-05	1.9E-03	6.2E-04	1.0	0.846		1.3	0.154		1.6	0.174		1.6	0.163		1.3	0.386	
5-Hydroxylysine	7.0E-05	7.7E-05	8.8E-05	7.7E-05	8.4E-05	6.6E-05	1.4E-04	1.2E-04	1.3E-04	1.0E-04	9.1E-05	1.2E-04	7.8E-05	9.0E-06	7.6E-05	8.8E-06	1.3E-04	8.7E-06	1.1E-04	1.7E-05	1.0	0.733		1.6	0.002	**	1.3	0.087		1.4	0.071		0.8	0.135	
Citric acid	6.1E-03	6.5E-03	5.1E-03	5.3E-03	5.7E-03	6.1E-03	7.1E-03	6.6E-03	5.8E-03	7.9E-03	6.5E-03	8.1E-03	5.9E-03	7.4E-04	5.7E-03	4.1E-04	6.5E-03	6.6E-04	7.5E-03	8.5E-04	1.0	0.708		1.1	0.350		1.3	0.070		1.3	0.047	*	1.2	0.186	
Phe	3.8E-02	3.8E-02	4.6E-02	4.2E-02	3.8E-02	3.7E-02	4.1E-02	4.0E-02	4.6E-02	4.3E-02	3.8E-02	4.5E-02	4.0E-02	4.7E-03	3.9E-02	2.3E-03	4.2E-02	3.1E-03	4.2E-02	4.0E-03	1.0	0.630		1.0	0.654		1.0	0.681		1.1	0.310		1.0	0.995	
Galacturonic acid	N.D.	3.8E-04	3.5E-04	N.D.	N.D.	3.5E-04	N.D.	N.D.	N.D.	6.3E-04	4.3E-04	N.D.	3.6E-04	2.3E-05	3.5E-04	N.A.	N.D.	N.A.	5.3E-04	1.4E-04	1.0	N.A.		<1	N.A.		1.5	0.330		1.5	N.A.		1<	N.A.	
AMP	7.4E-02	6.4E-02	6.9E-02	6.2E-02	6.3E-02	7.1E-02	7.5E-02	7.3E-02	6.8E-02	7.7E-02	8.1E-02	8.7E-02	6.9E-02	5.1E-03	6.6E-02	4.7E-03	7.2E-02	3.6E-03	8.2E-02	5.3E-03	1.0	0.474		1.0	0.427		1.2	0.037	*	1.2	0.017	*	1.1	0.065	
O-Acetylcarnitine	4.8E-02	4.5E-02	4.5E-02	4.6E-02	3.7E-02	4.8E-02	4.7E-02	3.9E-02	4.3E-02	4.0E-02	4.0E-02	4.2E-02	4.6E-02	2.0E-03	4.4E-02	5.7E-03	4.3E-02	4.0E-03	4.1E-02	1.1E-03	0.9	0.567		0.9	0.324		0.9	0.027	*	0.9	0.485		1.0	0.457	
Anserine_divalent	3.0E-04	2.4E-04	3.7E-04	2.0E-04	3.9E-04	2.8E-04	4.2E-04	4.1E-04	3.6E-04	2.7E-04	3.6E-04	3.7E-04	3.1E-04	6.2E-05	2.9E-04	9.3E-05	4.0E-04	3.4E-05	3.3E-04	5.5E-05	0.9	0.821		1.3	0.112		1.1	0.606		1.1	0.540		0.8	0.178	
N'-Methyl-4-pyridone-5-	3.1E-04	2.5E-04	3.2E-04	2.8E-04	N.D.	2.8E-04	N.D.	3.7E-04	3.4E-04	3.5E-04	3.2E-04	3.6E-04	3.0E-04	3.8E-05	2.8E-04	1.3E-06	3.5E-04	1.9E-05	3.4E-04	1.8E-05	0.9	0.510		1.2	0.114		1.1	0.169		1.2	0.028	*	1.0	0.531	
Nicotinamide	4.6E-02	5.1E-02	7.3E-02	5.1E-02	5.4E-02	5.5E-02	5.5E-02	5.6E-02	5.1E-02	6.1E-02	6.2E-02	7.5E-02	5.7E-02	1.4E-02	5.3E-02	2.5E-03	5.4E-02	2.5E-03	6.6E-02	7.4E-03	0.9	0.727		1.0	0.770		1.2	0.392		1.2	0.086		1.2	0.093	
Glycerol	6.8E-02	7.6E-02	1.2E-01	7.4E-02	6.9E-02	1.0E-01	1.1E-01	7.1E-02	9.9E-02	7.1E-02	5.9E-02	6.5E-02	8.7E-02	2.6E-02	8.1E-02	1.7E-02	9.4E-02	2.1E-02	6.5E-02	6.2E-03	0.9	0.784		1.1	0.721		0.7	0.275		0.8	0.237		0.7	0.131	
Trp	8.3E-03	8.2E-03	9.6E-03	8.8E-03	7.7E-03	8.0E-03	8.8E-03	9.2E-03	1.0E-02	9.5E-03	8.3E-03	1.1E-02	8.7E-03	7.9E-04	8.2E-03	5.4E-04	9.4E-03	7.4E-04	9.5E-03	1.1E-03	0.9	0.373		1.1	0.314		1.1	0.395		1.2	0.163		1.0	0.969	
GMP	7.0E-03	6.3E-03	6.6E-03	5.7E-03	6.5E-03	6.4E-03	7.8E-03	7.4E-03	6.8E-03	9.3E-03	9.9E-03	9.4E-03	6.6E-03	3.2E-04	6.2E-03	4.0E-04	7.3E-03	5.2E-04	9.5E-03	3.6E-04	0.9	0.218		1.1	0.134		1.4	5.3E-04	***	1.5	4.6E-04	***	1.3	0.005	**
Leu	1.2E-01	1.3E-01	1.4E-01	1.2E-01	1.2E-01	1.2E-01	1.6E-01	1.3E-01	1.6E-01	1.4E-01	1.2E-01	1.4E-01	1.3E-01	1.1E-02	1.2E-01	1.9E-03	1.5E-01	1.7E-02	1.3E-01	1.3E-02	0.9	0.293		1.1	0.233		1.0	0.942		1.1	0.312		0.9	0.263	
Pyridoxamine 5'-phosphate	2.1E-03	2.0E-03	1.9E-03	1.8E-03	1.9E-03	1.9E-03	1.8E-03	1.5E-03	1.5E-03	1.4E-03	1.4E-03	1.4E-03	2.0E-03	9.6E-05	1.8E-03	7.2E-05	1.6E-03	1.7E-04	1.4E-03	3.1E-05	0.9	0.121		0.8	0.041	*	0.7	0.005	**	0.8	0.003	**	0.9	0.167	
Adenylosuccinic acid	9.7E-04	1.1E-03	7.3E-04	7.7E-04	1.0E-03	7.5E-04	1.3E-03	1.4E-03	1.4E-03	1.5E-03	1.2E-03	1.5E-03	9.2E-04	1.7E-04	8.5E-04	1.6E-04	1.4E-03	8.9E-05	1.4E-03	2.0E-04	0.9	0.648		1.5	0.026	*	1.5	0.035	*	1.6	0.023	*	1.0	0.816	
GABA	6.3E-03	7.3E-03	5.0E-03	5.5E-03	6.8E-03	4.9E-03	7.9E-03	7.4E-03	8.2E-03	9.2E-03	5.9E-03	9.2E-03	6.2E-03	1.1E-03	5.7E-03	9.3E-04	7.8E-03	4.3E-04	8.1E-03	1.9E-03	0.9	0.628		1.3	0.113		1.3	0.231		1.4	0.157		1.0	0.864	
5-Oxoproline	7.0E-04	7.6E-04	8.8E-04	7.2E-04	7.1E-04	7.4E-04	1.1E-03	8.7E-04	9.0E-04	7.9E-04	6.5E-04	9.6E-04	7.8E-04	8.9E-05	7.2E-04	1.5E-05	9.5E-04	1.2E-04	8.0E-04	1.5E-04	0.9	0.386		1.2	0.116		1.0	0.865		1.1	0.485		0.8	0.247	
Creatine	3.8E-02	3.5E-02	3.8E-02	3.0E-02	3.6E-02	3.6E-02	4.1E-02	3.5E-02	2.8E-02	3.9E-02	3.9E-02	4.4E-02	3.7E-02	1.8E-03	3.4E-02	3.5E-03	3.5E-02	6.6E-03	4.1E-02	2.7E-03	0.9	0.315		0.9	0.659		1.1	0.108		1.2	0.060		1.2	0.250	
γ-Glu-Cys	1.0E-03	1.2E-03	1.0E-03	8.5E-04	1.2E-03	9.3E-04	1.2E-03	1.1E-03	1.1E-03	1.0E-03	8.3E-04	1.0E-03	1.1E-03	7.8E-05	9.9E-04	1.7E-04	1.1E-03	8.4E-05	9.6E-04	1.2E-04	0.9	0.525		1.1	0.385		0.9	0.285		1.0	0.849		0.9	0.125	
3'-Dephospho CoA	3.4E-04	2.7E-04	3.3E-04	2.3E-04	3.0E-04	3.5E-04	2.2E-04	3.0E-04	N.D.	3.7E-04	3.0E-04	N.D.	3.2E-04	4.0E-05	2.9E-04	5.9E-05	2.6E-04	5.8E-05	3.3E-04	4.8E-05	0.9	0.580		0.8	0.380		1.1	0.731		1.1	0.461		1.3	0.314	
2,3-Diaminopropionic acid	9.0E-05	N.D.	N.D.	N.D.	8.9E-05	7.7E-05	1.0E-04	N.D.	N.D.	N.D.	N.D.	N.D.	9.0E-05	N.A.	8.3E-05	8.7E-06	1.0E-04	N.A.	N.D.	N.A.	0.9	N.A.		1.1	N.A.		<1	N.A.		<1	N.A.		<1	N.A.	
4-Amino-3-hydroxybutyric acid	2.2E-04	1.8E-04	1.7E-04	1.8E-04	1.8E-04	1.8E-04	2.1E-04	2.2E-04	2.0E-04	1.8E-04	1.8E-04	1.9E-04	1.9E-04	2.3E-05	1.8E-04	1.7E-06	2.1E-04	8.1E-06	1.9E-04	3.7E-06	0.9	0.367		1.1	0.301		1.0	0.665		1.0	0.039	*	0.9	0.020	*
1-Pyrroline-5-carboxylic acid	6.5E-04	6.1E-04	6.3E-04	4.3E-04	6.0E-04	7.1E-04	8.0E-04	8.5E-04	1.3E-03	1.2E-03	6.7E-04	1.0E-03	6.3E-04	2.0E-05	5.8E-04	1.4E-04	9.8E-04	2.7E-04	9.8E-04	2.8E-04	0.9	0.588		1.6	0.149		1.6	0.164		1.7	0.116		1.0	0.978	
Guanidinosuccinic acid	5.5E-03	3.0E-03	3.3E-03	3.5E-03	3.2E-03	4.1E-03	3.5E-03	2.7E-03	3.2E-03	3.5E-03	2.1E-03	2.9E-03	3.9E-03	1.3E-03	3.6E-03	4.5E-04	3.2E-03	3.8E-04	2.9E-03	7.0E-04	0.9	0.725		0.8	0.436		0.7	0.317		0.8	0.217		0.9	0.569	
γ-Butyrobetaine	6.9E-03	6.9E-03	6.8E-03	6.3E-03	6.3E-03	6.2E-03	8.4E-03	6.9E-03	7.6E-03	8.1E-03	8.0E-03	8.1E-03	6.8E-03	6.8E-05	6.3E-03	1.9E-05	7.7E-03	7.5E-04	8.1E-03	7.6E-05	0.9	0.003	**	1.1	0.202		1.2	3.5E-05	***	1.3	2.9E-04	***	1.1	0.446	
Ethanolamine	1.9E-03	1.7E-03	2.0E-03	2.1E-03	1.5E-03	1.5E-03	1.6E-03	1.8E-03	1.3E-03	1.4E-03	1.4E-03	2.1E-03	1.9E-03	1.3E-04	1.7E-03	3.3E-04	1.6E-03	2.3E-04	1.6E-03	3.8E-04	0.9	0.504		0.8	0.137		0.9	0.405		1.0	0.803		1.0	0.825	
UDP	5.5E-03	5.0E-03	3.7E-03	4.5E-03	4.0E-03	4.4E-03	5.3E-03	3.8E-03	5.6E-03	2.6E-03	2.3E-03	2.3E-03	4.7E-03	8.9E-04	4.3E-03	2.6E-04	4.9E-03	9.3E-04	2.4E-03	1.6E-04	0.9	0.519		1.0	0.802		0.5	0.042	*	0.6	0.001	**	0.5	0.040	*
Lactic acid	5.9E-01	6.2E-01	6.3E-01	5.5E-01	5.2E-01	6.1E-01	6.8E-01	5.9E-01	6.5E-01	7.0E-01	7.0E-01	6.9E-01	6.1E-01	1.9E-02	5.6E-01	5.0E-02	6.4E-01	4.2E-02	7.0E-01	7.0E-03	0.9	0.199		1.0	0.412		1.1	0.009	**	1.2	0.038	*	1.1	0.127	
Carnitine	7.1E-02	7.3E-02	6.4E-02	6.1E-02	5.8E-02	7.0E-02	5.4E-02	4.7E-02	6.6E-02	5.7E-02	5.4E-02	5.8E-02	6.9E-02	4.7E-03	6.3E-02	6.4E-03	5.6E-02	9.8E-03	5.6E-02	2.2E-03	0.9	0.264		0.8	0.125		0.8	0.027	*	0.9	0.209		1.0	0.897	
5-Aminovaleric acid	1.5E-02	1.5E-02	1.3E-02	1.5E-02	1.2E-02	1.3E-02	2.0E-02	1.7E-02	1.7E-02	1.7E-02	1.5E-02	1.7E-02	1.4E-02	1.6E-03	1.3E-02	1.7E-03	1.8E-02	1.7E-03	1.7E-02	1.0E-03	0.9	0.390		1.3	0.044	*	1.1	0.133		1.3	0.049	*	0.9	0.219	
XC0016	1.7E-03	1.7E-03	1.2E-03	1.3E-03	1.2E-03	1.6E-03	1.6E-03	1.8E-03	1.3E-03	1.6E-03	1.4E-03	2.3E-03	1.5E-03	3.0E-04	1.4E-03	2.0E-04	1.6E-03	2.3E-04	1.7E-03	4.7E-04	0.9	0.557		1.1	0.704		1.2	0.521		1.3	0.312		1.1	0.676	
Uridine	3.2E-03	3.8E-03	5.2E-03	3.7E-03	3.7E-03	3.7E-03	3.8E-03	4.4E-03	4.3E-03	3.8E-03	4.6E-03	4.8E-03	4.1E-03	1.1E-03	3.7E-03	4.2E-05	4.2E-03	2.8E-04	4.4E-03	5.5E-04	0.9	0.607		1.0	0.901		1.1	0.671		1.2	0.161		1.1	0.557	
Cytidine	2.8E-04	2.5E-04	2.9E-04	2.2E-04	2.3E-04	2.8E-04	2.9E-04	2.7E-04	3.0E-04	2.4E-04	2.5E-04	2.5E-04	2.7E-04	2.3E-05	2.5E-04	3.3E-05	2.9E-04	1.3E-05	2.5E-04	8.0E-06	0.9	0.340		1.1	0.343		0.9	0.184		1.0	0.987		0.9	0.014	*
Gly-Leu	1.3E-03	1.2E-03	1.1E-03	9.6E-04	1.1E-03	1.2E-03	1.1E-03	8.8E-04	7.2E-04	6.1E-04	7.5E-04	6.6E-04	1.2E-03	1.1E-04	1.1E-03	1.2E-04	8.9E-04	1.9E-04	6.7E-04	7.2E-05	0.9	0.262		0.7	0.080		0.6	0.004	**	0.6	0.011	*	0.8	0.167	
FAD_divalent	1.8E-03	1.6E-03	1.6E-03	1.4E-03	1.5E-03	1.5E-03	1.4E-03	1.2E-03	1.1E-03	8.7E-04	1.2E-03	1.1E-03	1.7E-03	1.1E-04	1.5E-03	6.8E-05	1.3E-03	1.8E-04	1.1E-03	1.8E-04	0.9	0.090		0.7	0.036	*	0.6	0.012	*	0.7	0.038	*	0.9	0.281	
Taurocyamine	5.7E-04	6.3E-04	5.9E-04	5.5E-04	4.6E-04	6.0E-04	6.7E-04	5.6E-04	5.7E-04	4.2E-04	4.5E-04	4.4E-04	6.0E-04	2.9E-05	5.4E-04	6.7E-05	6.0E-04	6.5E-05	4.4E-04	1.9E-05	0.9	0.253		1.0	0.980		0.7	0.002	**	0.8	0.114		0.7	0.041	*
S-Adenosylhomocystein	4.4E-03	4.7E-03	5.1E-03	4.6E-03	4.3E-03	3.9E-03	4.2E-03	4.1E-03	4.2E-03	4.5E-03	4.0E-03	4.7E-03	4.8E-03	3.6E-04	4.3E-03	3.3E-04	4.2E-03	8.9E-05	4.4E-03	3.5E-04	0.9	0.152		0.9	0.100		0.9	0.273		1.0	0.651		1.1	0.388	
1-Methyl-4-imidazoleacetic acid	8.5E-04	4.3E-04	4.7E-04	3.6E-04	6.3E-04	5.8E-04	6.3E-04	8.0E-04	5.0E-04	5.7E-04	4.7E-04	7.2E-04	5.8E-04	2.3E-04	5.2E-04	1.4E-04	6.4E-04	1.5E-04	5.9E-04	1.3E-04	0.9	0.714		1.1	0.723		1.0	0.979		1.1	0.577		0.9	0.642	
XC0071	5.8E-05	7.9E-05	1.0E-04	5.8E-05	7.0E-05	8.5E-05	8.2E-05	7.4E-05	5.6E-05	7.9E-05	8.5E-05	1.0E-04	8.0E-05	2.4E-05	7.1E-05	1.4E-05	7.1E-05	1.3E-05	8.9E-05	1.3E-05	0.9	0.589		0.9	0.592		1.1	0.619		1.3	0.168		1.3	0.166	
Urea	4.8E-01	4.3E-01	4.2E-01	3.8E-01	3.8E-01	4.1E-01	5.8E-01	5.2E-01	5.2E-01	4.7E-01	4.2E-01	5.1E-01	4.4E-01	3.2E-02	3.9E-01	1.8E-02	5.4E-01	3.3E-02	4.7E-01	4.2E-02	0.9	0.086		1.2	0.023	*	1.1	0.470		1.2	0.069		0.9	0.088	
Ile	7.4E-02	7.9E-02	8.3E-02	6.8E-02	6.8E-02	7.2E-02	9.7E-02	8.1E-02	9.5E-02	8.1E-02	6.9E-02	8.5E-02	7.9E-02	4.4E-03	6.9E-02	2.1E-03	9.1E-02	8.7E-03	7.8E-02	8.1E-03	0.9	0.047	*	1.2	0.119		1.0	0.928		1.1	0.193		0.9	0.136	
SDMA	2.0E-04	2.1E-04	1.7E-04	1.6E-04	1.7E-04	1.8E-04	1.5E-04	1.9E-04	1.6E-04	1.4E-04	1.5E-04	1.7E-04	1.9E-04	2.1E-05	1.7E-04	8.3E-06	1.7E-04	1.8E-05	1.5E-04	1.8E-05	0.9	0.189		0.9	0.189		0.8	0.072		0.9	0.257		0.9	0.398	
Imidazole-4-acetic acid	2.3E-04	2.3E-04	1.8E-04	1.8E-04	1.8E-04	2.0E-04	1.9E-04	2.6E-04	2.3E-04	2.3E-04	2.2E-04	2.3E-04	2.1E-04	3.1E-05	1.9E-04	9.1E-06	2.3E-04	3.5E-05	2.3E-04	7.6E-06	0.9	0.278		1.1	0.558		1.1	0.499		1.2	0.004	**	1.0	0.914	

Uracil	5.3E-04	6.4E-04	8.5E-04	5.8E-04	6.1E-04	5.8E-04	5.8E-04	8.0E-04	6.5E-04	5.5E-04	7.1E-04	7.1E-04	6.7E-04	1.6E-04	5.9E-04	1.8E-05	6.8E-04	1.1E-04	6.6E-04	9.5E-05	0.9	0.466		1.0	0.969		1.0	0.874		1.1	0.349		1.0	0.798	
Ethanolamine phosphate	8.3E-03	8.1E-03	7.9E-03	7.1E-03	7.1E-03	7.0E-03	7.8E-03	8.3E-03	6.2E-03	6.0E-03	6.6E-03	6.6E-03	8.1E-03	2.3E-04	7.1E-03	6.1E-05	7.4E-03	1.1E-03	6.4E-03	3.3E-04	0.9	0.011	*	0.9	0.381		0.8	0.003	**	0.9	0.072		0.9	0.259	
Argininosuccinic acid	1.6E-03	2.1E-03	2.1E-03	1.4E-03	2.1E-03	1.6E-03	2.1E-03	1.4E-03	1.8E-03	1.9E-03	1.4E-03	1.8E-03	2.0E-03	2.8E-04	1.7E-03	3.4E-04	1.8E-03	3.5E-04	1.7E-03	2.4E-04	0.9	0.360		0.9	0.525		0.9	0.303		1.0	0.975		1.0	0.779	
Ala-Ala	1.1E-03	1.3E-03	1.4E-03	1.3E-03	1.1E-03	9.6E-04	1.1E-03	7.9E-04	7.2E-04	7.5E-04	7.8E-04	9.4E-04	1.3E-03	1.3E-04	1.1E-03	1.5E-04	8.6E-04	1.7E-04	8.3E-04	1.0E-04	0.9	0.196		0.7	0.031	*	0.6	0.010	**	0.7	0.061		1.0	0.810	
N^6-Acetyllysine	4.9E-04	5.5E-04	4.6E-04	4.2E-04	4.6E-04	4.1E-04	6.0E-04	6.1E-04	5.3E-04	6.1E-04	4.8E-04	6.9E-04	5.0E-04	4.7E-05	4.3E-04	2.7E-05	5.8E-04	4.5E-05	6.0E-04	1.1E-04	0.9	0.111		1.2	0.087		1.2	0.248		1.4	0.106		1.0	0.855	
Betonicine	4.7E-04	5.5E-04	4.1E-04	5.2E-04	3.7E-04	3.4E-04	5.5E-04	6.0E-04	5.3E-04	5.5E-04	5.2E-04	6.0E-04	4.8E-04	7.1E-05	4.1E-04	9.7E-05	5.6E-04	4.0E-05	5.6E-04	3.8E-05	0.9	0.398		1.2	0.167		1.2	0.182		1.4	0.105		1.0	0.901	
N^5-Ethylglutamine	7.9E-04	7.4E-04	6.1E-04	6.0E-04	6.5E-04	6.0E-04	8.5E-04	9.1E-04	8.3E-04	8.0E-04	7.1E-04	8.0E-04	7.2E-04	9.2E-05	6.2E-04	2.5E-05	8.7E-04	4.2E-05	7.7E-04	5.0E-05	0.9	0.194		1.2	0.087		1.1	0.448		1.2	0.019	*	0.9	0.063	
Cystathionine	7.6E-03	1.0E-02	7.2E-03	6.4E-03	8.9E-03	6.1E-03	1.6E-02	1.0E-02	1.7E-02	1.5E-02	1.1E-02	1.6E-02	8.3E-03	1.6E-03	7.2E-03	1.5E-03	1.4E-02	3.9E-03	1.4E-02	2.8E-03	0.9	0.414		1.7	0.099		1.6	0.058		1.9	0.036	*	0.9	0.807	
Ergothioneine	4.8E-02	5.1E-02	4.8E-02	2.8E-02	4.6E-02	5.3E-02	4.1E-02	3.0E-02	3.8E-02	4.0E-02	3.6E-02	3.9E-02	4.9E-02	2.0E-03	4.2E-02	1.3E-02	3.6E-02	5.4E-03	3.8E-02	2.5E-03	0.9	0.448		0.7	0.043	*	0.8	0.005	**	0.9	0.668		1.1	0.588	
Citrulline	9.8E-03	1.4E-02	1.7E-02	1.1E-02	1.3E-02	1.2E-02	1.4E-02	1.0E-02	1.2E-02	1.1E-02	1.1E-02	1.5E-02	1.4E-02	3.7E-03	1.2E-02	9.8E-04	1.2E-02	2.0E-03	1.2E-02	2.7E-03	0.9	0.466		0.9	0.522		0.9	0.617		1.0	0.788		1.0	0.879	
Stachydrine	3.6E-02	3.4E-02	3.1E-02	3.5E-02	2.5E-02	2.7E-02	4.0E-02	3.8E-02	3.5E-02	3.5E-02	3.2E-02	3.7E-02	3.4E-02	2.7E-03	2.9E-02	5.3E-03	3.8E-02	2.5E-03	3.4E-02	2.5E-03	0.9	0.233		1.1	0.160		1.0	0.854		1.2	0.205		0.9	0.189	
Gly-Gly	4.4E-04	4.8E-04	5.1E-04	3.9E-04	4.1E-04	4.2E-04	4.1E-04	3.4E-04	3.8E-04	4.0E-04	4.4E-04	5.2E-04	4.7E-04	3.6E-05	4.0E-04	1.6E-05	3.8E-04	3.1E-05	4.5E-04	6.2E-05	0.8	0.058		0.8	0.024	*	1.0	0.625		1.1	0.302		1.2	0.156	
CMP-N-acetylneuraminate	3.0E-03	3.4E-03	2.7E-03	2.8E-03	2.5E-03	2.4E-03	2.3E-03	2.7E-03	2.0E-03	2.1E-03	1.9E-03	4.2E-03	3.0E-03	3.3E-04	2.6E-03	2.2E-04	2.4E-03	3.6E-04	2.7E-03	1.3E-03	0.8	0.130		0.8	0.083		0.9	0.733		1.1	0.852		1.2	0.685	
Adenine	1.2E-03	1.9E-03	1.3E-03	1.6E-03	1.0E-03	1.1E-03	1.0E-03	1.7E-03	1.2E-03	1.3E-03	1.3E-03	2.1E-03	1.5E-03	3.8E-04	1.2E-03	2.9E-04	1.3E-03	3.5E-04	1.6E-03	4.7E-04	0.8	0.462		0.9	0.639		1.1	0.748		1.3	0.351		1.2	0.471	
Guanidoacetic acid	2.5E-03	2.2E-03	1.8E-03	1.7E-03	2.2E-03	1.5E-03	2.3E-03	2.7E-03	1.9E-03	2.0E-03	2.1E-03	2.9E-03	2.2E-03	3.2E-04	1.8E-03	3.4E-04	2.3E-03	4.0E-04	2.3E-03	5.0E-04	0.8	0.260		1.1	0.668		1.1	0.686		1.3	0.231		1.0	0.970	
3-Methylhistidine	2.9E-03	2.8E-03	2.8E-03	2.4E-03	2.4E-03	2.3E-03	3.8E-03	3.5E-03	3.7E-03	3.1E-03	1.9E-03	3.3E-03	2.8E-03	6.1E-05	2.4E-03	5.7E-05	3.7E-03	1.5E-04	2.8E-03	7.4E-04	0.8	6.5E-04	***	1.3	0.004	**	1.0	0.923		1.2	0.428		0.7	0.158	
N-Methylalanine	2.0E-04	2.2E-04	N.D.	1.8E-04	1.6E-04	1.7E-04	2.7E-04	2.6E-04	2.5E-04	2.9E-04	2.2E-04	2.2E-04	2.1E-04	1.5E-05	1.7E-04	8.9E-06	2.6E-04	6.2E-06	2.4E-04	4.5E-05	0.8	0.139		1.3	0.099		1.2	0.288		1.4	0.103		0.9	0.610	
Pro	8.5E-02	9.8E-02	7.9E-02	6.7E-02	8.3E-02	6.9E-02	1.1E-01	9.0E-02	1.1E-01	1.0E-01	8.2E-02	1.2E-01	8.8E-02	9.8E-03	7.3E-02	8.4E-03	1.0E-01	1.2E-02	1.0E-01	1.6E-02	0.8	0.123		1.2	0.139		1.1	0.345		1.4	0.086		1.0	0.704	
Val	1.1E-01	1.1E-01	1.1E-01	8.6E-02	9.1E-02	9.6E-02	1.4E-01	1.2E-01	1.3E-01	1.1E-01	9.5E-02	1.2E-01	1.1E-01	1.7E-03	9.1E-02	5.3E-03	1.3E-01	1.3E-02	1.1E-01	1.3E-02	0.8	0.018	*	1.2	0.117		1.0	0.859		1.2	0.142		0.8	0.122	
β-Ala-Lys	5.4E-04	7.2E-04	8.7E-04	6.7E-04	5.6E-04	5.3E-04	6.4E-04	4.3E-04	4.9E-04	5.5E-04	5.6E-04	6.5E-04	7.1E-04	1.7E-04	5.9E-04	7.1E-05	5.2E-04	1.1E-04	5.9E-04	5.5E-05	0.8	0.330		0.7	0.187		0.8	0.324		1.0	0.983		1.1	0.432	
Succinic acid	8.0E-02	8.5E-02	6.5E-02	6.5E-02	5.7E-02	6.8E-02	8.0E-02	8.8E-02	7.5E-02	9.1E-02	7.9E-02	8.2E-02	7.7E-02	1.1E-02	6.3E-02	5.9E-03	8.1E-02	6.6E-03	8.4E-02	6.1E-03	0.8	0.141		1.1	0.577		1.1	0.383		1.3	0.013	*	1.0	0.634	
3-Aminobutyric acid	1.2E-03	7.6E-04	6.7E-04	7.5E-04	6.7E-04	7.2E-04	1.0E-03	8.4E-04	9.0E-04	8.4E-04	6.8E-04	9.8E-04	8.7E-04	2.7E-04	7.1E-04	4.2E-05	9.2E-04	9.7E-05	8.3E-04	1.5E-04	0.8	0.422		1.1	0.777		1.0	0.844		1.2	0.305		0.9	0.439	
Carboxymethyllysine	9.5E-05	8.3E-05	1.3E-04	8.8E-05	5.9E-05	1.0E-04	9.1E-05	1.1E-04	1.0E-04	1.0E-04	8.2E-05	1.1E-04	1.0E-04	2.2E-05	8.2E-05	2.1E-05	1.0E-04	7.8E-06	1.0E-04	1.6E-05	0.8	0.337		1.0	0.937		1.0	0.929		1.2	0.322		1.0	0.976	
Homoserine	5.2E-04	6.3E-04	4.5E-04	4.2E-04	4.7E-04	4.1E-04	7.2E-04	6.0E-04	6.4E-04	6.4E-04	4.9E-04	6.5E-04	5.3E-04	9.2E-05	4.3E-04	3.1E-05	6.5E-04	6.0E-05	5.9E-04	8.8E-05	0.8	0.193		1.2	0.139		1.1	0.446		1.4	0.073		0.9	0.396	
Pyridoxal	1.7E-04	2.2E-04	1.7E-04	1.6E-04	1.3E-04	1.6E-04	1.6E-04	2.1E-04	1.4E-04	1.5E-04	1.6E-04	1.8E-04	1.8E-04	3.1E-05	1.5E-04	1.7E-05	1.7E-04	3.5E-05	1.6E-04	1.7E-05	0.8	0.182		0.9	0.599		0.9	0.296		1.1	0.546		0.9	0.688	
Hypotaurine	4.6E-03	3.7E-03	3.5E-03	3.5E-03	3.3E-03	2.8E-03	5.8E-03	4.9E-03	5.3E-03	5.9E-03	3.9E-03	5.1E-03	3.9E-03	5.8E-04	3.2E-03	3.3E-04	5.3E-03	4.5E-04	5.0E-03	1.0E-03	0.8	0.146		1.4	0.033	*	1.3	0.213		1.6	0.080		0.9	0.618	
Sarcosine	4.1E-02	3.8E-02	3.2E-02	3.2E-02	3.0E-02	2.8E-02	4.2E-02	4.0E-02	4.0E-02	4.0E-02	3.9E-02	4.7E-02	3.7E-02	5.0E-03	3.0E-02	2.3E-03	4.1E-02	1.4E-03	4.2E-02	4.7E-03	0.8	0.119		1.1	0.357		1.1	0.276		1.4	0.030	*	1.0	0.629	
N-Acetyllysine	2.2E-03	2.2E-03	2.5E-03	1.8E-03	1.9E-03	1.8E-03	2.0E-03	2.1E-03	2.2E-03	1.7E-03	1.7E-03	2.4E-03	2.3E-03	1.4E-04	1.9E-03	5.7E-05	2.1E-03	8.5E-05	1.9E-03	3.7E-04	0.8	0.021	*	0.9	0.155		0.8	0.236		1.1	0.704		0.9	0.503	
β-Ala	2.4E-02	2.5E-02	2.0E-02	1.8E-02	2.0E-02	1.8E-02	2.5E-02	2.0E-02	2.5E-02	2.4E-02	1.6E-02	2.6E-02	2.3E-02	2.7E-03	1.8E-02	9.8E-04	2.4E-02	2.8E-03	2.2E-02	5.0E-03	0.8	0.086		1.0	0.809		1.0	0.781		1.2	0.352		0.9	0.667	
Cysteine glutathione disulfide	1.3E-04	2.1E-04	1.7E-04	1.6E-04	1.3E-04	1.2E-04	2.0E-04	1.4E-04	1.4E-04	1.9E-04	2.0E-04	3.6E-04	1.7E-04	3.7E-05	1.4E-04	2.1E-05	1.6E-04	3.4E-05	2.5E-04	9.5E-05	0.8	0.257		0.9	0.735		1.5	0.287		1.8	0.170		1.6	0.240	
N,N-Dimethylglycine	2.6E-02	2.1E-02	2.0E-02	2.0E-02	1.7E-02	1.7E-02	2.2E-02	2.3E-02	2.3E-02	2.4E-02	2.4E-02	2.4E-02	2.2E-02	3.4E-03	1.8E-02	1.5E-03	2.2E-02	6.6E-04	2.4E-02	1.7E-04	0.8	0.137		1.0	0.961		1.1	0.469		1.4	0.018	*	1.1	0.042	*
N-Acetylglutamic acid	7.8E-03	9.3E-03	6.3E-03	5.7E-03	6.7E-03	6.2E-03	9.2E-03	7.2E-03	9.3E-03	8.3E-03	7.0E-03	8.4E-03	7.8E-03	1.5E-03	6.2E-03	4.8E-04	8.6E-03	1.2E-03	7.9E-03	7.6E-04	0.8	0.200		1.1	0.531		1.0	0.936		1.3	0.040	*	0.9	0.465	
Acetoacetamide	1.9E-04	1.7E-04	N.D.	1.6E-04	1.4E-04	1.3E-04	2.3E-04	2.2E-04	1.7E-04	2.0E-04	1.9E-04	3.4E-04	1.8E-04	1.3E-05	1.4E-04	1.9E-05	2.1E-04	3.0E-05	2.4E-04	8.4E-05	0.8	0.089		1.2	0.239		1.4	0.316		1.7	0.167		1.2	0.554	
N-Acetylornithine	5.3E-04	5.2E-04	4.8E-04	4.5E-04	3.9E-04	3.7E-04	5.8E-04	4.6E-04	4.1E-04	4.0E-04	4.2E-04	4.7E-04	5.1E-04	2.3E-05	4.0E-04	3.8E-05	4.8E-04	9.0E-05	4.3E-04	3.9E-05	0.8	0.022	*	0.9	0.663		0.8	0.051		1.1	0.439		0.9	0.428	
ADP-ribose	4.1E-03	4.4E-03	6.6E-03	3.3E-03	4.8E-03	3.8E-03	4.8E-03	4.0E-03	2.9E-03	4.9E-03	5.7E-03	6.3E-03	5.0E-03	1.4E-03	4.0E-03	7.5E-04	3.9E-03	9.4E-04	5.6E-03	6.8E-04	0.8	0.324		0.8	0.306		1.1	0.543		1.4	0.049	*	1.4	0.067	
Gly-Asp	1.1E-04	1.3E-04	1.7E-04	1.3E-04	7.8E-05	1.2E-04	8.6E-05	7.7E-05	8.4E-05	1.0E-04	1.1E-04	1.1E-04	1.4E-04	3.0E-05	1.1E-04	2.9E-05	8.2E-05	4.4E-06	1.1E-04	5.7E-06	0.8	0.292		0.6	0.079		0.8	0.209		1.0	0.897		1.3	0.004	**
Malonylcarnitine	2.6E-04	2.7E-04	2.3E-04	2.3E-04	1.9E-04	1.7E-04	2.1E-04	1.9E-04	2.0E-04	1.8E-04	1.9E-04	2.0E-04	2.5E-04	1.9E-05	2.0E-04	2.8E-05	2.0E-04	9.5E-06	1.9E-04	8.3E-06	0.8	0.061		0.8	0.024	*	0.7	0.017	*	1.0	0.614		1.0	0.255	
Isobutyryl CoA_divalent	4.2E-04	6.4E-04	7.1E-04	4.8E-04	4.7E-04	4.4E-04	1.8E-03	6.0E-04	4.7E-04	9.0E-04	9.1E-04	1.4E-03	5.9E-04	1.5E-04	4.6E-04	2.3E-05	9.7E-04	7.6E-04	1.1E-03	2.6E-04	0.8	0.283		1.6	0.479		1.8	0.067		2.3	0.056		1.1	0.865	
XC0001	1.7E-04	2.1E-04	1.5E-04	9.9E-05	1.7E-04	1.5E-04	3.0E-04	2.4E-04	2.2E-04	2.0E-04	1.8E-04	1.8E-04	1.7E-04	3.3E-05	1.4E-04	3.5E-05	2.5E-04	4.1E-05	1.9E-04	9.3E-06	0.8	0.249		1.4	0.070		1.1	0.544		1.4	0.115		0.8	0.115	
CMP	8.1E-04	6.7E-04	7.7E-04	5.2E-04	6.5E-04	6.0E-04	8.2E-04	8.3E-04	6.6E-04	8.2E-04	8.1E-04	7.0E-04	7.5E-04	7.0E-05	5.9E-04	6.9E-05	7.7E-04	1.0E-04	7.8E-04	6.9E-05	0.8	0.047	*	1.0	0.798		1.0	0.689		1.3	0.030	*	1.0	0.946	
Tyr-Arg_divalent	9.3E-05	1.2E-04	1.6E-04	1.0E-04	1.0E-04	8.7E-05	7.2E-05	9.9E-05	7.4E-05	8.7E-05	8.0E-05	8.0E-05	1.2E-04	3.3E-05	9.7E-05	8.5E-06	8.2E-05	1.5E-05	8.3E-05	4.3E-06	0.8	0.298		0.7	0.149		0.7	0.165		0.9	0.083		1.0	0.928	
3-Hydroxybutyric acid	1.2E-02	1.3E-02	1.1E-02	8.2E-03	8.8E-03	1.1E-02	2.2E-02	1.3E-02	1.6E-02	1.3E-02	1.2E-02	1.8E-02	1.2E-02	7.7E-04	9.3E-03	1.5E-03	1.7E-02	4.7E-03	1.5E-02	3.4E-03	0.8	0.077		1.4	0.207		1.2	0.320		1.6	0.103		0.9	0.518	
Thr-Asp	6.9E-05	5.7E-05	8.2E-05	5.9E-05	4.9E-05	N.D.	N.D.	N.D.	2.0E-05	3.5E-05	N.D.	3.9E-05	6.9E-05	1.2E-05	5.4E-05	7.6E-06	2.0E-05	N.A.	3.7E-05	3.2E-06	0.8	0.186		0.3	N.A.		0.5	0.036	*	0.7	0.156		1.9	N.A.	
Pipecolic acid	3.8E-03	3.8E-03	2.9E-03	2.8E-03	2.6E-03	2.8E-03	4.6E-03	4.3E-03	3.8E-03	3.9E-03	3.0E-03	4.0E-03	3.5E-03	5.1E-04	2.7E-03	1.3E-04	4.2E-03	4.3E-04	3.6E-03	5.4E-04	0.8	0.110		1.2	0.136		1.0	0.769		1.3	0.093		0.9	0.220	
4-Guanidinobutyric acid	1.7E-02	1.3E-02	1.2E-02	1.0E-02	8.4E-03	1.3E-02	1.8E-02	1.6E-02	1.8E-02	1.7E-02	1.4E-02	1.9E-02	1.4E-02	3.1E-03	1.1E-02	2.4E-03	1.7E-02	1.2E-03	1.7E-02	2.4E-03	0.8	0.230		1.3	0.169		1.2	0.281		1.6	0.035	*	1.0	0.637	
Glutathione (GSSG)_divalent	1.0E-01	1.2E-01	9.1E-02	1.1E-01	6.3E-02	6.8E-02	7.5E-02	1.1E-01	6.5E-02	7.2E-02	7.9E-02	1.4E-01	1.1E-01	1.7E-02	8.0E-02	2.5E-02	8.2E-02	2.2E-02	9.7E-02	3.7E-02	0.8	0.234		0.8	0.223		0.9	0.724		1.2	0.565		1.2	0.599	
Homocitrulline	5.1E-04	4.4E-04	6.9E-04	3.6E-04	4.5E-04	4.2E-04	6.6E-04	6.2E-04	6.4E-04	5.4E-04	4.1E-04	5.0E-04	5.4E-04	1.3E-04	4.1E-04	4.4E-05	6.4E-04	1.7E-05	4.8E-04	6.2E-05	0.8	0.213		1.2	0.320		0.9	0.520		1.2	0.191		0.8	0.041	*
Met	1.3E-02	1.3E-02	1.3E-02	9.3E-03	1.1E-02	9.8E-03	1.9E-02	1.7E-02	1.6E-02	1.4E-02	1.2E-02	1.8E-02	1.3E-02	2.1E-04	9.9E-03	6.5E-04	1.7E-02	1.3E-03	1.5E-02	2.8E-03	0.8	0.008	**	1.3	0.032	*	1.1	0.380		1.5	0.086		0.9	0.307	
NADH	2.3E-03	1.7E-03	1.1E-03	1.1E-03	1.8E-03	9.5E-04	8.9E-04	3.7E-04	2.9E-04	2.4E-04	3.2E-04	2.3E-04	1.7E-03	5.9E-04	1.3E-03	4.5E-04	5.2E-04	3.2E-04	2.7E-04	4.7E-05	0.8	0.387		0.3	0.053		0.2	0.051		0.2	0.058		0.5	0.311	
Thiaproline	6.9E-04	1.1E-03	6.9E-04	7.0E-04	6.3E-04	5.3E-04	5.3E-04	7.0E-04	5.8E-04	7.3E-04	9.0E-04	1.1E-03	8.4E-04	2.5E-04	6.2E-04	8.9E-05	6.0E-04	8.9E-05	9.1E-04	2.0E-04	0.7	0.277		0.7	0.248		1.1	0.695		1.5	0.105		1.5	0.093	
Isovalerylalanine	5.1E-04	4.1E-04	N.D.	2.8E-04	3.6E-04	3.8E-04	7.0E-04	4.8E-04	7.0E-04	6.0E-04	4.2E-04	6.3E-04	4.6E-04	7.6E-05	3.4E-04	5.7E-05	6.3E-04	1.3E-04	5.5E-04	1.1E-04	0.7	0.210		1.4	0.168		1.2	0.365		1.6	0.066		0.9	0.497	
2-Aminobutyric acid	7.9E-03	1.1E-02	6.4E-03	5.0E-03	6.7E-03	7.0E-03	1.4E-02	9.2E-03	9.8E-03	9.1E-03	7.4E-03	1.1E-02	8.4E-03	2.3E-03	6.2E-03	1.1E-03	1.1E-02	2.6E-03	9.1E-03	1.7E-03	0.7	0.237		1.3	0.277		1.1	0.705		1.5	0.076		0.8	0.365	
Creatinine	1.8E-03	2.3E-03	2.1E-03	1.5E-03	1.6E-03	1.4E-03	2.3E-03	2.0E-03	2.1E-03	1.8E-03	1.5E-03	2.0E-03	2.1E-03	2.4E-04	1.5E-03	9.5E-05	2.1E-03	1.6E-04	1.8E-03	2.6E-04	0.7	0.042	*	1.0	0.746		0.9	0.232		1.2	0.209		0.8	0.136	
Ornithine	6.4E-02	8.7E-02	6.3E-02	4.3E-02	5.9E-02	5.3E-02	1.2E-01	8.7E-02	1.0E-01	9.4E-02	7.9E-02	1.0E-01	7.1E-02	1.4E-02	5.2E-02	8.0E-03	1.0E-01	1.4E-02	9.2E-02	1.2E-02	0.7	0.116		1.4	0.057		1.3	0.118		1.8	0.013	*	0.9	0.454	
Kynurenine	2.2E-04	2.9E-04	2.7E-04	2.0E-04	1.9E-04	1.7E-04	3.0E-04	2.3E-04	2.6E-04	2.5E-04	2.5E-04	4.5E-04	2.6E-04	3.5E-05	1.9E-04	1.5E-05	2.6E-04	3.8E-05	3.1E-04	1.2E-04	0.7	0.051		1.0	0.899		1.2	0.518		1.7	0.200		1.2	0.548	
$1H$-Imidazole-4-propionic acid	4.6E-04	3.2E-04	N.D.	3.3E-04	2.4E-04	2.6E-04	3.3E-04	2.7E-04	2.8E-04	2.7E-04	1.0E-04	2.1E-04	3.9E-04	9.9E-05	2.8E-04	4.8E-05	3.0E-04	3.4E-05	2.0E-04	8.4E-05	0.7	0.341		0.8	0.411		0.5	0.158		0.7	0.235		0.7	0.163	
XC0061	1.1E-02	1.3E-02	8.2E-03	7.7E-03	7.0E-03	8.4E-03	7.2E-03	8.5E-03	9.0E-03	7.8E-03	6.5E-03	1.0E-02	1.1E-02	2.3E-03	7.7E-03	7.1E-04	8.2E-03	9.4E-04	8.2E-03	1.9E-03	0.7	0.146		0.8	0.194		0.8	0.217		1.1	0.715		1.0	0.982	
CDP-choline	6.4E-04	7.7E-04	6.3E-04	4.7E-04	5.7E-04	4.3E-04	5.1E-04	6.2E-04	6.0E-04	5.4E-04	5.5E-04	6.4E-04	6.8E-04	7.7E-05	4.9E-04	7.4E-05	5.8E-04	5.8E-05	5.8E-04	5.7E-05	0.7	0.035	*	0.8	0.134		0.8	0.138		1.2	0.179		1.0	0.971	
3-Aminoisobutyric acid	3.8E-03	4.2E-03	2.5E-03	2.2E-03	3.0E-03	2.3E-03	4.9E-03	4.0E-03	6.2E-03	6.4E-03	2.9E-03	6.1E-03	3.5E-03	9.3E-04	2.5E-03	4.3E-04	5.0E-03	1.1E-03	5.1E-03	1.9E-03	0.7	0.195		1.4	0.146		1.5	0.280		2.0	0.134		1.0	0.942	
Saccharopine	1.9E-03	2.0E-03	7.5E-04	5.6E-04	1.2E-03	1.4E-03	2.1E-03	1.5E-03	1.4E-03	3.5E-03	1.4E-03	3.0E-03	1.5E-03	6.9E-04	1.1E-03	4.5E-04	1.7E-03	3.7E-04	2.7E-03	1.1E-03	0.7	0.396		1.1	0.817		1.7	0.215		2.5	0.111		1.6	0.241	
XA0033	2.3E-02	2.2E-02	3.0E-02	2.3E-02	1.4E-02	1.5E-02	2.7E-02	2.3E-02	2.8E-02	2.8E-02	3.1E-02	2.5E-02	2.5E-02	4.8E-03	1.7E-02	4.9E-03	2.6E-02	2.9E-03	2.8E-02	3.2E-03	0.7	0.132		1.1	0.723		1.1	0.410		1.6	0.043	*	1.1	0.498	
3-Ureidopropionic acid	6.1E-04	5.9E-04	6.2E-04	N.D.	4.2E-04	4.2E-04	6.0E-04	5.7E-04	6.1E-04	4.7E-04	N.D.	8.1E-04	6.1E-04	1.5E-05	4.2E-04	1.5E-06	5.9E-04	2.1E-05	6.4E-04	2.4E-04	0.7	0.002	**	1.0	0.443		1.1	0.865		1.5	0.417		1.1	0.820	
Phosphorylcholine	1.1E-01	1.1E-01	1.5E-01	1.2E-01	6.4E-02	7.7E-02	1.4E-01	1.2E-01	1.4E-01	1.4E-01	1.6E-01	1.3E-01	1.3E-01	2.4E-02	8.7E-02	3.0E-02	1.3E-01	1.4E-02	1.4E-01	1.9E-02	0.7	0.159		1.0	0.739		1.1	0.382		1.6	0.063		1.1	0.446	
Gluconolactone	8.4E-03	9.6E-03	7.8E-03	6.0E-03	5.0E-03	6.9E-03	8.6E-03	7.8E-03	8.0E-03	6.9E-03	7.6E-03	8.0E-03	8.6E-03	9.3E-04	6.0E-03	9.6E-04	8.1E-03	4.2E-04	7.5E-03	5.7E-04	0.7	0.026	*	0.9	0.487		0.9	0.164		1.3	0.091		0.9	0.195	
2-Hydroxybutyric acid	3.3E-03	4.2E-03	2.2E-03	1.6E-03	2.6E-03	2.5E-03	5.3E-03	3.1E-03	3.2E-03	3.2E-03	3.0E-03	4.4E-03	3.2E-03	9.6E-04	2.2E-03	5.5E-04	3.9E-03	1.3E-03	3.5E-03	7.9E-04	0.7	0.213		1.2	0.529		1.1	0.704		1.6	0.089		0.9	0.716	
2-Aminoadipic acid	4.9E-03	6.2E-03	2.5E-03	2.7E-03	3.6E-03	3.0E-03	9.9E-03	6.4E-03	7.7E-03	8.4E-03	4.5E-03	7.8E-03	4.5E-03	1.9E-03	3.1E-03	4.7E-04	8.0E-03	1.7E-03	6.9E-03	2.1E-03	0.7	0.326		1.8	0.075		1.5	0.214		2.2	0.082		0.9	0.521	
Trigonelline	7.3E-03	7.0E-03	6.7E-03	5.0E-03	4.4E-03	5.1E-03	9.6E-03	1.0E-02	8.1E-03	8.3E-03	6.7E-03	8.5E-03	7.0E-03	3.1E-04	4.8E-03	4.0E-04	9.2E-03	9.9E-04	7.9E-03	9.7E-04	0.7	0.002	**	1.3	0.049	*	1.1	0.257		1.6	0.020	*	0.9	0.165	
Hydroxyproline	1.1E-02	1.4E-02	8.4E-03	7.6E-03	7.3E-03	7.4E-03	1.4E-02	1.3E-02	1.4E-02	1.3E-02	9.4E-03	1.3E-02	1.1E-02	2.6E-03	7.4E-03	1.7E-04	1.4E-02	5.4E-04	1.2E-02	2.1E-03	0.7	0.142		1.2	0.217		1.1	0.685		1.6	0.067		0.9	0.273	
UMP	1.4E-02	1.4E-02	1.2E-02	8.5E-03	9.3E-03	9.1E-03	1.6E-02	1.3E-02	1.3E-02	1.3E-02	1.4E-02	1.4E-02	1.3E-02	1.2E-03	9.0E-03	4.1E-04	1.4E-02	1.5E-03	1.4E-02	3.1E-04	0.7	0.015	*	1.0	0.702		1.0	0.798		1.5	1.7E-04	***	1.0	0.799	
Gluconic acid	2.2E-02	2.4E-02	2.1E-02	1.5E-02	1.3E-02	1.7E-02	2.2E-02	1.9E-02	2.2E-02	1.8E-02	2.0E-02	2.1E-02	2.2E-02	1.8E-03	1.5E-02	2.2E-03	2.1E-02	1.5E-03	1.9E-02	1.4E-03	0.7	0.011	*	0.9	0.350		0.9	0.091		1.3	0.049	*	0.9	0.249	
XC0040	9.8E-04	8.4E-04	6.4E-04	5.3E-04	4.8E-04	6.3E-04	1.2E-03	9.2E-04	1.1E-03	9.0E-04	7.0E-04	9.8E-04	8.2E-04	1.8E-04	5.5E-04	7.5E-05	1.1E-03	1.6E-04	8.6E-04	1.4E-04	0.7	0.095		1.3	0.138		1.0	0.797		1.6	0.042	*	0.8	0.153	
Trimethylamine N-oxide	9.0E-03	5.8E-03	3.3E-03	4.5E-03	4.2E-03	3.2E-03	9.9E-03	1.7E-02	7.4E-03	4.5E-03	4.2E-03	7.0E-03	6.1E-03	2.8E-03	4.0E-03	6.9E-04	1.1E-02	4.8E-03	5.2E-03	1.5E-03	0.7	0.328		1.9	0.192		0.9	0.691		1.3	0.285		0.5	0.149	
Trimethylamine	4.4E-04	6.9E-04	N.D.	3.7E-04	3.2E-04	3.4E-04	6.8E-04	7.5E-04	7.3E-04	4.5E-04	4.9E-04	2.0E-03	5.6E-04	1.8E-04	3.4E-04	2.1E-05	7.2E-04	3.3E-05	9.7E-04	8.7E-04	0.6	0.330		1.3	0.419		1.7	0.503		2.8	0.337		1.3	0.667	
7,8-Dihydrobiopterin	1.5E-04	1.4E-04	1.4E-04	1.1E-04	5.6E-05	8.9E-05	1.2E-04	1.3E-04	8.3E-05	1.1E-04	1.2E-04	9.6E-05	1.4E-04	7.2E-06	8.6E-05	2.8E-05	1.1E-04	2.4E-05	1.1E-04	1.4E-05	0.6	0.069		0.8	0.148		0.8	0.037	*	1.3	0.288		1.0	0.990	
N_{ω}-Methylarginine	1.2E-04	1.9E-04	1.4E-04	9.4E-05	9.7E-05	8.0E-05	2.2E-04	1.8E-04	2.1E-04	1.8E-04	1.0E-04	2.5E-04	1.5E-04	3.6E-05	9.0E-05	9.0E-06	2.1E-04	1.9E-05	1.8E-04	7.1E-05	0.6	0.088		1.4	0.100		1.2	0.621		2.0	0.168		0.9	0.559	
N^6-Methyllysine	6.1E-04	6.8E-04	7.3E-04	4.2E-04	3.7E-04	3.9E-04	1.1E-03	9.1E-04	1.0E-03	7.5E-04	5.4E-04	8.3E-04	6.7E-04	6.2E-05	3.9E-04	2.2E-05	1.0E-03	8.5E-05	7.1E-04	1.5E-04	0.6	0.009	**	1.5	0.007	**	1.1	0.739		1.8	0.065		0.7	0.055	
Ophthalmic acid	1.6E-03	2.0E-03	1.3E-03	9.2E-04	8.3E-04	1.1E-03	3.0E-03	1.9E-03	1.7E-03	1.7E-03	1.2E-03	1.8E-03	1.6E-03	3.4E-04	9.4E-04	1.3E-04	2.2E-03	7.2E-04	1.6E-03	3.1E-04	0.6	0.061		1.4	0.289		1.0	0.813		1.6	0.061		0.7	0.246	

Methionine sulfoxide	1.6E-03	2.2E-03	1.7E-03	1.1E-03	1.1E-03	9.2E-04	3.3E-03	3.1E-03	2.8E-03	2.3E-03	2.2E-03	2.9E-03	1.8E-03	3.1E-04	1.0E-03	9.9E-05	3.1E-03	2.2E-04	2.4E-03	3.8E-04
Butyrylcarnitine	1.1E-02	1.1E-02	7.1E-03	7.7E-03	3.1E-03	5.0E-03	4.0E-02	2.0E-02	5.4E-03	1.7E-02	1.4E-02	1.9E-02	9.6E-03	2.2E-03	5.3E-03	2.3E-03	2.2E-02	1.7E-02	1.7E-02	2.3E-03
Glutaric acid	1.3E-03	1.1E-03	6.1E-04	N.D.	N.D.	5.3E-04	1.9E-03	1.3E-03	8.7E-04	1.2E-03	1.0E-03	1.4E-03	1.0E-03	3.7E-04	5.3E-04	N.A.	1.4E-03	5.3E-04	1.2E-03	2.1E-04
4-Acetamidobutanoic acid	1.2E-03	1.6E-03	8.4E-04	9.0E-04	4.2E-04	5.7E-04	4.0E-03	1.9E-03	4.6E-04	1.6E-03	1.7E-03	1.8E-03	1.2E-03	4.0E-04	6.3E-04	2.4E-04	2.1E-03	1.8E-03	1.7E-03	9.4E-05
Glucuronic acid	4.5E-03	6.3E-03	5.1E-03	2.8E-03	1.6E-03	3.4E-03	6.7E-03	6.3E-03	6.9E-03	4.7E-03	4.9E-03	4.2E-03	5.3E-03	9.3E-04	2.6E-03	8.9E-04	6.7E-03	3.1E-04	4.6E-03	3.5E-04
Isovalerylcarnitine	3.4E-03	3.1E-03	2.5E-03	1.9E-03	7.9E-04	1.5E-03	4.2E-03	4.1E-03	2.7E-03	3.7E-03	3.3E-03	4.4E-03	3.0E-03	4.5E-04	1.4E-03	5.6E-04	3.7E-03	8.3E-04	3.8E-03	5.6E-04
Penicillamine	3.0E-04	3.4E-04	2.5E-04	N.D.	1.3E-04	N.D.	4.2E-04	3.9E-04	3.8E-04	2.9E-04	3.4E-04	4.3E-04	2.9E-04	4.2E-05	1.3E-04	N.A.	4.0E-04	2.0E-05	3.5E-04	7.1E-05
UDP-glucuronic acid	7.2E-04	9.2E-04	7.7E-04	3.0E-04	3.1E-04	4.0E-04	2.3E-03	1.5E-03	1.7E-03	5.1E-04	4.8E-04	2.3E-04	8.0E-04	1.0E-04	3.4E-04	5.3E-05	1.8E-03	3.9E-04	4.1E-04	1.5E-04
Octanoylcarnitine	3.5E-04	4.9E-04	2.6E-04	2.0E-04	1.0E-04	1.5E-04	1.8E-03	5.9E-04	1.2E-04	6.1E-04	4.7E-04	6.9E-04	3.7E-04	1.2E-04	1.5E-04	4.8E-05	8.5E-04	8.9E-04	5.9E-04	1.1E-04
Hexanoic acid	N.D.	6.1E-04	N.D.	N.D.	N.D.	N.D.	1.2E-03	6.5E-04	N.D.	4.8E-04	N.D.	7.1E-04	6.1E-04	N.A.	N.D.	N.A.	9.4E-04	4.1E-04	6.0E-04	1.7E-04
Octanoic acid	N.D.	4.0E-04	N.D.	N.D.	N.D.	N.D.	6.8E-04	3.8E-04	N.D.	N.D.	3.6E-04	4.8E-04	4.0E-04	N.A.	N.D.	N.A.	5.3E-04	2.1E-04	4.2E-04	8.8E-05
Methylguanidine	4.2E-04	5.2E-04	N.D.	N.D.	N.D.	N.D.	N.D.	4.0E-04	5.0E-04	4.6E-04	N.D.	5.1E-04	4.7E-04	7.5E-05	N.D.	N.A.	4.5E-04	7.3E-05	4.9E-04	3.1E-05
N-Methylglutamic acid	1.0E-04	8.1E-05	N.D.	N.D.	N.D.	N.D.	N.D.	9.1E-05	8.5E-05	8.9E-05	8.8E-05	9.4E-05	9.3E-05	1.7E-05	N.D.	N.A.	8.8E-05	4.0E-06	9.1E-05	3.3E-06
Ethyl glucuronide	N.D.	7.3E-04	N.D.	N.D.	N.D.	N.D.	3.9E-04	N.D.	9.3E-04	N.D.	N.D.	4.7E-04	7.3E-04	N.A.	N.D.	N.A.	6.6E-04	3.8E-04	4.7E-04	N.A.
3-Amino-2-piperidone	N.D.	2.0E-04	N.D.	N.D.	N.D.	N.D.	N.D.	N.D.	1.5E-04	N.D.	N.D.	2.8E-04	2.0E-04	N.A.	N.D.	N.A.	1.5E-04	N.A.	2.8E-04	N.A.
N-Methylproline	N.D.	N.D.	N.D.	N.D.	N.D.	N.D.	2.4E-04	2.0E-04	2.0E-04	2.1E-04	1.7E-04	2.5E-04	N.D.	N.A.	N.D.	N.A.	2.1E-04	2.1E-05	2.1E-04	3.9E-05
Isobutyric acid	N.D.	N.D.	N.D.	N.D.	N.D.	N.D.	7.4E-04	5.8E-04	N.D.	4.4E-04	N.D.	N.D.	N.D.	N.A.	N.D.	N.A.	6.6E-04	1.1E-04	4.4E-04	N.A.
S-Methylmethionine	N.D.	N.D.	N.D.	N.D.	N.D.	N.D.	N.D.	N.D.	N.D.	5.2E-05	4.9E-05	5.7E-05	N.D.	N.A.	N.D.	N.A.	N.D.	N.A.	5.2E-05	4.3E-06

Methionine sulfoxide	0.6	0.037	*	1.7	0.007	**	1.3	0.107		2.4	0.018	*	0.8	0.085	
Butyrylcarnitine	0.6	0.079		2.3	0.353		1.7	0.020	*	3.1	0.004	**	0.8	0.661	
Glutaric acid	0.5	N.A.		1.3	0.423		1.2	0.446		2.3	N.A.		0.9	0.731	
4-Acetamidobutanoic acid	0.5	0.104		1.7	0.482		1.4	0.172		2.7	0.009	**	0.8	0.723	
Glucuronic acid	0.5	0.021	*	1.3	0.116		0.9	0.329		1.8	0.043	*	0.7	0.002	**
Isovalerylcarnitine	0.5	0.020	*	1.2	0.287		1.3	0.124		2.7	0.006	**	1.0	0.859	
Penicillamine	0.4	N.A.		1.3	0.036	*	1.2	0.291		2.7	N.A.		0.9	0.420	
UDP-glucuronic acid	0.4	0.006	**	2.3	0.037	*	0.5	0.025	*	1.2	0.523		0.2	0.014	*
Octanoylcarnitine	0.4	0.070		2.3	0.445		1.6	0.074		3.9	0.009	**	0.7	0.661	
Hexanoic acid	<1	N.A.		1.5	N.A.		1.0	N.A.		1<	N.A.		0.6	0.438	
Octanoic acid	<1	N.A.		1.3	N.A.		1.0	N.A.		1<	N.A.		0.8	0.604	
Methylguanidine	<1	N.A.		1.0	0.832		1.0	0.810		1<	N.A.		1.1	0.626	
N-Methylglutamic acid	<1	N.A.		1.0	0.779		1.0	0.893		1<	N.A.		1.0	0.577	
Ethyl glucuronide	<1	N.A.		0.9	N.A.		0.7	N.A.		1<	N.A.		0.7	N.A.	
3-Amino-2-piperidone	<1	N.A.		0.7	N.A.		1.4	N.A.		1<	N.A.		1.9	N.A.	
N-Methylproline	N.A.	N.A.		1<	N.A.		1<	N.A.		1<	N.A.		1.0	0.929	
Isobutyric acid	N.A.	N.A.		1<	N.A.		1<	N.A.		1<	N.A.		0.7	N.A.	
S-Methylmethionine	N.A.	N.A.		N.A.	N.A.		1<	N.A.		1<	N.A.		1<	N.A.	

N.D.: Not Detected.
N.A.: Not Available.
† candidate compound is predicted from HMT database based on m/z and MT of peaks.

¶ in the case of A vs B, the ratio of the average was calculated as A/B.

|| Welch's *t* -test , *p* -value (*<0.05, **<0.01, ***<0.001)
The peak signal intensities of Ala and Betaine were saturated and the relative areas of ^{13}C were used.
Description of XA・・・or XC・・・ in compound name indicates unknown peaks, which has been detected in some other organism samples in the HMT database for metabolite identification.
