# Supplementary material for: Chronic Intake of Japanese Sake Mediates Radiation-Induced Metabolic Alterations in Mouse Liver
Source: PLoS One. 2016 Jan 11;11(1):e0146730. doi: 10.1371/journal.pone.0146730 (PMC4713437; doi:10.1371/journal.pone.0146730)
Supplement: S5 Table — These have significant correlation (P<0.01) based on factor loadings between the PC2 scores and variables in the metabolome data for irradiated mice administered sake. (PDF) [file pone.0146730.s008.pdf]

S5 Table.Selected metabolites.

These have significant correlation (P<0.01) based on factor loadings between the PC2 scores and variables in the metabolome data for irradiated mice administered sake.

Sample No	1	2	3	4	5	6	7	8	9	10	11	12		
PC2 scores	−4.64	−5.89	−2.02	−7.94	1.44	1.48	−5.19	−5.08	−1.51	11.88	9.03	8.45		
Metabolites	Relative area values												factor loading	p-value
XA0013	N.D.	N.D.	N.D.	1.7E−04	N.D.	N.D.	N.D.	N.D.	4.6E−04	N.D.	N.D.	N.D.	1.0E+00	1.3E−79
Cytosine	N.D.	N.D.	N.D.	N.D.	N.D.	N.D.	4.3E−05	5.7E−05	8.7E−05	N.D.	N.D.	N.D.	9.5E−01	1.4E−06
AMP	1.1E−01	1.1E−01	1.2E−01	9.7E−02	1.2E−01	1.2E−01	1.2E−01	1.2E−01	1.2E−01	1.7E−01	1.6E−01	1.6E−01	9.5E−01	2.5E−06
Gly−Gly	4.0E−04	4.0E−04	3.8E−04	3.5E−04	4.5E−04	3.9E−04	3.9E−04	4.2E−04	4.5E−04	7.5E−04	6.1E−04	6.5E−04	9.3E−01	1.3E−05
3'−Dephospho CoA	1.8E−04	1.2E−04	1.7E−04	2.0E−04	2.0E−04	2.8E−04	1.6E−04	2.0E−04	2.7E−04	4.8E−04	3.8E−04	4.8E−04	9.1E−01	3.1E−05
Cys	1.6E−04	1.3E−04	1.4E−04	2.0E−04	2.2E−04	2.6E−04	1.5E−04	2.4E−04	2.3E−04	4.2E−04	4.5E−04	4.0E−04	9.1E−01	4.5E−05
Glutathione (GSH)	3.8E−01	3.4E−01	4.5E−01	4.5E−01	5.0E−01	5.1E−01	4.0E−01	3.9E−01	5.0E−01	5.8E−01	6.4E−01	5.5E−01	9.0E−01	6.7E−05
Ala−Ala	7.2E−04	6.1E−04	7.2E−04	4.9E−04	7.3E−04	7.4E−04	7.2E−04	7.5E−04	8.1E−04	9.6E−04	9.1E−04	9.3E−04	8.9E−01	1.1E−04
Nicotinamide	6.2E−02	6.3E−02	7.6E−02	7.8E−02	8.5E−02	7.6E−02	7.6E−02	8.3E−02	6.5E−02	1.2E−01	1.2E−01	1.2E−01	8.9E−01	1.3E−04
Trimethylamine	6.2E−04	9.1E−04	8.6E−04	3.6E−04	8.2E−04	6.6E−04	1.5E−03	9.6E−04	1.3E−03	3.0E−03	3.5E−03	3.3E−03	8.6E−01	3.3E−04
Cysteine glutathione (	1.6E−04	1.4E−04	1.3E−04	1.3E−04	1.8E−04	1.5E−04	1.7E−04	1.9E−04	2.1E−04	2.9E−04	2.5E−04	2.5E−04	8.5E−01	4.5E−04
CMP	7.5E−04	8.9E−04	7.0E−04	5.6E−04	9.8E−04	9.2E−04	8.1E−04	7.9E−04	7.4E−04	1.1E−03	1.1E−03	1.0E−03	8.4E−01	5.6E−04
GMP	9.9E−03	8.4E−03	9.7E−03	6.5E−03	9.5E−03	8.6E−03	9.4E−03	1.0E−02	1.1E−02	1.2E−02	1.2E−02	1.3E−02	8.4E−01	6.7E−04
Gly	1.2E−01	1.1E−01	1.2E−01	1.2E−01	1.5E−01	1.5E−01	1.1E−01	1.3E−01	1.2E−01	1.6E−01	1.4E−01	1.4E−01	8.3E−01	8.1E−04
Octanoic acid	N.D.	N.D.	N.D.	N.D.	N.D.	1.3E−04	3.8E−04	5.4E−04	4.2E−04	N.D.	N.D.	N.D.	−8.3E−01	9.0E−04
6−Phosphogluconic ac	1.7E−03	1.8E−03	2.0E−03	1.7E−03	2.0E−03	2.2E−03	1.3E−03	1.7E−03	1.6E−03	2.2E−03	2.4E−03	2.1E−03	8.2E−01	1.1E−03
Ascorbic acid	1.6E−02	2.0E−02	2.3E−02	2.0E−02	2.8E−02	3.6E−02	1.5E−02	1.7E−02	2.7E−02	3.6E−02	3.1E−02	2.8E−02	8.1E−01	1.3E−03
CoA_divalent	5.5E−03	4.2E−03	6.2E−03	5.9E−03	6.1E−03	7.6E−03	5.1E−03	6.3E−03	7.1E−03	8.0E−03	8.0E−03	7.3E−03	8.1E−01	1.4E−03
XC0137	5.6E−03	5.5E−03	6.5E−03	5.3E−03	5.8E−03	5.6E−03	5.6E−03	5.2E−03	6.0E−03	2.9E−03	3.2E−03	3.3E−03	−8.1E−01	1.5E−03
XC0016	9.2E−04	7.4E−04	8.4E−04	6.4E−04	1.0E−03	9.5E−04	7.2E−04	6.7E−04	7.6E−04	1.1E−03	9.8E−04	8.8E−04	7.9E−01	2.4E−03
Sedoheptulose 7−pho	7.5E−03	5.4E−03	5.3E−03	4.0E−03	3.5E−03	3.8E−03	6.0E−03	6.1E−03	6.8E−03	2.3E−03	2.2E−03	2.2E−03	−7.9E−01	2.5E−03
Thiamine phosphate	8.1E−04	7.9E−04	8.8E−04	6.8E−04	8.6E−04	1.0E−03	6.5E−04	5.4E−04	7.3E−04	9.8E−04	9.6E−04	1.0E−03	7.8E−01	2.5E−03
S−Methylglutathione	1.9E−03	1.6E−03	1.5E−03	1.5E−03	2.1E−03	1.8E−03	1.5E−03	1.3E−03	2.0E−03	2.2E−03	2.1E−03	2.0E−03	7.8E−01	2.6E−03
Lactic acid	4.0E−01	4.0E−01	4.8E−01	4.6E−01	5.4E−01	5.6E−01	4.4E−01	4.0E−01	4.7E−01	5.4E−01	5.8E−01	5.2E−01	7.8E−01	2.7E−03
UMP	1.9E−02	1.9E−02	2.0E−02	1.1E−02	1.7E−02	1.5E−02	1.9E−02	2.0E−02	1.9E−02	2.5E−02	2.5E−02	2.6E−02	7.7E−01	3.6E−03
XC0132	5.6E−04	5.9E−04	6.5E−04	5.7E−04	6.8E−04	6.4E−04	5.2E−04	5.6E−04	5.7E−04	6.5E−04	6.9E−04	6.6E−04	7.6E−01	4.0E−03
Glucose 6−phosphate	3.5E−02	2.2E−02	3.1E−02	3.4E−02	2.4E−02	2.9E−02	3.1E−02	3.4E−02	3.5E−02	1.2E−02	2.5E−02	2.0E−02	−7.6E−01	4.5E−03
Ribose 5−phosphate	3.1E−03	2.9E−03	4.3E−03	3.4E−03	4.2E−03	3.6E−03	2.4E−03	3.1E−03	3.4E−03	4.5E−03	4.2E−03	3.9E−03	7.6E−01	4.5E−03
IMP	3.9E−03	5.0E−03	4.5E−03	4.1E−03	2.8E−03	3.0E−03	5.0E−03	5.3E−03	4.9E−03	3.5E−03	2.5E−03	2.4E−03	−7.4E−01	5.4E−03
Glycerol 3−phosphate	7.4E−02	6.7E−02	7.6E−02	8.2E−02	7.3E−02	8.1E−02	6.3E−02	6.8E−02	7.4E−02	8.7E−02	9.1E−02	8.1E−02	7.3E−01	7.3E−03
Gly−Asp	2.1E−04	2.0E−04	2.5E−04	1.8E−04	2.8E−04	2.1E−04	2.0E−04	1.9E−04	2.3E−04	2.7E−04	2.3E−04	2.4E−04	7.1E−01	9.7E−03
