# Supplementary material for: Chronic Intake of Japanese Sake Mediates Radiation-Induced Metabolic Alterations in Mouse Liver
Source: PLoS One. 2016 Jan 11;11(1):e0146730. doi: 10.1371/journal.pone.0146730 (PMC4713437; doi:10.1371/journal.pone.0146730)
Supplement: S6 Table — These have significant correlation (P<0.01) based on factor loadings between the PC1 scores and variables in the metabolome data for irradiated mice administered sake. (PDF) [file pone.0146730.s009.pdf]

S6 Table.Selected metabolites.

These have significant correlation (P<0.01) based on factor loadings between the PC1 scores and variables in the metabolome data for irradiated mice administered sake.

Sample No	1	2	3	4	5	6	7	8	9	10	11	12		
PC1 scores	−11.30	−3.14	−3.82	17.52	5.40	13.07	−5.82	−4.89	−4.96	−3.51	0.39	1.05		
Metabolites	Relative area values												factor loading	p-value
Val	1.3E−01	1.1E−01	1.1E−01	7.8E−02	9.6E−02	7.8E−02	1.1E−01	1.1E−01	1.2E−01	1.1E−01	1.1E−01	1.1E−01	−9.7E−01	1.1E−07
Urea	5.6E−01	5.5E−01	5.5E−01	3.3E−01	4.2E−01	3.2E−01	5.4E−01	5.3E−01	5.2E−01	4.9E−01	5.2E−01	4.8E−01	−9.5E−01	2.7E−06
XA0027	4.2E−04	2.4E−04	N.D.	N.D.	2.0E−04	N.D.	N.D.	N.D.	N.D.	N.D.	N.D.	N.D.	−9.4E−01	7.8E−06
Homoserine	4.1E−04	3.1E−04	3.5E−04	1.5E−04	2.6E−04	2.4E−04	3.6E−04	3.3E−04	3.3E−04	2.9E−04	3.0E−04	2.6E−04	−9.4E−01	8.1E−06
Pipecolic acid	3.6E−03	2.5E−03	2.9E−03	1.4E−03	1.9E−03	1.4E−03	2.7E−03	2.3E−03	2.9E−03	2.9E−03	2.4E−03	2.0E−03	−9.3E−01	1.6E−05
5,6,7,8−Tetrahydr	N.D.	N.D.	1.2E−04	N.D.	N.D.	1.9E−04	N.D.	N.D.	1.1E−04	1.4E−04	1.4E−04	1.2E−04	9.2E−01	2.0E−05
Stachydrine	3.0E−02	2.8E−02	3.0E−02	1.1E−02	2.1E−02	1.2E−02	3.1E−02	2.9E−02	2.8E−02	2.8E−02	3.3E−02	2.6E−02	−9.2E−01	3.0E−05
N6−Acetyllysine	4.9E−04	5.2E−04	4.8E−04	2.0E−04	3.2E−04	2.7E−04	4.7E−04	4.0E−04	4.2E−04	4.1E−04	3.5E−04	3.7E−04	−9.1E−01	3.2E−05
5−Aminovaleric ac	1.2E−02	1.1E−02	1.4E−02	3.8E−03	7.6E−03	4.6E−03	1.2E−02	1.1E−02	1.3E−02	8.3E−03	8.9E−03	8.6E−03	−9.0E−01	5.9E−05
N−Acetyllysine	2.0E−03	1.9E−03	2.3E−03	1.0E−03	1.8E−03	1.4E−03	2.1E−03	2.1E−03	2.1E−03	2.0E−03	1.8E−03	1.9E−03	−9.0E−01	8.4E−05
Octanoic acid	N.D.	N.D.	N.D.	N.D.	N.D.	1.3E−04	3.8E−04	5.4E−04	4.2E−04	N.D.	N.D.	N.D.	−8.9E−01	8.8E−05
Betaine	7.1E−01	6.6E−01	8.2E−01	2.7E−01	4.8E−01	2.9E−01	8.0E−01	6.4E−01	6.0E−01	5.8E−01	6.8E−01	6.1E−01	−8.9E−01	1.1E−04
2−Aminobutyric ac	1.0E−02	5.5E−03	6.1E−03	2.9E−03	4.6E−03	4.0E−03	6.6E−03	6.1E−03	7.6E−03	6.9E−03	6.0E−03	5.2E−03	−8.9E−01	1.1E−04
XA0012	1.2E−03	1.0E−03	1.0E−03	5.5E−04	7.8E−04	6.1E−04	1.0E−03	1.1E−03	1.0E−03	9.0E−04	6.5E−04	7.2E−04	−8.9E−01	1.3E−04
Galacturonic acid	5.1E−04	3.8E−04	4.1E−04	2.1E−04	3.5E−04	3.1E−04	5.2E−04	5.1E−04	4.6E−04	4.5E−04	4.3E−04	4.9E−04	−8.8E−01	1.3E−04
Lys	1.6E−01	1.4E−01	1.6E−01	8.6E−02	9.6E−02	8.2E−02	1.2E−01	1.2E−01	1.3E−01	1.4E−01	1.2E−01	1.2E−01	−8.8E−01	1.4E−04
Hydroxyproline	1.5E−02	8.8E−03	9.4E−03	4.8E−03	8.1E−03	6.0E−03	1.1E−02	8.4E−03	1.2E−02	1.2E−02	9.8E−03	9.8E−03	−8.8E−01	1.4E−04
GABA	4.2E−03	3.2E−03	4.0E−03	2.0E−03	3.1E−03	2.5E−03	3.7E−03	3.8E−03	3.7E−03	4.5E−03	3.9E−03	3.8E−03	−8.7E−01	2.0E−04
Betonicine	5.5E−04	4.7E−04	6.7E−04	1.3E−04	2.8E−04	1.6E−04	5.7E−04	6.1E−04	5.1E−04	3.8E−04	5.6E−04	4.2E−04	−8.7E−01	2.1E−04
N−Acetylglutamic	7.7E−03	5.7E−03	5.8E−03	2.2E−03	3.5E−03	3.0E−03	4.4E−03	4.7E−03	5.0E−03	4.6E−03	4.0E−03	4.1E−03	−8.7E−01	2.5E−04
S−Adenosylhomoc	4.9E−03	3.9E−03	3.0E−03	2.5E−03	2.7E−03	2.2E−03	4.2E−03	4.6E−03	4.3E−03	4.5E−03	3.2E−03	3.4E−03	−8.6E−01	3.0E−04
3−Methylhistidine	2.8E−03	2.0E−03	2.5E−03	1.2E−03	1.5E−03	1.2E−03	2.4E−03	2.0E−03	2.9E−03	1.8E−03	1.9E−03	1.6E−03	−8.6E−01	3.1E−04
XC0061	6.5E−03	6.3E−03	7.2E−03	2.5E−03	5.3E−03	4.9E−03	6.6E−03	7.2E−03	7.4E−03	7.8E−03	6.4E−03	5.1E−03	−8.6E−01	3.5E−04
Methionine sulfoxi	1.8E−03	1.1E−03	1.5E−03	6.8E−04	8.8E−04	7.6E−04	1.7E−03	1.8E−03	1.4E−03	1.3E−03	1.6E−03	1.1E−03	−8.6E−01	3.8E−04
γ−Butyrobetaine	6.2E−03	6.8E−03	8.0E−03	2.7E−03	4.5E−03	3.3E−03	6.3E−03	5.6E−03	5.8E−03	5.8E−03	5.1E−03	4.9E−03	−8.5E−01	4.0E−04
Sarcosine	3.4E−02	2.5E−02	2.2E−02	9.5E−03	2.1E−02	1.7E−02	3.1E−02	2.5E−02	3.1E−02	3.7E−02	2.6E−02	2.9E−02	−8.5E−01	4.5E−04
3−Hydroxybutyric	1.8E−02	1.5E−02	1.4E−02	8.3E−03	8.8E−03	8.4E−03	1.6E−02	1.7E−02	1.5E−02	1.1E−02	9.9E−03	1.0E−02	−8.5E−01	5.0E−04
2−Methylserine	3.1E−04	2.3E−04	2.7E−04	N.D.	1.9E−04	1.2E−04	2.8E−04	3.4E−04	3.3E−04	2.3E−04	2.2E−04	1.7E−04	−8.5E−01	5.1E−04
UDP−glucoseUDP	7.8E−03	1.1E−02	1.4E−02	2.4E−02	2.9E−02	3.6E−02	9.5E−03	8.9E−03	1.2E−02	1.9E−02	1.6E−02	1.8E−02	8.4E−01	5.7E−04
Phosphoenolpyruv	4.6E−04	5.4E−04	6.8E−04	8.5E−04	8.7E−04	9.0E−04	3.8E−04	4.5E−04	4.0E−04	5.7E−04	5.2E−04	7.5E−04	8.4E−01	6.6E−04
Phosphorylcholine	2.1E−01	1.9E−01	1.9E−01	5.5E−02	1.4E−01	1.1E−01	1.4E−01	1.4E−01	1.7E−01	1.9E−01	1.4E−01	1.4E−01	−8.4E−01	6.8E−04
3−Phosphoglyceri	1.0E−03	1.2E−03	1.4E−03	1.8E−03	1.7E−03	2.1E−03	6.9E−04	9.2E−04	8.7E−04	1.2E−03	1.2E−03	1.6E−03	8.4E−01	6.9E−04
XC0040	6.1E−04	7.4E−04	8.0E−04	1.6E−04	4.2E−04	2.2E−04	7.4E−04	7.3E−04	5.6E−04	4.8E−04	5.4E−04	6.8E−04	−8.4E−01	6.9E−04
cis−4−Hydroxypro	3.7E−04	2.4E−04	2.6E−04	1.9E−04	1.7E−04	1.7E−04	2.6E−04	2.5E−04	2.8E−04	2.9E−04	2.8E−04	2.3E−04	−8.3E−01	7.9E−04
XA0033	3.7E−02	3.4E−02	3.5E−02	1.1E−02	2.6E−02	2.2E−02	2.5E−02	2.7E−02	3.1E−02	3.6E−02	2.6E−02	2.7E−02	−8.3E−01	8.1E−04
SDMA	1.8E−04	1.5E−04	1.4E−04	1.0E−04	1.4E−04	9.6E−05	1.2E−04	1.5E−04	1.5E−04	1.3E−04	1.2E−04	1.4E−04	−8.2E−01	1.1E−03
Trigonelline	5.6E−03	5.0E−03	4.6E−03	1.8E−03	4.0E−03	2.5E−03	4.8E−03	4.2E−03	5.1E−03	6.3E−03	5.2E−03	5.8E−03	−8.2E−01	1.1E−03
Glucuronic acid	4.9E−03	5.4E−03	5.6E−03	2.7E−03	4.5E−03	3.5E−03	6.1E−03	6.0E−03	5.6E−03	5.7E−03	5.8E−03	6.1E−03	−8.2E−01	1.2E−03
N6−Methyllysine	5.9E−04	3.9E−04	3.9E−04	1.9E−04	1.7E−04	1.3E−04	5.1E−04	4.7E−04	4.9E−04	2.0E−04	2.9E−04	2.3E−04	−8.1E−01	1.4E−03
UDP−glucuronic a	4.8E−03	4.4E−03	5.9E−03	3.5E−04	2.0E−03	6.6E−04	4.3E−03	3.7E−03	2.7E−03	1.8E−03	3.2E−03	3.5E−03	−8.1E−01	1.5E−03
Ophthalmic acid	1.8E−03	1.3E−03	1.4E−03	8.3E−04	1.4E−03	1.2E−03	1.8E−03	1.3E−03	1.4E−03	1.4E−03	1.4E−03	1.3E−03	−8.1E−01	1.5E−03

Gln	2.5E-01	2.3E-01	2.2E-01	3.5E-01	2.6E-01	2.8E-01	2.1E-01	2.4E-01	2.1E-01	2.7E-01	2.6E-01	2.6E-01	8.1E-01	1.6E-03
Ile	8.0E-02	7.0E-02	6.2E-02	5.9E-02	6.8E-02	5.6E-02	7.2E-02	7.2E-02	7.6E-02	7.5E-02	6.2E-02	6.7E-02	-8.0E-01	1.6E-03
1-Methylnicotinan	7.7E-04	5.2E-04	5.5E-04	3.7E-04	2.4E-04	2.9E-04	5.5E-04	6.4E-04	5.1E-04	5.9E-04	3.9E-04	3.8E-04	-8.0E-01	1.7E-03
γ-Glu-Cys	8.4E-04	4.6E-04	6.9E-04	4.5E-04	4.1E-04	4.3E-04	6.5E-04	6.9E-04	6.8E-04	7.7E-04	6.1E-04	5.7E-04	-8.0E-01	1.9E-03
N-Acetylhistidine	9.5E-04	7.6E-04	9.0E-04	5.5E-04	6.5E-04	6.6E-04	8.4E-04	7.5E-04	6.8E-04	7.8E-04	6.2E-04	7.0E-04	-8.0E-01	2.0E-03
Met	1.4E-02	1.1E-02	1.1E-02	9.7E-03	8.2E-03	7.3E-03	1.2E-02	1.2E-02	1.1E-02	1.3E-02	1.1E-02	9.3E-03	-7.9E-01	2.4E-03
N-Acetylserine	3.7E-04	3.3E-04	2.8E-04	2.1E-04	2.8E-04	2.2E-04	2.7E-04	3.0E-04	2.5E-04	3.1E-04	2.5E-04	2.7E-04	-7.9E-01	2.4E-03
Ornithine	9.2E-02	8.9E-02	9.2E-02	4.3E-02	7.9E-02	5.4E-02	7.4E-02	7.5E-02	6.7E-02	8.9E-02	8.5E-02	8.6E-02	-7.8E-01	3.0E-03
Isovalerylalanine	4.2E-04	3.3E-04	2.3E-04	1.9E-04	2.7E-04	2.1E-04	3.3E-04	3.0E-04	3.8E-04	4.1E-04	2.4E-04	3.0E-04	-7.7E-01	3.5E-03
Leu	1.4E-01	1.2E-01	1.1E-01	1.1E-01	1.2E-01	1.0E-01	1.2E-01	1.2E-01	1.3E-01	1.4E-01	1.2E-01	1.3E-01	-7.6E-01	4.0E-03
Gluconolactone	6.6E-03	5.9E-03	6.7E-03	3.7E-03	5.5E-03	5.3E-03	6.5E-03	6.5E-03	7.4E-03	8.1E-03	7.2E-03	6.7E-03	-7.6E-01	4.1E-03
Imidazolelactic ac	3.2E-04	3.5E-04	3.4E-04	9.8E-05	2.6E-04	1.8E-04	2.7E-04	2.0E-04	3.5E-04	3.0E-04	2.6E-04	3.4E-04	-7.6E-01	4.4E-03
2-Hydroxybutyric	2.9E-03	2.0E-03	2.4E-03	9.9E-04	1.9E-03	2.0E-03	2.6E-03	2.8E-03	3.7E-03	3.3E-03	2.9E-03	2.6E-03	-7.5E-01	4.5E-03
3-Aminoisobutyric	2.6E-03	1.9E-03	1.6E-03	1.2E-03	1.6E-03	1.0E-03	1.5E-03	1.7E-03	1.4E-03	1.7E-03	1.6E-03	1.7E-03	-7.5E-01	4.8E-03
XA0035	4.3E-04	4.3E-04	4.4E-04	5.7E-04	6.3E-04	7.5E-04	3.1E-04	4.1E-04	4.3E-04	5.4E-04	5.1E-04	4.2E-04	7.5E-01	4.8E-03
XA0055	2.5E-04	2.4E-04	4.7E-04	2.0E-03	6.2E-04	3.9E-04	N.D.	3.0E-04	2.4E-04	1.6E-04	8.5E-04	6.0E-04	7.4E-01	6.3E-03
5'-Deoxy-5'-meth	2.7E-04	1.9E-04	1.7E-04	1.2E-04	1.5E-04	1.3E-04	1.9E-04	1.6E-04	1.6E-04	1.4E-04	1.6E-04	1.4E-04	-7.3E-01	6.9E-03
N5-Ethylglutamine	3.8E-04	3.7E-04	5.3E-04	2.4E-04	3.3E-04	3.0E-04	5.8E-04	5.5E-04	5.2E-04	4.5E-04	4.8E-04	4.1E-04	-7.3E-01	7.0E-03
Ribulose 5-phosp	6.0E-03	5.8E-03	6.2E-03	9.4E-03	6.8E-03	8.2E-03	5.9E-03	6.5E-03	6.2E-03	8.8E-03	7.6E-03	7.8E-03	7.3E-01	7.0E-03
N-Acetylglutamine	6.1E-04	5.4E-04	6.5E-04	8.0E-04	6.7E-04	7.7E-04	5.8E-04	6.4E-04	4.9E-04	7.2E-04	5.8E-04	6.2E-04	7.3E-01	7.2E-03
N-Methylalanine	2.3E-04	1.6E-04	1.4E-04	N.D.	N.D.	N.D.	N.D.	1.1E-04	N.D.	1.4E-04	1.4E-04	N.D.	-7.3E-01	7.3E-03
Homocitrulline	2.3E-04	2.0E-04	1.8E-04	1.5E-04	1.8E-04	1.3E-04	2.4E-04	2.3E-04	2.1E-04	1.5E-04	1.8E-04	1.3E-04	-7.2E-01	8.5E-03
Isovalerylcarnitine	2.6E-03	2.1E-03	2.2E-03	4.1E-04	2.9E-03	1.8E-03	3.7E-03	3.3E-03	4.4E-03	3.2E-03	2.5E-03	2.7E-03	-7.2E-01	8.6E-03
Cystathionine	1.2E-02	7.1E-03	5.8E-03	3.7E-03	6.9E-03	6.1E-03	6.6E-03	7.5E-03	8.5E-03	1.0E-02	6.4E-03	7.6E-03	-7.2E-01	8.7E-03
Glutaric acid	7.7E-04	5.1E-04	3.8E-04	1.8E-04	4.4E-04	4.8E-04	7.2E-04	6.0E-04	4.4E-04	7.6E-04	4.8E-04	5.6E-04	-7.2E-01	8.7E-03
Cytosine	N.D.	N.D.	N.D.	N.D.	N.D.	N.D.	4.3E-05	5.7E-05	8.7E-05	N.D.	N.D.	N.D.	7.1E-01	9.8E-03
