# Supplementary material for: Chronic Intake of Japanese Sake Mediates Radiation-Induced Metabolic Alterations in Mouse Liver
Source: PLoS One. 2016 Jan 11;11(1):e0146730. doi: 10.1371/journal.pone.0146730 (PMC4713437; doi:10.1371/journal.pone.0146730)
Supplement: S7 Table — (PDF) [file pone.0146730.s010.pdf]

S7 Table

Changes in food intake during the administration of 15% ethanol.

Period of administration (day number)	Food intake (g/mouse/day)	
	Control (water)	Ethanol
14th-18th	2.64	2.31
19th-22th	2.64	2.55
23th-26th	2.84	2.75
27th-30th	2.68	2.59

Four or five mice were bred per cage. Daily food intake was calculated based on the difference between the weight of food added to the cage in the morning after treatment and the weight of food remaining in the evening.
